# Inactivation of NLRP3 inflammasome by dephosphorylation at Serine 658 alleviates glial inflammation in the mouse model of Parkinson’s disease

**DOI:** 10.1186/s13024-025-00818-z

**Published:** 2025-03-05

**Authors:** Rong-Xin Zhu, Rui-Xue Han, Yue-Han Chen, Lei Huang, Ting Liu, Jingwei Jiang, Cong Wang, Lei Cao, Yang Liu, Ming Lu

**Affiliations:** 1 Jiangsu Key Laboratory of Neurodegeneration, Department of Pharmacology, Nanjing Medical University, Nanjing, 211116 China; 2 Department of Pharmacology, Nanjing University of Chinese Medicine, Nanjing, Jiangsu 210023 China; 3 China Pharmaceutical University, Nanjing, 211116 China

**Keywords:** NLRP3 inflammasome, Phosphorylation, Serine 658, Inflammation, Parkinson’s disease

## Abstract

**Background:**

Parkinson’s disease (PD) is a leading neurodegenerative disorder characterized by the progressive loss of dopaminergic neurons, contributing to considerable disability worldwide. Current treatments offer only symptomatic relief, highlighting the need for novel therapeutic strategies targeting disease progression. Neuroinflammation plays a pivotal role in PD pathogenesis, with the NLRP3 inflammasome emerging as a key contributor.

**Methods:**

The virtual screening of a natural product library comprising 5,088 compounds was applied to identify five potential NLRP3 inhibitors through molecular docking scores. Then surface plasmon resonance assays were used to detect their binding affinities to the NLRP3 protein. Functional studies in macrophages and glial cells were used to demonstrate the effect of Psoralen on NLRP3 phosphorylation and inflammasome activation.

**Results:**

Psoralen treatment improved PD-like symptoms and reduced dopaminergic neuronal death by targeting glial NLRP3 inflammasome activation in the MPTP/p mouse model. By performing 4D label-free quantitative phosphorylation proteomics and site mutation assays, we identified that Psoralen prevents NLRP3 phosphorylation at Serine 658 by binding to its NACHT and LRR domains.

**Conclusions:**

These findings position Psoralen as a promising NLRP3 inflammasome inhibitor, offering a potential therapeutic avenue for PD and other NLRP3 inflammasome-related diseases. Additionally, this research highlights the innovative approach of targeting specific phosphorylation sites on the NLRP3 protein to reduce neuroinflammation.

**Supplementary Information:**

The online version contains supplementary material available at 10.1186/s13024-025-00818-z.

## Background

Parkinson’s disease (PD) is the second most prevalent neurodegenerative disorder, contributing significantly to global disability and public health challenges [1]. The primary pathological hallmark of PD is the progressive loss of dopaminergic (DA) neurons in the substantia nigra, associated with complex pathogenic processes [2]. Current therapeutic strategies, aside from levodopa which provides only symptomatic relief, are unable to slow the disease progression [3]. Neuroinflammation was first reported in PD pathogenesis in 1988, highlighting reactive microglia in post-mortem PD patient tissues [4]. This discovery has spurred numerous drug trials focusing on anti-inflammatory agents [5]. Although no Phase 3 clinical trials have yet confirmed the efficacy of anti-inflammatory strategies for PD therapy, targeting neuroinflammation remains promising and is a primary research focus. Identifying anti-inflammatory targets is crucial for successful clinical translation.

The term “inflammasome” describes a large multimeric protein complex that activates inflammatory caspases and processes pro-IL-β [6]. Inflammasome assembly is triggered by pattern recognition receptors (PRRs) like nucleotide-binding oligomerization domain (NOD)-like receptors (NLRs) and absent in melanoma 2 (AIM2)-like receptors [7]. Five receptors are confirmed to assemble inflammasomes: NLRP1, NLRP3, NLRC4, AIM2, and pyrin [7]. NLRP3 is the most extensively studied among these [8]. Dysregulated NLRP3 inflammasome activation is linked to various diseases, including cancer, metabolic diseases, and neurodegenerative disorders [9, 10]. In PD research, NLRP3 inflammasome activation in glial cells and DA neurons accelerates disease progression [11–13]. Importantly, both our group and others have observed that inhibiting NLRP3 inflammasome assembly and activation offers therapeutic benefits in PD models [12, 14, 15]. These findings suggest that targeting NLRP3 inflammasome could facilitate the development of anti-inflammatory strategies for PD treatment. Natural products, known for their molecular diversity and unique bioactivity, are vital resources for drug discovery, offering enhanced efficacy and safety [16]. Screening for potential NLRP3 inflammasome inhibitors from natural products could expedite clinical translation success.

Canonical NLRP3 inflammasome activation involves a two-step process: (1) A priming signal induced by Toll-like receptor (TLR) ligands, leading to upregulation of NLRP3, pro-IL-1β, and pro-IL-18; (2) An activation step triggered by stimuli such as ATP and nigericin, resulting in inflammasome assembly and IL-1β release [17]. Clinical trials of the IL-1β-targeted monoclonal antibody canakinumab have shown therapeutic effects on inflammatory diseases, including atherosclerosis and recurrent fever syndromes, though higher rates of fatal infections were observed in recipients [18, 19]. This raises the question of whether upstream targeting with NLRP3-specific inhibitors might offer safety and efficacy advantages over IL-1β blockade. Recent studies suggest that post-translational modifications of the NLRP3 protein, such as ubiquitination and phosphorylation, are crucial for inflammasome assembly and activation [8]. For example, phosphorylation of NLRP3 protein at Serine 194 initiates inflammasome activation, while phosphorylation at Serine 803 inhibits it [20, 21]. These insights provide valuable strategies for selectively targeting NLRP3 protein to ameliorate NLRP3 inflammasome-driven neuroinflammation.

In this study, we conducted a virtual screening of a natural product library comprising 5,088 compounds. Five candidate NLRP3 inhibitors were selected for further investigation based on their molecular docking scores. Surface plasmon resonance (SPR) assays were used to determine the binding affinities of these candidates to the NLRP3 protein. Functional studies were performed in bone marrow-derived macrophages (BMDMs) stimulated with LPS + ATP and LPS + Nigericin, two established NLRP3 inflammasome activation models. Among these candidates, S4737, known as Psoralen, emerged as the most effective NLRP3 inflammasome inhibitor, impeding NLRP3 phosphorylation and inflammasome assembly. Notably, this inhibitory effect was validated in microglia and astrocytes, key players in neuroinflammation. Psoralen treatment improved PD-like motor symptoms and prevented DA neuronal death by inhibiting glial NLRP3 inflammasome activation. Mechanistically, Psoralen blocked NLRP3 phosphorylation at Serine 658 by binding to its NACTH and LRR domains. Collectively, our findings reveal a novel phosphorylation site on the NLRP3 protein that positively regulates inflammasome activation and introduce an effective NLRP3 inhibitor with potential for PD therapy and other NLRP3 inflammasome-driven diseases.

## Materials and methods

### Mice

Male C57BL/6 mice (8 weeks old) and pregnant female mice were sourced from the Laboratory Animal Center of Nanjing Medical University and housed under specific pathogen-free (SPF) conditions. Mice were maintained on a 12-hour light/dark cycle in a temperature-controlled environment with unrestricted access to food and water. Additionally, pregnant female C57BL/6 mice (gestational day 14–15) were used to culture primary neurons. *Nlrp3* knockout (KO) and wild-type (WT) littermate mice were maintained under the same SPF conditions as previously described [12]. The study protocols were approved by the Institutional Animal Care and Research Committee of Nanjing Medical University (IACUC No. 2008067).

### The MPTP/p mouse model

C57BL/6 mice were randomly assigned to receive either MPTP/p (MPTP: 20 mg/kg, s.c., and probenecid: 250 mg/kg, i.p.) or saline twice a week for five weeks. One week after the final injection, mice were euthanized, and samples were collected for subsequent analyses.

### Behavioral tests

A series of behavioral tests were performed to assess motor function. Mice were acclimated to the testing environment for one hour prior to each test.

#### Open field test

Mice were placed in the center of a transparent glass box (50 × 50 × 50 cm), and their movements were recorded for 10 min using an automated video tracking system (Clever Sys Inc.). Locomotor activity was scored based on the distance traveled and speed during the initial 5 min.

#### Pole test

Mice were placed facing upward on a vertical wooden pole (50 cm height, 2 cm diameter). After a day of acclimation, the time taken by each mouse to descend the pole (T-total) was measured over three consecutive trials.

#### Rotarod test

Mice were tested on a rotating rod (3 cm diameter) with five compartments (10 cm width each). The apparatus initiated at 5 rpm and accelerated to 30 rpm over 5 min. The latency to fall was recorded across six trials, with a 30-minute rest between trials.

#### Elevated plus maze (EPM)

The EPM consisted of two open and two closed arms elevated 50 cm above the floor. Mice were allowed to explore the maze for 6 min, and their movements in the open arms and center zone were recorded using Clever Sys Inc. software. The apparatus was cleaned with 75% ethanol between trials.

#### Gait analysis

Gait parameters were evaluated using the WalkAnalysisator 1.0.9 system (Beijing Zhongshi Dichuang Technology Development Co., Ltd.). Mice were trained to walk through a transparent glass walkway, and their footprints were captured by a high-definition camera. Gait metrics such as stride length and movement trajectory were analyzed.

### Virtual screening analysis

The Alphafold 2 database model was applied to predict the potential binding region of the NLRP3 protein (PDB: 6NPY). Virtual docking simulations were performed between the NLRP3 protein and 5,088 natural compounds using PyMOL software. Compounds were ranked based on Gibbs free energy scores.

### Surface plasmon resonance (SPR) analysis

SPR analysis was performed as described previously [22]. Recombinant human NLRP3 protein (CUSBIO, #CSB-EP822275HU3) was immobilized on a CM5 chip (GE, USA), and interactions with different concentrations of compounds were quantified. The equilibrium dissociation constant (KD) was calculated using a 1:1 binding model in the Reichert data evaluation software.

### Primary cell and cell lines cultures

Bone marrow-derived macrophages (BMDMs) were isolated and cultured as described [22]. Primary astrocytes and microglia were isolated from 3-day-old neonatal mice as previously reported [23]. Cells were cultured in DMEM/F12 medium supplemented with 10% fetal bovine serum (FBS) and 100 units/ml penicillin and streptomycin. Primary astrocytes were cultured for 14 days with medium changes every three days, while microglia were cultured for 10 days and harvested by gentle agitation. Primary dopaminergic neurons from the midbrain of pregnant female mice (gestational day 14–16) were cultured in a neurobasal medium supplemented with 10% B27 and antibiotics, as described [23]. SH-SY5Y (CRL-2266) and HEK-293T cells (SCSP-502) were obtained from the American Type Culture Collection and the National Collection of Authenticated Cell Cultures and cultured following the respective protocols.

The schematic model of the coculture system is illustrated in the supplemental Figure S4A. For MCM (microglia conditional medium) and ACM (astrocytic conditional medium) collection, microglia, and astrocytes were incubated in serum-free medium for 1 h to allow the cells to adapt to the culture environment and then exposed to the following stimulation: (i) LPS (100 ng/mL) plus ATP (5 mM, 30 min before) for 6 h. (ii) LPS/ATP stimulation after pretreatment with either Psoralen or NLRP3 inhibitor MCC950 at 10 µM for 1 h. The conditioned medium from activated microglia (MCM) was collected, filtered using 0.22 μm pore filters, and stored at − 80 °C until use. The neurons were plated on PLL-coated wells and cultured in DMEM/F12 medium supplemented with 10% FBS and 1% streptomycin/penicillin for 6 h. The media were changed to neurobasal medium supplemented with 2% B27 and 0.5 mM glutamine and half-changed every 3 days. After 6 days, neurons were treated with the conditioned medium (MCM or ACM: neurobasal = 1:2) for 24 h.

### The NLRP3 inflammasome activation models

BMDMs and glial cells (microglia and astrocytes) were plated in 12-well plates and primed with lipopolysaccharide (LPS, 100ng/mL) for 5.5 h. Cells were subsequently stimulated with ATP (5mM) or nigericin (10µM) for 30 min, after which supernatants and cell lysates were collected for immunoblotting.

For the washout experiment, LPS-primed BMDMs were incubated with Pso for 1 h, and then three washes over 15 min were performed to remove the unbound drug before nigericin stimulation. Then the supernatants were collected for ELISA detection.

### Cell viability and LDH release assays

Cell viability was assessed using the CCK-8 cell proliferation and cytotoxicity assay kit (CA1210, Solarbio, China), and lactate dehydrogenase (LDH) release was measured using the LDH activity detection kit (BC0680, Solarbio, China). All experimental procedures and data analysis were conducted as per the manufacturer’s instructions.

### ELISA assays

Supernatants from BMDMs and glial cells were collected, and the levels of IL-1β (EM001), IL-6 (EM004), and TNF-α (EM008) were quantified using ELISA kits (Excell Bio, Shanghai, China) following the manufacturer’s instructions.

### Western blotting

Cell and tissue lysates were prepared using RIPA buffer (Beyotime, P0013B) supplemented with protease inhibitors. Lysates were centrifuged at 16,000 g for 15 min, and protein concentrations were determined using a BCA assay (Beyotime, P0010). Proteins were resolved on 8–15% SDS-PAGE gels, transferred to PVDF membranes, and blocked with 5% BSA. Membranes were incubated with primary antibodies overnight at 4 °C, followed by secondary antibody incubation for 1 h. Protein bands were visualized using a chemiluminescence imaging system (Bio-Rad). Antibody details are provided in Supplementary Table 2.

### Immunohistochemistry and Immunofluorescence

Mice were perfused with 4% paraformaldehyde, and brains were sectioned at 25 μm thickness. Sections were treated with 3% hydrogen peroxide to quench endogenous peroxidase activity, blocked with 5% BSA, and incubated with primary antibodies overnight. Immunohistochemical staining was performed using a commercial DAB kit (MX Biotechnologies, DAB0031). Immunofluorescence was performed similarly, with sections incubated with Alexa Fluor-conjugated secondary antibodies. Nuclei were counterstained with Hoechst (Sigma, 33342), and sections were imaged using confocal microscopy (FV3000, Olympus). The number of TH^+^ neurons, GFAP^+^ astrocytes, IBA-1^+^ microglia, and Nissl^+^ neurons in the SNpc were calculated using Stereo Investigator (MBF Bioscience, Williston, VT).

### Phos-Tag SDS-PAGE

For analysis of NLRP3 phosphorylation, samples of tissue and cell extracts in SDS-gel sample buffer were subjected to phosphate affinity SDS-PAGE using an acrylamide-pendant phosphate-binding tag (Phos-tag™) with Mn^2+^ as previously described [24]. Standard Tris-Cl buffered stacking (4.5% w/v acrylamide) and separating (6% w/v acrylamide, 50µM Phos-tagged acrylamide, 100µM MnCl_2_) gel recipes were applied. Electrophoresis was performed under constant current conditions (30 mA/gel) until the BPB reached the bottom of the resolving gel. For the transfer of gel-separated proteins to PVDF membranes, gels were pretreated by washing in methanol-free transfer buffer with 5mM EDTA for 10 min twice to remove bivalent cations. Immunoblotting was performed with primary antibody against NLRP3.

### 4D-label free phosphoproteomics

#### Protein extraction and digestion

SDT (4%SDS, 100mM Tris-HCl, 1mM DTT, pH 7.6) buffer was used for sample lysis and protein extraction. The amount of protein was quantified with the BCA Protein Assay Kit (Bio-Rad, USA). Protein digestion by trypsin was performed according to the filter-aided sample preparation (FASP) procedure described by Matthias Mann.

#### Phosphopeptides enrichment

IMAC enrichment method. The enrichment of phosphopeptides was carried out using High-Select™ Fe-NTA Phosphopeptides Enrichment Kit according to the manufacturer’s instructions (Thermo Scientific). After lyophilized, the phosphopeptides peptides were resuspended in 20µL loading buffer (0.1% formic acid).

#### LC-MS/MS analysis

LC-MS/MS analysis was performed on a timsTOF Pro mass spectrometer (Bruker) and was coupled to Nanoelute (Bruker Daltonics) for 60 min. The peptides were loaded on a C18-reversed phase analytical column (homemade, 25 cm long, 75 μm inner diameter, 1.9 μm, C18) in buffer A (0.1% Formic acid) and separated with a linear gradient of buffer B (84% acetonitrile and 0.1% Formic acid) at a flow rate of 300nl/min. The mass spectrometer was operated in positive ion mode. The mass spectrometer collected ion mobility MS spectra over a mass range of m/z 100–1700 and 1/k0 of 0.6 to 1.6 and then performed 10 cycles of PASEF MS/MS with a target intensity of 1.5k and a threshold of 2500. Active exclusion was enabled with a release time of 0.4 min.

#### Identification and quantitation of phosphorylated proteins

The MS raw data for each sample were combined and searched using the MaxQuant software for identification and quantitation analysis.

### Bioinformatic analysis

#### Motif analysis

The motifs were analyzed by MeMe (http://meme-suite.org/index.htm). We extracted the amino acid sequences containing the modified site and six upstream/downstream amino acids from the modified site (13 amino acid sites in total). These sequences were used to predict motifs in this study (parameters: width: 13, occurrences:20, background: species).

#### Subcellular localization

CELLO (http://cello.life.nctu.edu.tw/*)* which is a multi-class SVM classification system, was used to predict protein subcellular localization.

#### Domain annotation

Protein sequences are searched using the InterProScan software to identify protein domain signatures from the InterPro member database Pfam.

#### GO annotation

The protein sequences of the selected differentially expressed proteins were locally searched using the NCBI BLAST + client software (ncbi-blast-2.2.28+-win32.exe) and InterProScan to find homologue sequences, then gene ontology (GO) terms were mapped and sequences were annotated using the software program Blast2GO. The GO annotation results were plotted by R scripts.

#### KEGG annotation

Following annotation steps, the studied proteins were blasted against the online Kyoto Encyclopedia of Genes and Genomes (KEGG) database (http://geneontology.org/) to retrieve their KEGG orthology identifications and were subsequently mapped to pathways in KEGG.

#### Enrichment analysis

Enrichment analysis was applied based on the Fisher’ exact test, considering the whole quantified proteins as background dataset. Benjamini- Hochberg correction for multiple testing was further applied to adjust derived p-values. And only functional categories and pathways with p-values under a threshold of 0.05 were considered as significant.

### Co-IP assay

Cell samples were harvested with NP40 lysis buffer (Beyotime, P0013F) containing protease inhibitors. Equal amounts of protein were then incubated with NLRP3 antibody at 4℃ overnight. Protein A/G PLUS-Agarose (Santa Cruz Biotechnology, SC-2003) was added to incubate with samples for 4 h at room temperature. The immunoprecipitated complexes were washed three times with lysis buffer and denatured by adding loading buffer, followed by boiling for 5 min. Western blotting analysis was used to detect the immunoprecipitated samples.

### Pulldown assay

HEK-293T cells were transfected with plasmids, including Flag-NLRP1, Flag-NLRP3 plasmid, Flag-AIM2, Flag-NLRC4, Flag-NLRP3 (PYD: 1-93aa), Flag-NLRP3 (NACHT: 220-536aa), or Flag-NLRP3 (LRR: 742-991aa). The lysates were incubated with streptavidin beads. The interaction between biotin-labeled compounds and recombinant proteins was analyzed by Western blotting. Details of antibodies and plasmids are provided in Supplemental Tables 2 and Table 3.

### Ultra-performance liquid chromatography-mass spectrum (UPLC–MS)

The concentrations of psoralen in mouse plasma and brain were quantified using liquid chromatography-mass spectrometry with a Thermo TSQ Quantis LC-MS/MS System equipped with an electrospray ionization interface used to generate positive ions [M + H]^+^ for psoralen as previously described [12].

### High-performance liquid chromatography (HPLC)

The mice were sacrificed and the striatum was collected to measure the levels of monoamine transmitters (DA, 5-HIAA, HVA, and 5-HT). The samples (10 µL/mg) were homogenated in the buffer containing 0.1 mol/L perchloric acid, 0.1mM EDTA-2Na, 4 × 10^8^ mol/L DHBA (Sigma, 858781), and centrifuged with 20,000 g for 20 min at 4 °C, then the supernatant was collected for measurement. HPLC detection system and Parameters were set as previously described [23].

### Real-time qPCR

Total RNA was extracted from BMDMs using TRIzol™ reagent (ThermoFisher Scientific, 15596018CN) and dissolved in 20 µL DEPC-treated water. RNA concentration and purity were assessed using a OneDrop™ OD-1000 + Ultra-Micro Spectrophotometer (OneDrop). Complementary DNA (cDNA) was synthesized from 1 µg of total RNA using the HiScript III RT SuperMix for qPCR (Vazyme, R323-01) following the manufacturer’s protocol. qPCR amplification was conducted with ChamQ Universal SYBR qPCR Master Mix (Vazyme, Q341-02) under the following conditions: initial denaturation at 95 °C for 30 s, followed by 40 cycles of denaturation at 95 °C for 10 s, and annealing/extension at 60 °C for 30 s. Melting curve analysis was performed with the following steps: 95 °C for 10 s, 60 °C for 30 s, and 95 °C for 15 s. Relative gene expression was calculated using the 2^^−ΔΔCt^ method, with normalization to Gapdh as the reference gene. Details of primers and small interfering RNA sequences are provided in Supplemental Table 4.

### Longitudinal functioning experiment

This Longitudinal functioning experiment (last for 8 weeks) was divided into two stages. Chronic MPTP/p treatment (5 weeks) is Stage 1, and administration for another 3 consecutive weeks is Stage 2. After Stage 1 chronic MPTP/p treatment, behavioral test 1 (green) was performed to assess the successful PD modeling and efficacy of Pso and MCC950. Then mice were administrated for another 3 consecutive weeks (without MPTP/p) as Stage 2 to observe longitudinal function in the behavioral test 2 (orange). The Schematic diagram of the longitudinal experimental procedure is illustrated in Fig. S10A.

### Stereotaxic surgery

AAV vectors expressing NLRP3 or its mutant (S658A) were injected bilaterally (300nl/side) into the midbrain of mice using a stereotaxic apparatus (RWD Life Science). The coordinates are AP: -3.0 mm, ML: ±1.3 mm, and DV: -4.5 mm. After surgery, the mice were maintained for 3 weeks to allow for viral expression.

### Statistical analysis

Data are expressed as mean ± S.E.M. Statistical analyses were conducted using GraphPad Prism 9. Student’s t-test, one-way ANOVA, or two-way ANOVA with Tukey’s multiple comparisons test were used. Statistical significance was set at *p* < 0.05. All in vitro experiments included at least three biological and two technical replicates. Further details are provided in figure legends.

## Results

### Virtual screening and validation of natural product inhibitors targeting NLRP3 inflammasome in BMDMs

Numerous studies have established a strong link between NLRP3 inflammasome activation and the progression of PD [25]. In this study, we observed a loss of DA neurons in the SNc of MPTP/p-treated mice, which was associated with heightened reactivity of microglia and astrocytes (Fig. S1A-D). Notably, the presence of NLRP3-positive microglia in MPTP/p-treated mice was significantly higher (80.43%) compared to saline-treated controls (37.76%) (Fig. S1E-F). Similarly, the proportion of NLRP3-positive astrocytes increased from 28.61% in saline-treated mice to 67.56% in MPTP/p-treated mice (Fig. S1G-H). These findings suggest that glial NLRP3 expression contributes to the degeneration of DA neurons. To further validate this, we analyzed PD pathology in MPTP/p-treated WT and *Nlrp3* KO mice. Behavioral assessments showed that *Nlrp3* KO mice exhibited improved motor performance compared to WT mice. In the open field test, *Nlrp3* KO mice displayed increased movement distance following MPTP/p treatment (Fig. S2A-B). In the rotarod test, the latency to fall was significantly extended in *Nlrp3* KO mice under MPTP/p treatment compared to WT mice (Fig. S2C). Similarly, *Nlrp3* KO mice demonstrated reduced time to complete the pole test compared to WT mice after MPTP/p treatment (Fig. S2D). In the elevated plus maze test, *Nlrp3* KO mice exhibited longer stride lengths than WT mice in the MPTP/p treatment context (Fig. S2E-F). Although no significant differences were observed between saline- and MPTP/p-treated mice, *Nlrp3* KO mice exhibited greater locomotion in both the center and open-arm regions (Fig. S2G-H). Additionally, immunofluorescence staining of midbrain slices revealed that *Nlrp3* KO attenuated the loss of DA neurons and reduced glial reactivity in MPTP/p-treated mice (Fig. S2I). These findings support the notion that NLRP3 expression in glial cells contributes to DA neuronal loss and PD progression.

**Fig. 1:**
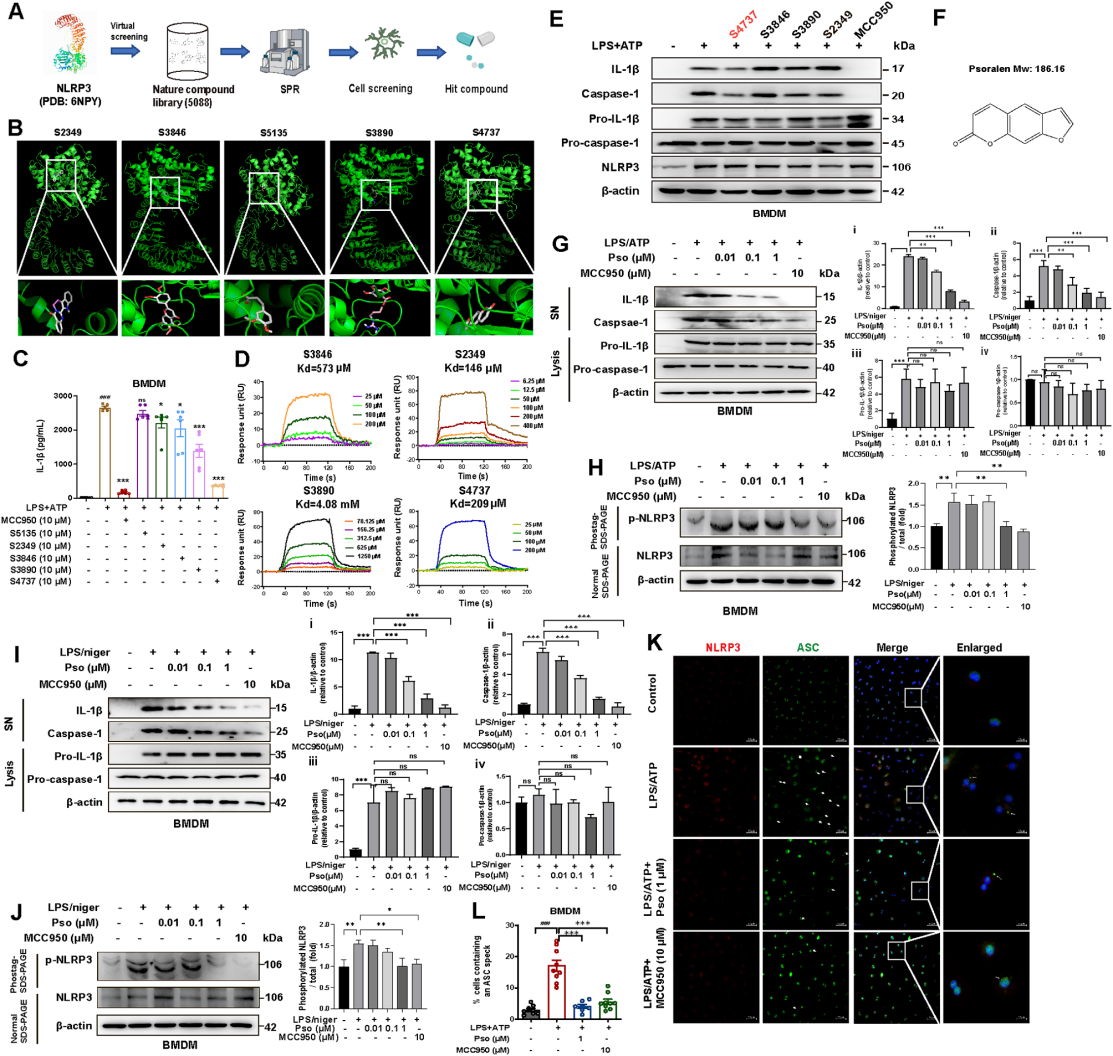
Virtual screening and validation of potential NLRP3 inflammasome inhibitors in BMDMs. **(A)** The schematic model of screening NLRP3 inflammasome inhibitors from a nature compound library. **(B)** Five potential NLRP3 inflammasome inhibitors from 5,088 natural products were selected based on docking scores. **(C)** LPS (100 ng/mL) primed-BMDMs were treated with candidates and then stimulated with ATP (5 mmol/L). The levels of IL-1β in the supernatant were measured by ELISA, *n* = 6. MCC950 as a positive drug for inhibiting NLRP3 inflammasome activation. **(D)** The binding affinity between candidate compounds and purified human NLRP3 protein was examined by SPR assay. **(E)** Levels of IL-1β and caspase-1 in SN (Supernatant) and levels of pro-IL-1β/pro-caspase-1 in cell lysates (Lysates) were analyzed by Western blotting to examine the effects of candidate drugs on NLRP3 inflammasome activation. **(F)** The chemical structure of S4737 (Psoralen). Levels of IL-1β and caspase-1 in SN and levels of pro-IL-1β/pro-caspase-1 in Lysates were analyzed by immunoblotting in BMDMs pretreated with different concentrations of Psoralen (0.01, 0.1 and 1 µM) followed by stimulation with LPS/ATP **(G)** or LPS/Nigericin **(I)**. Phos-tag SDS-PAGE and quantification of the phosphorylation levels of NLRP3 in BMDMs pretreated with Psoralen (0.01, 0.1 and 1 µM) followed by stimulation with LPS/ATP **(H)** or LPS/Nigericin **(J)**. **(K)** Immunofluorescence staining for NLRP3 (red) and ASC (green) in the LPS/ATP treated BMDMs. DAPI stains the nucleus (blue). The scale bar represents 50 μm. Enlarge vision: 10 μm. **(L)** The percentage of cells containing an ASC speck was quantified. 100 BMDMs per group were analyzed. Data were analyzed by one-way ANOVA, followed by Tukey post-tests. ^*^P < 0.05, ^**^P < 0.01, and ^***^P < 0.001. ns: no significance

Given the therapeutic potential of inhibiting NLRP3 inflammasome activation in PD [25], we conducted a virtual screening of a natural product library consisting of 5,088 compounds (Fig. 1A). Based on docking scores, five candidate compounds (S2349, S3846, S5135, S3890, and S4737) were selected for further evaluation (Fig. 1B, Supplementary Table 1). To assess their inhibitory effects on NLRP3 inflammasome activation, we measured IL-1β release from BMDMs treated with LPS + ATP, using MCC950 as a positive control [26]. All compounds, except S5135, significantly reduced IL-1β release at a concentration of 10 µM, with S4737 emerging as the most effective (Fig. 1C). Surface plasmon resonance (SPR) assays were conducted to determine the binding affinities of the four active compounds to NLRP3. The equilibrium dissociation constants (Kds) were calculated as follows: S3846 (573 µM), S2349 (146 µM), S3890 (4.08 mM), and S4737 (209 µM) (Fig. 1D). Subsequent western blotting analysis of IL-1β and caspase-1 expression in LPS + ATP-stimulated BMDMs identified S4737 as the most effective inhibitor of NLRP3 inflammasome activation, and it was selected for further study (Fig. 1E). To explore the concentration-dependent effects of S4737, also known as Psoralen, concentrations ranging from 0.01 to 1 µM were tested on NLRP3 inflammasome activation in BMDMs (Fig. 1F). Psoralen (Pso) treatment did not affect cell viability and LDH release but significantly reduced IL-1β and caspase-1 levels in the supernatant of LPS + ATP-stimulated BMDMs in a concentration-dependent manner (Fig. S3A, D and Fig. 1G). Interestingly, Psoralen selectively inhibited the phosphorylation of NLRP3, while total NLRP3 levels remained unchanged (Fig. 1H). To validate the inhibitory effect of Psoralen, we employed a second model of NLRP3 inflammasome activation using LPS + Nigericin in BMDMs [27]. Consistent with the previous findings, Psoralen reduced NLRP3 inflammasome activation and phospho-NLRP3 expression in a concentration-dependent manner (Fig. 1I-J). Immunofluorescence staining for NLRP3 and ASC showed a decrease in ASC speck-positive BMDMs upon Psoralen treatment under LPS + ATP condition (Fig. 1K-L). In summary, our results confirm that Psoralen effectively inhibits NLRP3 inflammasome activation and may hold potential as a therapeutic agent for PD.

### Psoralen inhibits NLRP3 phosphorylation and NLRP3 inflammasome activation in microglia and astrocytes

**Fig. 2:**
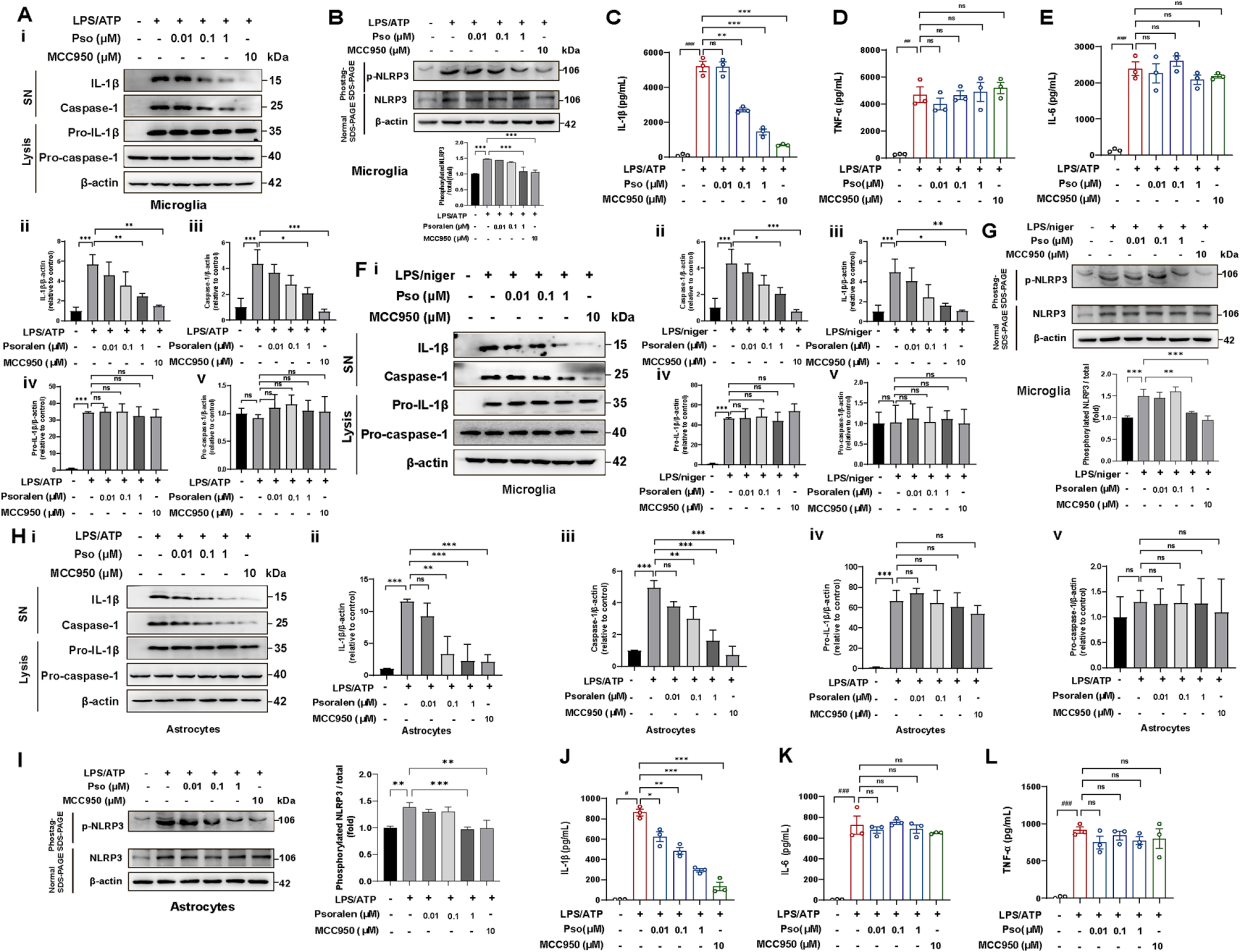
Psoralen prevents NLRP3 inflammasome activation in microglia and astrocytes. **(A)** LPS (100 ng/mL) primed microglia were treated with different concentrations (0.01, 0.1, and 1 µM) of Pso and then stimulated with ATP (5 mmol/L). The levels of IL-1β and caspase-1 in SN and levels of pro-IL-1β/pro-caspase-1 in Lysates were analyzed by immunoblotting. Quantification of relative expression of IL-1β (i), caspase-1(ii), pro-IL-1β (iii), and pro-caspase-1(iv) in microglia. All experiments were performed with three biological replicates. **(B)** Phos-tag SDS-PAGE and quantification of NLRP3 phosphorylation levels in microglia treated with LPS/ATP. Measurement of IL-1β **(C)**, TNF-α **(D)**, and IL-6 **(E)** levels in the supernatant of microglia, *n* = 3. **(F)** LPS (100 ng/mL) primed microglia were treated with different concentrations (0.01, 0.1, and 1 µM) of Pso and then stimulated with nigericin (5µmol/L). Quantification of relative expression of IL-1β (i), caspase-1(ii), pro-IL-1β (iii), and pro-caspase-1(iv) in microglia stimulated with LPS/nigericin. **(G)** Phos-tag SDS-PAGE and quantification on NLRP3 phosphorylation treated with LPS/nigericin in microglia. **(H)** LPS (100 ng/mL) primed astrocytes were treated with different concentrations (0.01, 0.1, and 1 µM) of Pso and then stimulated with ATP (5 mmol/L). Quantification of relative expression of IL-1β (i), caspase-1(ii), pro-IL-1β (iii), and pro-caspase-1(iv) in astrocytes stimulated with LPS/ATP. **(I)** Phos-tag SDS-PAGE and quantification on NLRP3 phosphorylation treated with LPS/ATP in astrocytes. Measurement of IL-1β **(J)**, IL-6 **(K)**, and TNF-α **(L)** levels in astrocytes, *n* = 3. Data were analyzed by one-way ANOVA, followed by Tukey post-tests. ^*^P < 0.05, ^**^P < 0.01, and ^***^P < 0.001. ns: no significance

We further investigated the inhibitory effects of Psoralen on NLRP3 inflammasome activation in microglia and astrocytes, two key glial cell types that exacerbate neuroinflammation and contribute to PD progression. In microglia, Psoralen (Pso) treatment at concentrations ranging from 0.01 to 100 µM had no significant effect on cell viability and LDH release (Fig. S3B, E). When microglia were stimulated with LPS + ATP, NLRP3 inflammasome activation was indicated by increased IL-1β and caspase-1 levels in the supernatant. However, Pso treatment (0.01-1µM) significantly and concentration-dependently suppressed NLRP3 inflammasome activation (Fig. 2A_i − v_). Additionally, Pso inhibited the phosphorylation of NLRP3 in LPS + ATP-stimulated microglia, consistent with the results observed in BMDMs (Fig. 2B). Notably, Pso treatment selectively reduced IL-1β release without affecting TNF-α or IL-6 levels, suggesting a specific inhibitory effect on NLRP3 inflammasome activation in microglia (Fig. 2C-E). We further validated these findings using an alternative model of NLRP3 inflammasome activation in microglia, stimulated with LPS + Nigericin. Pso treatment (0.01-1µM) again demonstrated a concentration-dependent inhibition of NLRP3 inflammasome activation and reduced phospho-NLRP3 expression (Fig. 2F_i − v_-2G). In astrocytes, Pso treatment across a similar concentration range (0.01–100 µM) did not affect cell viability and LDH release, although a higher concentration (300 µM) reduced cell viability (Fig. S3C, F). LPS + ATP stimulation led to NLRP3 inflammasome activation and increased phospho-NLRP3 expression in astrocytes, both of which were effectively blocked by Pso treatment at 1µM (Fig. 2H_i − v_-2I). Similarly, Pso specifically inhibited NLRP3 inflammasome activation in LPS + ATP-stimulated astrocytes, as evidenced by reduction in the release of IL-1β but not TNF-α or IL-6 (Fig. 2J-L). Collectively, these results demonstrate that Psoralen is a potent inhibitor of NLRP3 inflammasome activation in both microglia and astrocytes, which may help mitigate neuroinflammation and slow PD progression.

### Psoralen treatment ameliorates PD-like motor symptoms and DA neuronal death through Inhibition of NLRP3 inflammasome activation

**Fig. 3:**
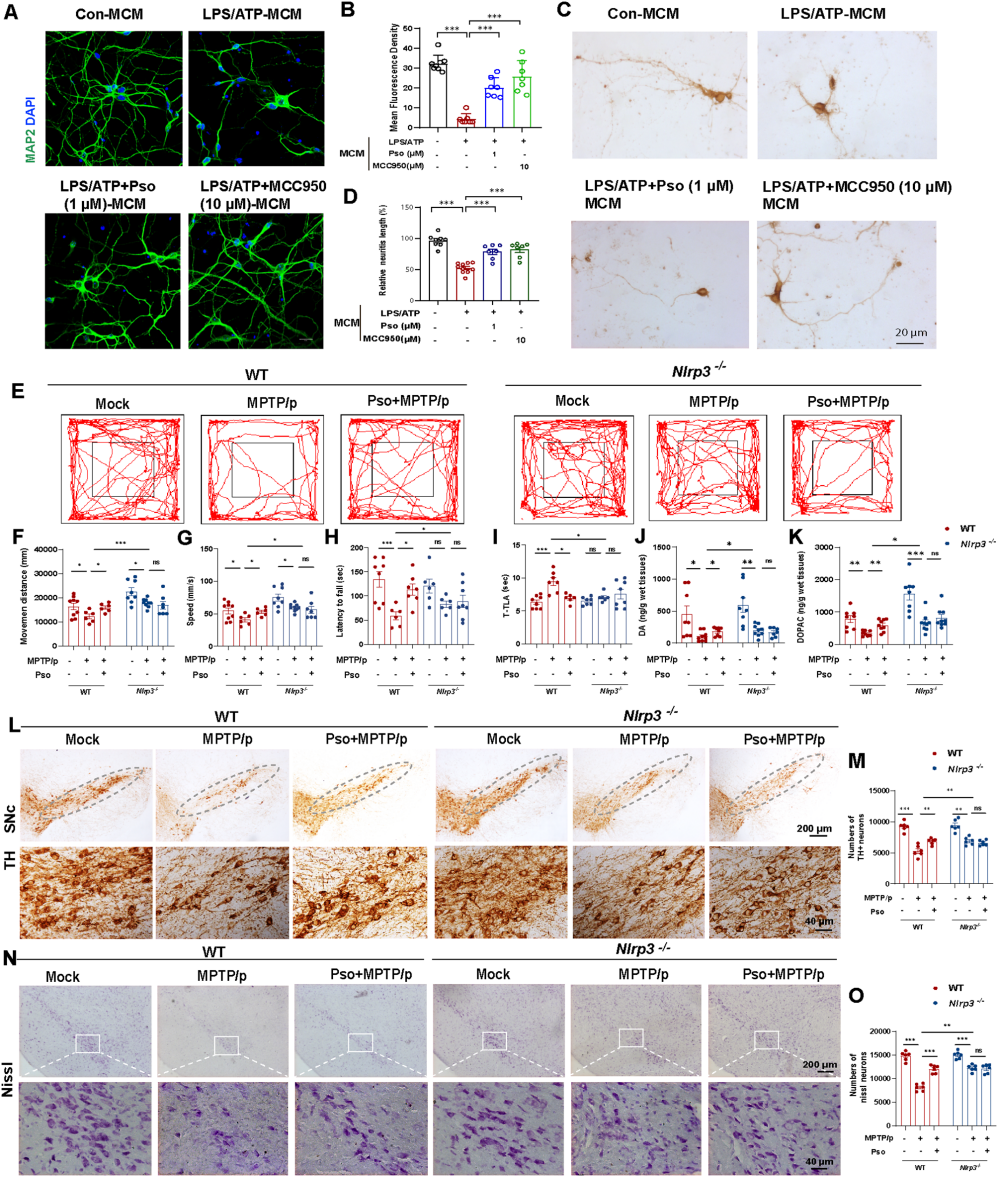
Psoralen ameliorates PD-like motor symptoms and DA neuronal death in the presence of functional NLRP3. Primary neurons were incubated with conditioned medium (MCM) from microglia treated with LPS/ATP and Pso (1 µM). Representative immunostaining **(A)** and quantification **(B)** of MAP2 intensity (green). The scale bar represents 20 μm. **(C-D)** Effects of MCM on the morphology of DA neurons observed by immunohistochemical staining of TH. The scale bar represents 20 μm. **(E-G)** Travel path, movement distance and speed were recorded in the open field test, *n* = 6–10. **(H)** Latency to fall was recorded in the rotarod test, *n* = 6–8. **(I)** The time taken to descend a pole (T-TLA) was recorded in the pole test, *n* = 6–8. **(J-K)** The levels of dopamine and DOPAC in the striatum homogenate were detected by HPLC, *n* = 8–10. **(L-M)** Immunohistochemical staining and counting of TH^+^ neurons in the SNc. The scale bar represents 200 μm. Enlarge vision: 40 μm. **(N-O)** Staining and counting of Nissl^+^ neurons in the SNc. The scale bar represents 200 μm. Enlarge vision: 40 μm. Data were analyzed by one-way ANOVA, followed by Tukey post-tests. ^*^P < 0.05, ^**^P < 0.01, and ^***^P < 0.001. ns: no significance

To evaluate the therapeutic potential of Psoralen in PD, we employed an indirect co-culture system of microglia/astrocytes and neurons (Fig. S4A). Conditioned medium (MCM) from LPS + ATP-treated microglia significantly damaged primary neurons, but this damage was markedly reduced by Pso-containing MCM, as shown by MAP2 staining (Fig. 3A-B). Notably, this neuroprotective effect was also observed in DA neurons, as demonstrated by immunohistochemical and immunofluorescence staining for TH (Fig. 3C-D and Fig. S4B-C). Similarly, the conditioned medium from LPS + ATP-treated astrocytes (ACM) caused neuronal damage, which was also mitigated by Pso-containing ACM (Fig. S4D-G). Interestingly, Pso did not affect cell viability or LDH release in SH-SY5Y cells under both basal and MPP^+^ conditions (Fig. S5A-D), suggesting that its neuroprotective effects are mediated through the inhibition of glial NLRP3 inflammasome activation, rather than direct effects on neurons. We next assessed the effects of Pso in MPTP/p-treated WT and *Nlrp3* KO mice (Fig. S6A). The ability of Pso to cross the blood-brain barrier was confirmed using UPLC-MS/MS. Upon administration, Pso (20 mg/kg, i.g.) successfully penetrated the blood-brain barrier, with increasing concentrations detected in plasma and brain over time (Fig. S6B-C). Behavioral tests revealed that Pso treatment improved motor performance in MPTP/p-treated WT mice but not in *Nlrp*3 KO mice. In the open field test, Pso increased both movement distance and speed in WT but not *Nlrp*3 KO mice (Fig. 3E-G). Meanwhile, Pso treatment extended the latency to fall in WT but not *Nlrp3* KO mice in the rotarod test (Fig. 3H). Likewise, in the pole test, Pso improved performance in WT but had no effect in *Nlrp3* KO mice (Fig. 3I). Analysis of neurotransmitter levels in the striatum by HPLC revealed that Pso treatment significantly increased the levels of dopamine and its metabolites (DOPAC and HVA) in MPTP/p-treated WT mice, an effect not observed in *Nlrp3* KO mice (Fig. 3J-K, Fig. S7A). However, Pso had no effect on 5-HT or 5-HIAA levels (Fig. S7B-C). Moreover, Pso treatment reduced the loss of DA neurons in the nigrostriatal pathway, as demonstrated by immunohistochemical staining for TH in the SNc and striatum (Fig. 3L-M, Fig. S7D), a finding further supported by Nissl staining of the SNc region (Fig. 3N-O). Collectively, these results suggest that Psoralen confers neuroprotection in the presence of functional NLRP3.

**Fig. 4:**
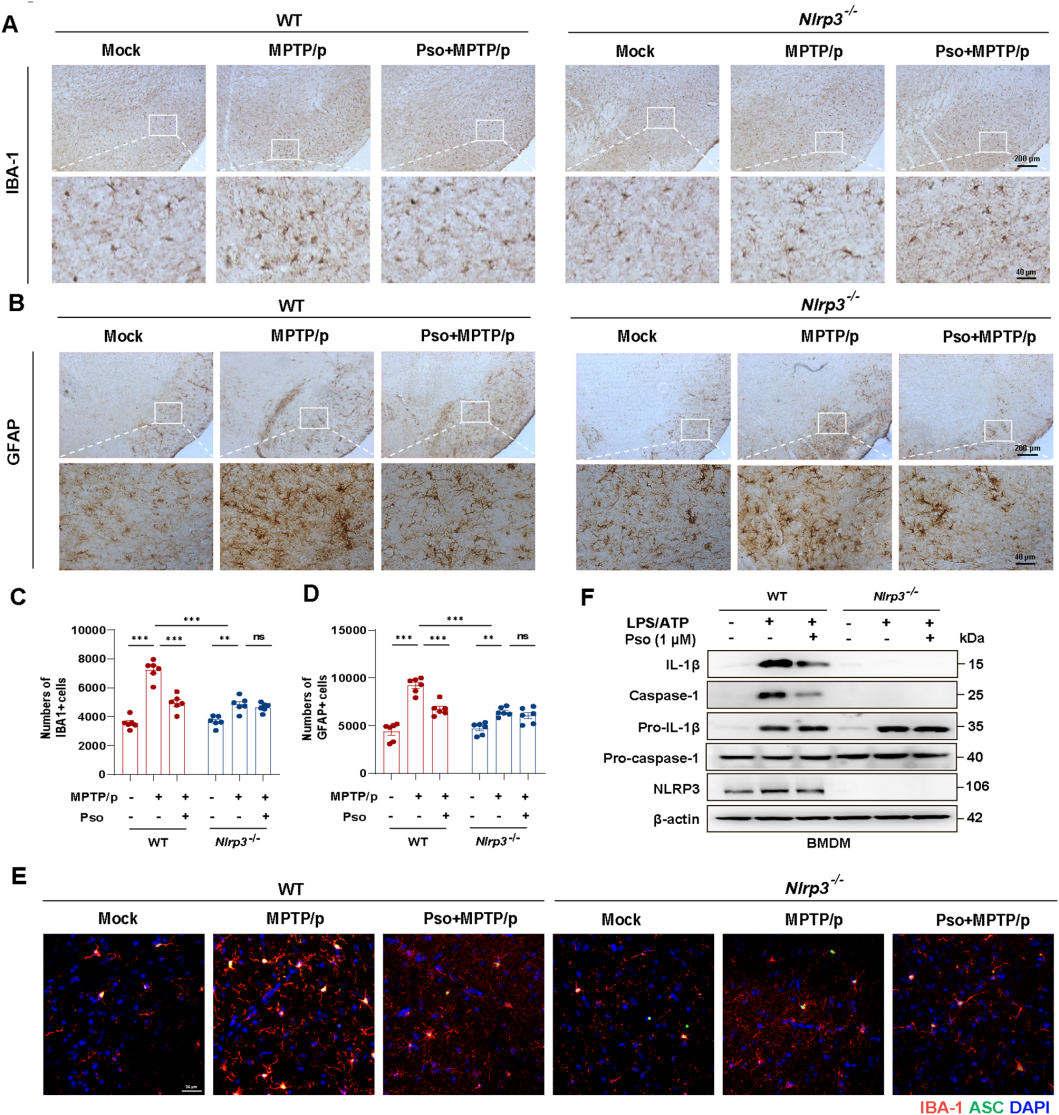
Psoralen prevents glial NLRP3 inflammasome activation in the MPTP/p mouse model. Immunohistochemical staining and counting of IBA-1^+^ cells (**A** and **C**) and GFAP^+^ cells (**B** and **D**). The scale bar represents 200 μm. Enlarge vision: 40 μm. **(E)** Immunofluorescence staining for ASC (green) and microglia marker IBA1 (red) in the SNc of MPTP/p-treated WT and *Nlrp3*^*−/−*^ mice. DAPI stains the nucleus (blue). The scale bar represents 100 μm. **(F)** Effects of Psoralen (1µM) on NLRP3 inflammasome activation in the LPS/ATP primed-BMDMs from WT and *Nlrp3* KO mice. Data were analyzed by one-way ANOVA, followed by Tukey post-tests. ^*^P < 0.05, ^**^P < 0.01, and ^***^P < 0.001. ns: no significance

In MPTP/p-treated WT mice, Pso reduced the number of IBA-1 positive microglia and GFAP positive astrocytes in the SNc, indicating suppression of glial reactivity (Fig. 4A-D). However, this effect was not observed in *Nlrp3* KO mice, which were resistant to MPTP/p-induced glial reactivity, further underscoring the essential role of NLRP3 in mediating the effects of Pso (Fig. 4A-D). Additionally, immunofluorescence staining revealed that Pso inhibited the MPTP/p-induced assembly of NLRP3 inflammasome, as indicated by reduced ASC speck formation, an effect absent in *Nlrp3* KO mice (Fig. 4E). Consistent with these findings, Pso treatment significantly prevented LPS + ATP-induced NLRP3 inflammasome activation in BMDMs from WT but not *Nlrp3* KO mice (Fig. 4F). In conclusion, these findings highlight the therapeutic potential of Psoralen in PD by inhibiting glial NLRP3 inflammasome activation.

To investigate the longitudinal functioning of psoralen, we designed the additional experiment (last for 8 weeks) illustrated in Fig. S10A. At the endpoint of Stage 1 (green arrow), behavioral tests showed that Pso (20 mg/kg) treatment improved motor performance in MPTP/p-treated mice. Pso treatment increased movement distance in the OFT (Fig. S10B-C), extended the latency to fall in the rotarod test (Fig. S10D), and improved T-TLA in the pole test (Fig. S10E). Likewise, the MCC950 (10 mg/kg) treatment also significantly improved the motor dysfunction of MPTP/p mice. After another 3 consecutive weeks of administration, at the endpoint of Stage 2 (orange arrow), behavioral tests showed that Pso (20 mg/kg) treatment can still improve motor performance (Fig. S10F-I). As shown in Fig. S10K, Pso alleviates the loss of the dopaminergic neurons, the number of IBA-1-positive microglia, and GFAP-positive astrocytes in the midbrain of MPTP/p-treated mice. These results demonstrated that Pso (20 mg/kg) exerts a neuroprotective effect even at long-term administration. In contrast to Pso, although immunohistochemical staining results showed that MCC950 (10 mg/kg) ameliorated the loss of dopaminergic neurons, the improvement of movement activity after long-term administration was significantly lower than that in the Pso (20 mg/kg) treatment group (Fig. S10F-I). We speculated the therapeutic effects of MCC950 may be abolished due to the long-term hepatotoxicity of MCC950, which is consistent with the previous report^1^. Furthermore, from the body weight curve of mice, mild reductions in weight with prolonged treatment in the MCC950 group were observed compared with that in the Pso group (Fig. S10J). H&E staining demonstrated that MCC950 (10 mg/kg, 8-week) treatment performed an aggravated liver injury in the MPTP/p group (Fig. S10K). Moreover, pyroptosis-related proteins in the striatum were detected by immunoblotting. Both Pso and MCC950 treatment inhibited the activation of the NLRP3 inflammasome, subsequently reducing the expression of the N-terminal pore-forming GSDMD fragment (GSDMD-NT) (Fig. S10L). Taken together, Psoralen treatment (20 mg/kg) performed more sustainable neuroprotective effects even with long-term administration. It has better effects on ameliorating PD-like motor symptoms and DA neuronal death than MCC950. More importantly, Pso exhibits the advantage of higher safety and less hepatotoxicity.

### Psoralen binds to the NACHT and LRR domains of NLRP3

**Fig. 5:**
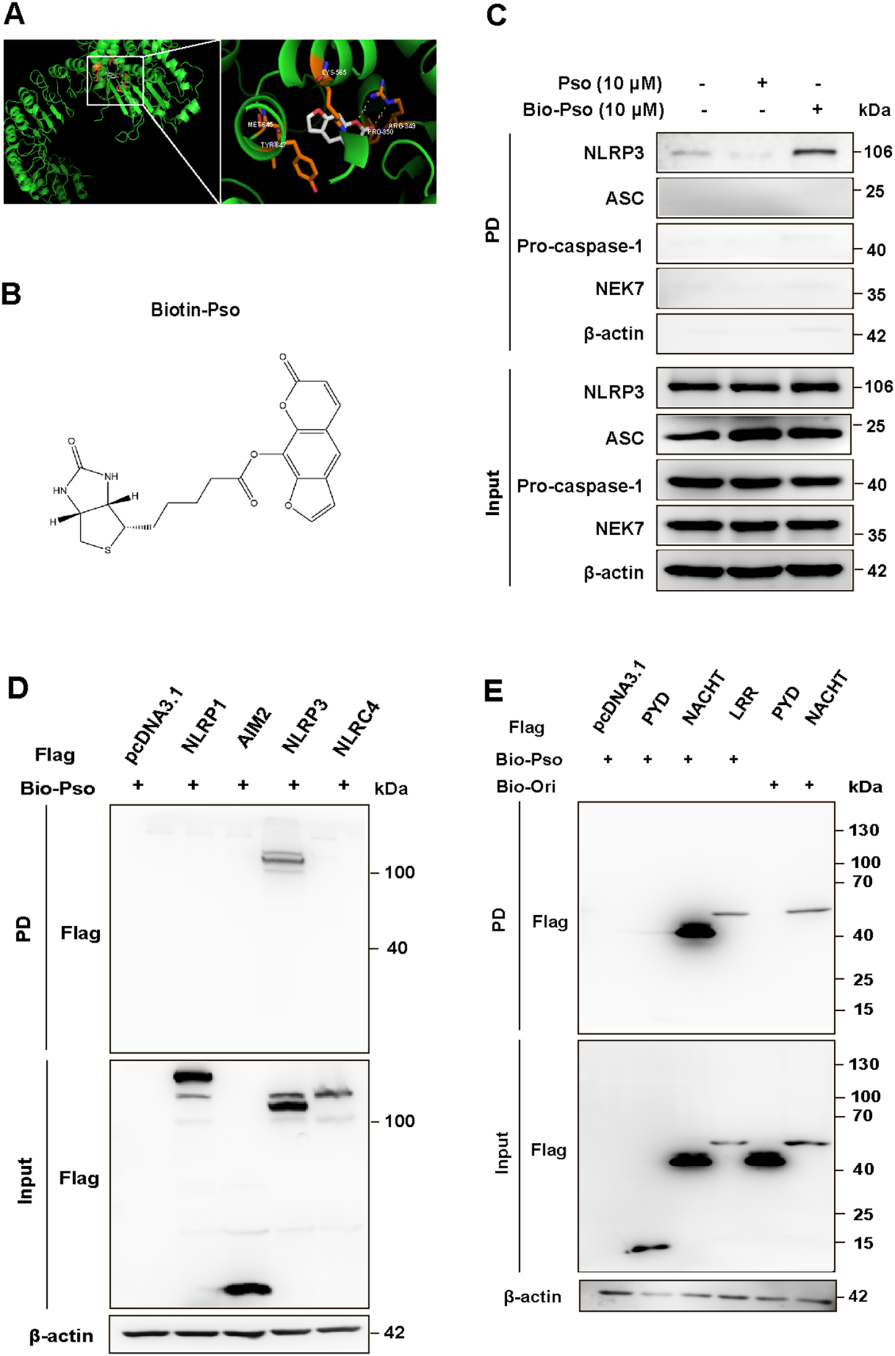
Biotinylated-Psoralen directly binds to the NACHT and LRR domains of NLRP3 protein. **(A)** Docking complex of human NLRP3 protein with Psoralen (Pso). **(B)** The chemical structure of Biotinylated-Psoralen. **(C)** Cell lysates of LPS-primed BMDMs were incubated with Pso or Bio-Pso for 2 h, which were then pulled down using streptavidin beads. The NLRP3 inflammasome components in the pull-down (PD) and total (Input) fractions were examined by Western blotting. Flag-tagged NLRP1, AIM2, NLRP3 or NLRC4 **(D)**, NLRP3-LRR, NLPR3-NACHT or NLRP3-PYD **(E)** was expressed in HEK-293T cells. The HEK-293T cell lysates were incubated with Bio-Pso (10 µM) and then were pulled down using streptavidin beads for Western blotting assay. Bio-Oridonin (1 µM) as a positive drug targeting the NACHT domain of NLRP3 protein

We further elucidate the mechanism underlying Psoralen-mediated inhibition of NLRP3 inflammasome. Molecular docking analysis revealed a direct interaction between Pso and the NLRP3 protein (Fig. 5A). To experimentally validate this interaction, we synthesized biotinylated Psoralen (Bio-Pso) and confirmed its structure through LC-MS and proton nuclear magnetic resonance (HNMR) spectroscopy (Fig. 5B and Fig. S8A-B). BMDMs were treated with either Pso or Bio-Pso for 2 h, followed by LPS + ATP stimulation. Streptavidin beads were then employed to pull down Bio-Pso-bound proteins from cell lysates. The pull-down fractions (PD) contained NLRP3, but no other components of the inflammasome complex, confirming that Pso directly binds to NLRP3 (Fig. 5C). To investigate the specificity of Pso’s binding, we expressed several inflammasome sensors, including NLRP1, AIM2, NLRP3, and NLRC4, in HEK-293T cells and treated them with Bio-Pso. Importantly, only NLRP3 was present in the pull-down fractions, with no binding detected for NLRP1, AIM2, or NLRC4, indicating that Pso specifically binds to NLRP3 (Fig. 5D). Next, we sought to identify the specific domains of NLRP3 to which Pso binds. We cloned and expressed individual domains of NLRP3, including PYD, NACHT, and LRR in HEK-293T cells (Fig. S8C). Biotinylated oridonin (Bio-Ori), known to bind the NACHT domain of NLRP3, was used as a positive control [28]. Streptavidin pull-down assays showed that Bio-Pso specifically bound to the NACHT and LRR domains, but not the PYD domain (Fig. 5E). Besides, to detect whether the binding is reversible (washout experiment), LPS-primed BMDMs were incubated with Pso for 1 h and then performed three washes over 15 min to remove unbound drug before nigericin stimulation. The results showed that Pso still inhibited nigericin-induced IL-1β production after the washout, suggesting Pso is an irreversible inhibitor of NLRP3 inflammasome (Fig. S11). Thus, we have identified that Psoralen binds to both the NACHT and LRR domains of NLRP3, providing mechanistic insight into its specific inhibition of NLRP3 inflammasome activation.

### Psoralen impedes NLRP3 inflammasome activation by blocking NLRP3 phosphorylation at Serine 658

**Fig. 6:**
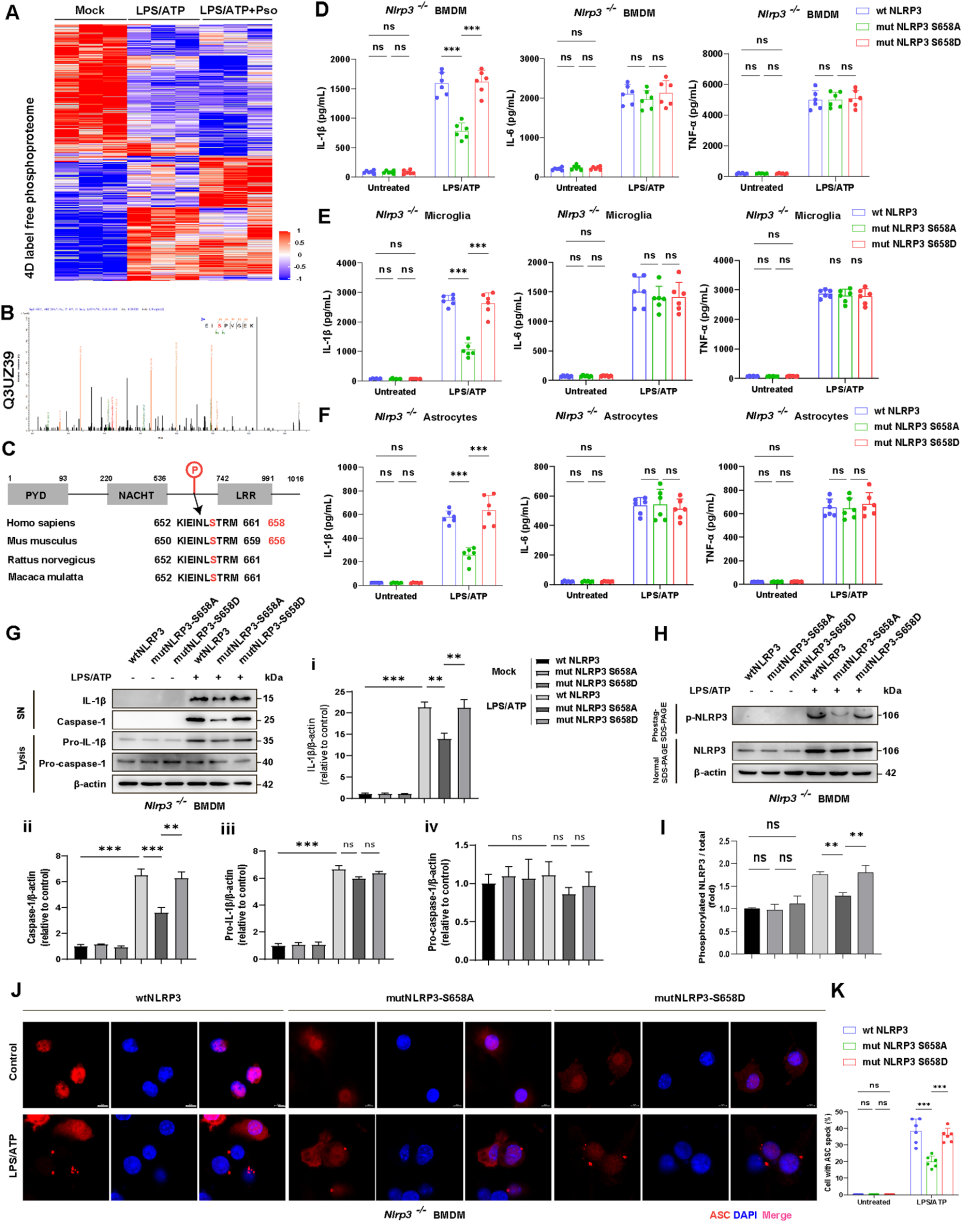
S658 phosphorylation is required for NLRP3 inflammasome activation. **(A)** Heatmap demonstrated the differential phosphorylation peptides (fold change > 1) from 4D label-free quantitative phosphorylation proteomics. **(B)** Representative phosphorylation peptide Q3UZ39, annotated to NLRP3 protein. **(C)** The phosphorylation sites blocked by Psoralen across different species. BMDMs isolated from *Nlrp3* KO mice were transfected with wtNLRP3, *mut*NLRP3-S658A plasmids, or *mut*NLRP3-S658D plasmids, then ELISA detected the production of IL-1β, IL-6, and TNF-α in the supernatant of BMDMs **(D)**, Microglia **(E)**, and Astrocytes **(F)**. *n* = 6. **(G)** IL-1β and caspase-1 from SN and pro-IL-1β/pro-caspase-1 from Lysates were analyzed by immunoblotting. **(H-I)** Phos-tag SDS-PAGE and quantification of NLRP3 phosphorylation levels in *Nlrp3* KO BMDMs transfected with wtNLRP3, *mut*NLRP3-S658A plasmids, or *mut*NLRP3-S658D plasmids. **(J-K)** Immunofluorescence staining and quantification of ASC (red) in *Nlrp3* KO BMDMs transfected with wtNLRP3, *mut*NLRP3-S658A plasmids, or *mut*NLRP3-S658D plasmids. DAPI stains the nucleus (blue). The scale bar represents 5 μm. Data were analyzed by two-way ANOVA, followed by Tukey post-tests. ^*^P < 0.05, ^**^P < 0.01, and ^***^P < 0.001. ns: no significance

NLRP3 phosphorylation is essential for inflammasome activation, as previously demonstrated [20, 21]. To investigate Psoralen’s mechanism of action, we conducted 4D label-free quantitative phosphorylation proteomics to screen for phosphorylation sites on NLRP3 (Fig. S9A). This analysis identified 10,202 phospho-sites, 8,865 phospho-peptides, and 3,672 phospho-proteins across mock-treated, LPS + ATP-stimulated, and LPS + ATP + Pso-treated conditions (Fig. 6A and Fig. S9B). Bioinformatic analyses were performed to explore the cellular location, molecular function, and signaling pathways of differentially expressed proteins (Fig. S9C-F). Notably, NLRP3 was found to be phosphorylated at Serine 658 (Human S658, corresponding to mouse S656) in LPS + ATP-stimulated astrocytes, and this phosphorylation was blocked by Pso treatment (Fig. 6B-C and Fig. S9G). To explore the functional role of S658 phosphorylation in NLRP3 inflammasome activation, we created NLRP3 mutants: a non-phosphorylatable S658A mutant (S658A) and a phospho-mimetic S658D mutant (S658D). These mutant constructs, along with WT-NLRP3, were transfected into *Nlrp3* KO BMDMs, Microglia, and Astrocytes. ELISA assays revealed that both WT-NLRP3 and S658D mutant transfection induced significant IL-1β release in response to LPS + ATP stimulation, whereas transfection with S658A mutant dramatically suppressed IL-1β release in BMDM (Fig. 6D), Microglia (Fig. 6E), and Astrocytes (Fig. 6F). Interestingly, neither the S658A nor S658D mutations affected the release of IL-6 or TNF-α. Western blotting analysis confirmed that the S658A mutant reduced LPS + ATP-induced expression of IL-1β, caspase-1, and phospho-NLRP3 (Fig. 6G-I). Furthermore, immunofluorescence staining showed that the S658A mutant diminished the number of BMDMs containing ASC specks, a marker of inflammasome assembly (Fig. 6J-K). These results indicate that phosphorylation at S658 is crucial for NLRP3 inflammasome assembly and activation.

**Fig. 7:**
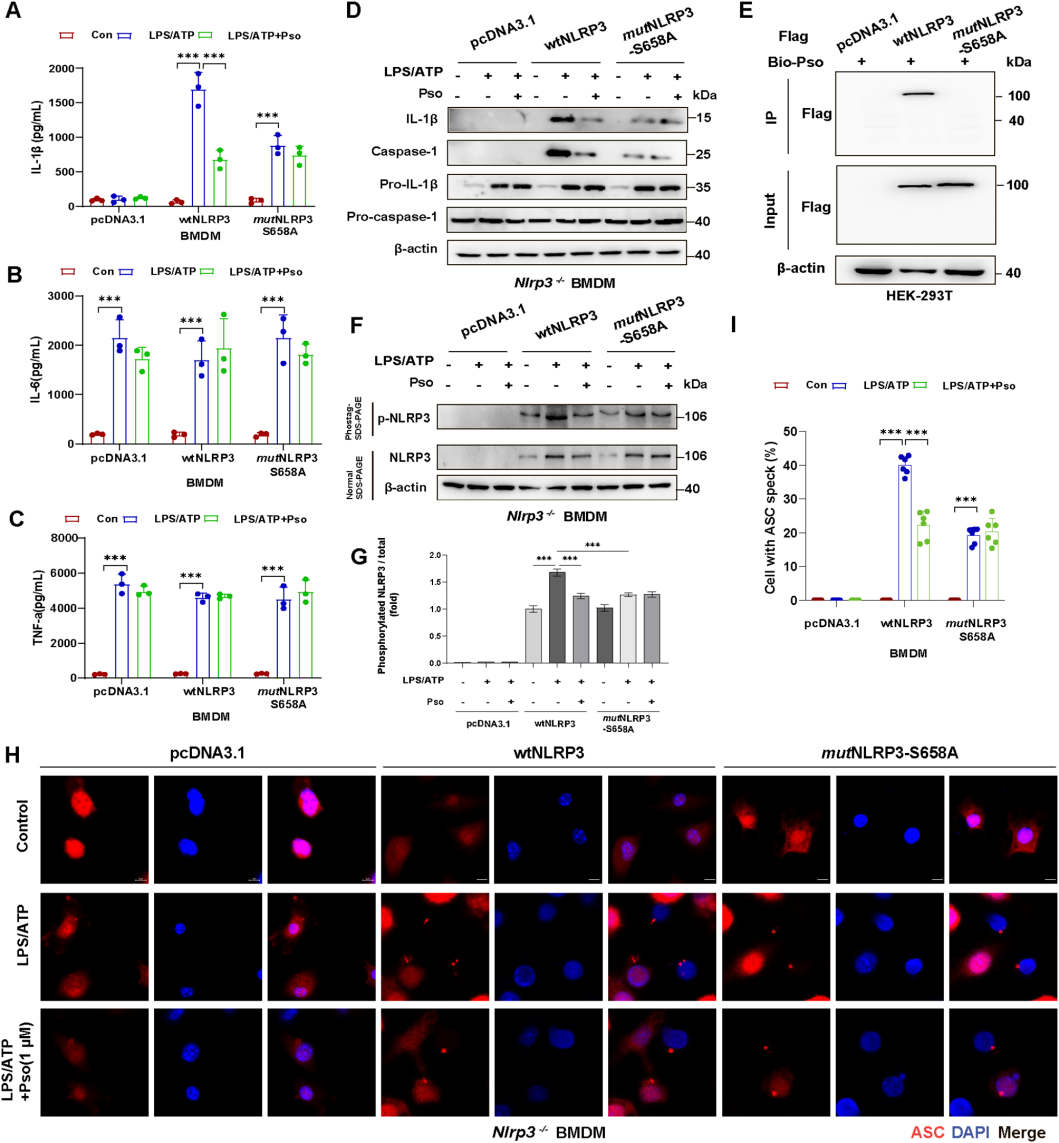
S658A mutation blocks the inhibitory effect of Psoralen on NLRP3 inflammasome activation. Effects of Psoralen treatment on the release of IL-1β **(A)**, IL-6 **(B)**, and TNF-α **(C)** from LPS + ATP-stimulated WT and S658A mutant BMDMs. **(D)** IL-1β and caspase-1 from SN and pro-IL-1β/pro-caspase-1 from Lysates were analyzed by immunoblotting after BMDMs were transfected with wtNLRP3 or *mut*NLRP3-S658A plasmids. **(E)** Immunoblots showed the binding of Pso and wtNLRP3 but not Pso and mutNLRP3-S658A in HEK-293T cells. **(F-G)** Phos-tag assay and quantification of NLRP3 phosphorylation in BMDMs pretreated with Pso followed by transfection with wtNLRP3 or *mut*NLRP3-S658A plasmids. **(H-I)** Immunostaining and quantification for ASC speck (red) in *Nlrp3* KO BMDMs pretreated with Pso followed by transfection with wtNLRP3 or *mut*NLRP3-S658A plasmids. DAPI stains the nucleus (blue). The scale bar represents 5 μm. Data were analyzed by one-way ANOVA, followed by Tukey post-tests. ^*^P < 0.05, ^**^P < 0.01 and ^***^P < 0.001

Next, we assessed the impact of Pso treatment on NLRP3 inflammasome activation in BMDMs transfected with either WT-NLRP3 or the S658A mutant. Pso treatment suppressed LPS + ATP-induced IL-1β release in WT BMDMs, but not in BMDMs expressing the S658A mutant (Fig. 7A). Similar to earlier findings, Pso treatment had no effect on IL-6 or TNF-α release in either WT or S658A mutant BMDMs (Fig. 7B-C). Western blotting further showed that Pso treatment reduced the expression of IL-1β and caspase-1 in LPS + ATP-stimulated WT BMDMs, but this effect was abolished in the S658A mutant BMDMs (Fig. 7D). Importantly, the S658A mutation also prevented Pso from binding to NLRP3, abolishing its inhibitory effect on NLRP3 phosphorylation (Fig. 7E-G). Consequently, Pso treatment failed to inhibit the assembly of the NLRP3 inflammasome in the S658A mutant, as demonstrated by immunofluorescence staining of ASC specks (Fig. 7H-I).

Furthermore, to understand the relative potency of Pso, MCC950 was included in the following experiments supplemented in Fig.S12. As shown in Fig. S12A, we observed that treatment of Pso (1 µM) inhibited the expression of caspase-1 and IL-1β in BMDMs incubated with LPS and ATP. Subsequently, we assessed the comparative efficacy of Pso and MCC950 at an identical concentration. Specifically, Pso demonstrated comparable efficacy at a concentration of 10 µM to that observed at 1 µM. In contrast, MCC950 at 10 µM exhibited a greater inhibitory potency on IL-1β release than Pso in WT BMDMs. We further delineated the effects of Pso and MCC950 on NLRP3 inflammasome activation by overexpression with the S658 mutant in *Nlrp3*^*−/−*^ BMDMs. We observed that mutation of NLRP3 S658A abrogated the downregulation of caspase-1 and IL-1β levels by Pso treatment, whereas mutation of NLRP3 S658A failed to influence the effect of MCC950 on NLRP3 inflammasome (Fig.S12C-D). These results indicate that phosphorylation at Serine 658 is a potential target for the inhibitory effect of Pso, not for MCC950 on NLRP3 inflammasome activation.

As we discovered that phosphorylation site S658 at NLRP3 is crucial for the activation of NLRP3, we further want to uncover the kinases or phosphatases that target S658 at NLRP3 phosphorylation. From our proteomics data, GO enrichment analysis indicated there were significantly elevated protein kinases or phosphatases (*Nrp2*, *Xdh*, *Mapk8*, *Anxa2*, and *Cox7a1*) in the PFF group compared with the Control group (Fig.S13A). These five protein kinases or phosphatases (*Nrp2*, *Xdh*, *Mapk8*, *Anxa2*, and *Cox7a1*) were knockdown in the BMDMs and the efficiency of small interfering RNA (*Nrp2*, *Xdh*, *Mapk8*, *Anxa2*, and *Cox7a1)* is validated by RT-PCR (Fig.S13B). Phos-tag SDS-PAGE and quantification of the phosphorylation levels of NLRP3 showed the knockdown of *Mapk8* and *Nrp2* can significantly reduce the phosphorylation levels of NLRP3 (*P* < 0.01, Fig.S13C-D). Therefore, *Mapk8* and *Nrp2* are selected for further study. To explore whether *Mapk8* or *Nrp2* targeted S658 phosphorylation in NLRP3 inflammasome activation, non-phosphorylatable S658A mutant (S658A) along with WT-NLRP3, were transfected into *Nlrp3* KO BMDMs. Phos-tag SDS-PAGE results revealed that the knockdown of *Mapk8* still induced significantly downregulated phosphorylation levels of NLRP3 in the S658A mutant group as well as the WT-NLRP3 group. However, the knockdown of *Nrp2* dramatically abolished the deduced phosphorylation of NLRP3 in the S658A mutant group. Overall, we speculated that *Nrp2*,* not* JNK/Mapk8 may be involved in the S658 phosphorylation site of NLRP3 (Fig.S13E-F).

Taken together, our findings demonstrate that Psoralen inhibits NLRP3 inflammasome activation by binding to NLRP3 and suppressing its phosphorylation at Serine 658, highlighting a novel regulatory mechanism that could be therapeutically targeted in diseases associated with NLRP3 inflammasome activation.

### Inhibition of NLRP3 phosphorylation at Serine 658 improves PD-like motor symptoms and pathology in the MPTP/p model

**Fig. 8:**
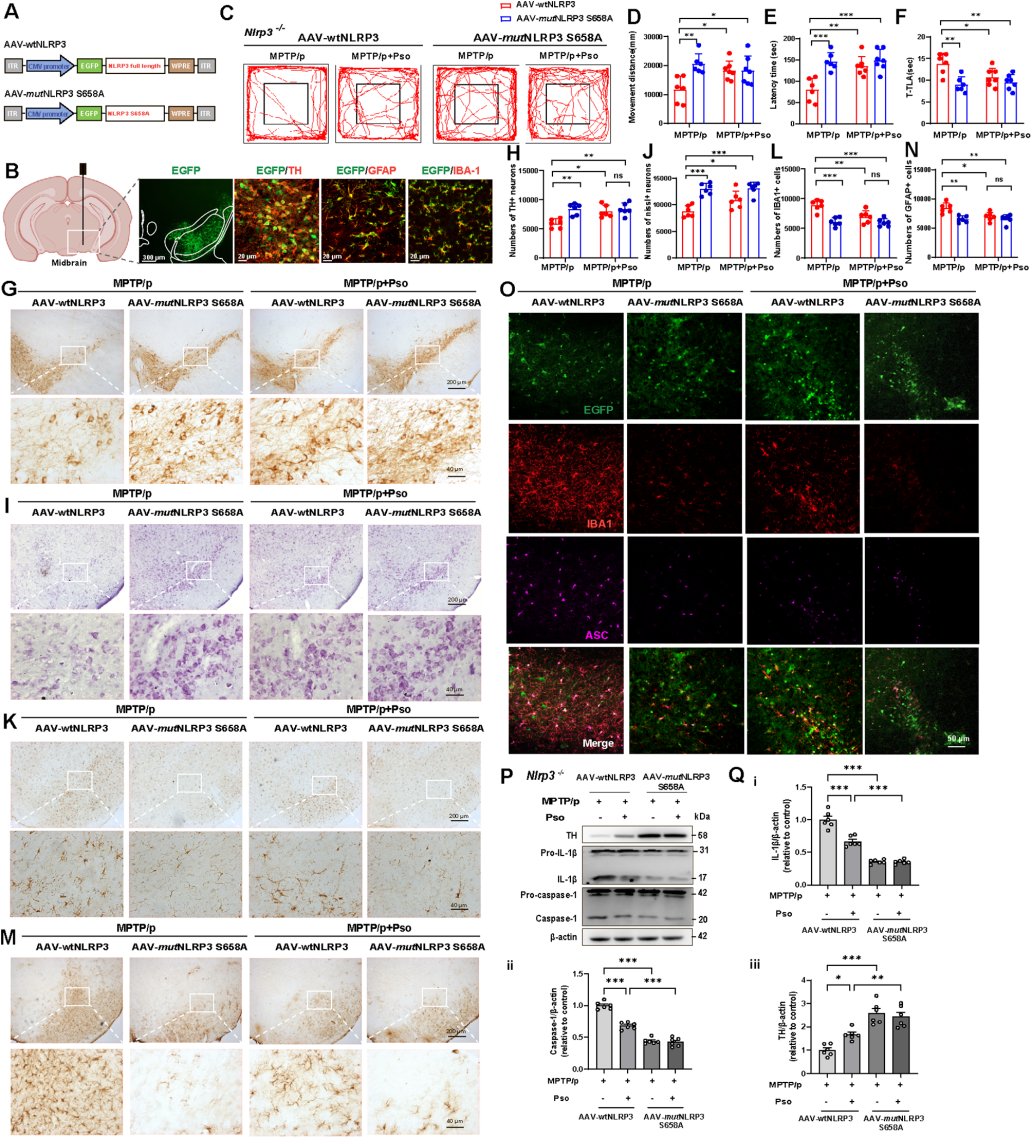
Inhibition of NLRP3 phosphorylation at Serine 658 improves PD-like motor symptoms and pathology in the MPTP/p model. **(A)** Schematic illustration of AAVs carrying NLRP3 WT-EGFP (AAV-wtNLRP3) or NLRP3 S658A-EGFP (AAV-mutNLRP3). **(B)** Representative images showing co-label immunofluorescence of AAV(NLRP3-EGFP) with dopaminergic neuron marker (TH), astrocytes marker (GFAP), and microglia marker (IBA-1). Scale bar: 300 μm. Enlarge vision: 300 μm. Motor performance of *Nlrp3* KO mice injected with AAV-wtNLRP3 or AAV-mutNLRP3 followed by treatment of MPTP/p and Psoralen in the open field test **(C-D)**, the rotarod test **(E)**, and the pole test **(F)**, *n* = 6. **(G-H)** Representative immunohistochemical images and quantification of TH-positive neurons in the substantia nigra compacta. **(I-J)** Representative immunohistochemical images and quantification of Nissl^+^ neurons in the substantia nigra compacta. **(K-L)** Representative immunohistochemical images and quantification of IBA1^+^ cells in the substantia nigra compacta. **(M-N)** Representative immunohistochemical images and quantification of GFAP^+^ cells in the substantia nigra compacta, *n* = 6. Scale bars, 200 μm. Enlarge vision: 40 μm. **(O)** Immunofluorescence staining for NLRP3 (green), IBA1(red), and ASC (purple) in the substantia nigra compacta of *Nlrp3* KO mice. The scale bar represents 50 μm. **(P-Q)** The protein levels of TH, IL-1β, and caspase-1 from the striatum were analyzed by immunoblotting, *n* = 3. Data were analyzed by two-way ANOVA, followed by Tukey post-tests. ^*^P < 0.05, ^**^P < 0.01 and ^***^P < 0.001. ns: no significance

To further investigate the therapeutic potential of targeting NLRP3 phosphorylation at Serine 658 in PD, *Nlrp3* KO mice were injected with adeno-associated viruses (AAV) carrying either WT NLRP3 (AAV-WT-NLRP3) or the S658A mutant NLRP3 (AAV-Mut-NLRP3_S658A). The mice were then subjected to the MPTP/p model of PD (Fig. 8A). Viral infections targeted the midbrain, and the infected efficiency was confirmed by co-label immunofluorescence of enhanced green fluorescent protein (EGFP) with dopaminergic neuron marker (TH), astrocytes marker (GFAP), and microglia marker (IBA-1) (Fig. 8B). In behavioral tests, such as the open field test, S658A mutation significantly improved the movement distance of MPTP/p-treated mice. However, Pso treatment increased movement only in MPTP/p-treated WT mice but not in mice carrying the S658A mutation (Fig. 8C-D). The results were consistent in the rotarod and pole tests, demonstrating that the inhibition of NLRP3 phosphorylation at S658 can ameliorate PD-like motor deficits (Fig. 8E-F). Histological analysis through TH and Nissl staining indicated that Pso treatment rescued DA neuronal loss in the SNc region of WT but not in S658A mutant mice. The effects observed in the S658A mutant group were similar to those of Pso treatment in WT mice, confirming that blocking S658 phosphorylation has a protective effect on DA neurons (Fig. 8G-J). In addition to protecting DA neurons, S658A mutation also reduced microglial and astrocytic reactivity in the SNc region of MPTP/p-treated mice. Likewise, Pso treatment reduced glial reactivity in WT mice, but had no effect in the S658A mutant mice (Fig. 8K-N). Immunofluorescence staining of ASC specks in combination with IBA1 demonstrated that the assembly of NLRP3 inflammasome in microglia was blocked by both the S658A mutation and Pso treatment (Fig. 8O). In the striatum region of MPTP/p-treated mice, both Pso treatment and S658A mutation restored TH expression, which was otherwise reduced due to MPTP/p-induced neurodegeneration. Furthermore, the levels of IL-1β and caspase-1 were significantly reduced by both Pso treatment and S658A mutation (Fig. 8P-8Q_i − iii_). These results collectively demonstrate that phosphorylation of NLRP3 at Serine 658 drives NLRP3 inflammasome assembly and activation in glial cells, promoting neuroinflammation and accelerating PD progression. Psoralen binds to NLRP3 and prevents its phosphorylation at Serine 658, effectively blocking NLRP3 inflammasome activation and neuroinflammation, thereby mitigating PD-like motor symptoms and neurodegeneration. This highlights the therapeutic potential of Psoralen in PD treatment by targeting the NLRP3 phosphorylation (Fig. S14).

## Discussion

Research into the role of inflammasome activation-mediated neuroinflammation in PD has intensified interest in targeting NLRP3 inflammasome for therapeutic intervention [25, 29]. In addition to the discovery of new drugs, recent studies have explored the therapeutic benefits of natural products in PD by inhibiting NLRP3 inflammasome activation [30]. In our study, we conducted a virtual screening of a natural product library comprising 5,088 compounds, and identified Psoralen as a potent NLRP3 inflammasome inhibitor. Psoralen selectively inhibited NLRP3 inflammasome activation by binding to the NACHT and LRR domains of the NLRP3 protein, without affecting NLRP1, AIM2, or NLRC4. It specifically blocked NLRP3 phosphorylation at Serine 658. Importantly, Psoralen treatment alleviated PD-like motor symptoms and dopaminergic neuronal degeneration by inhibiting NLRP3 phosphorylation and inflammasome activation in glial cells. Our study unveils a novel phosphorylation site (S658) on NLRP3 and introduces Psoralen as an inhibitor, offering a promising therapeutic target and strategy for diseases driven by NLRP3 inflammasome.

Growing evidence supports that NLRP3 inflammasome plays a crucial role in central neuroinflammation and neurodegeneration in PD [10, 31]. This has led to the development of various inhibitors targeting NLRP3 inflammasome for PD treatment. For instance, MCC950 and Glibenclamide have been identified as effective inhibitors, slowing disease progression in PD mouse models [32, 33]. In addition, it has been reported that Cleaved caspase-1 and the inflammasome adaptor protein apoptosis-associated speck-like protein containing a C-terminal caspase recruitment domain (ASC) were elevated in the substantia nigra of PD patients [34]. Besides, exome sequencing data for genetic variation of NLRP3 identified multiple single-nucleotide polymorphisms (SNPs) including rs7525979, indicating it was associated with a significantly reduced risk of developing PD [35]. Findings suggest plasma-borne inflammasome-related proteins as a potentially useful class of biomarkers for PD [36]. More importantly, several NLRP3 inhibitors have been reported in clinical research. VTX3232, a CNS-penetrant NLRP3 Inhibitor, is conducting a Phase 2a clinical study to evaluate the safety, tolerability, pharmacokinetics, and pharmacodynamics in participants with Early-Stage Parkinson’s Disease [37]. Selnoflast (RO7486967), formerly named somalix/RG6418/IZD334, an orally potent, selective, and reversible small molecule NLRP3 inflammasome inhibitor, has completed the Phase 1b study in participants with Early Idiopathic Parkinson’s Disease [38]. Despite the discovery and testing of several NLRP3 inflammasome inhibitors in cellular and animal models, no drugs targeting NLRP3 inflammasome are yet available on the market [17, 25, 39]. Natural products, with their high molecular diversity and unique bioactivity, are vital sources for drug discovery, offering enhanced efficacy and safety [16]. Consequently, we screened a natural product library of 5,088 compounds for NLRP3 inflammasome inhibitors. Psoralen emerged as the most effective candidate based on affinity and efficacy tests. A naturally occurring phytoalexin in Psoralea corylifolia seeds [40], Psoralen is used clinically in various treatments, often with UVA exposure [41, 42], such as for psoriasis, a chronic inflammatory skin condition [43]. In renal fibrosis research, Psoralen showed therapeutic effects by inhibiting NLRP3 inflammasome activation [44]. Here, we report for the first time the therapeutic effect of Psoralen in PD through the inhibition of glial NLRP3 inflammasome activation. Unlike traditional photoactivated Psoralen applications, we administered Psoralen intragastrically in mice. Our findings demonstrate that Psoralen can cross the blood-brain barrier and inhibit NLRP3 inflammasome activation in microglia and astrocytes, broadening its potential applications.

In the central nervous system, the persistent hyperactivation of inflammasomes such as NLRP1, AIM2, NLRP3, and NLRC4 contributes to neurodegeneration [10]. By overexpressing these inflammasomes in HEK-293T cells, we observed that Psoralen binds exclusively to NLRP3, targeting its NACHT and LRR domains. Numerous studies have shown that NLRP3 inflammasome-mediated neuroinflammation predominantly occurs in microglia and astrocytes [45, 46]. Accordingly, we evaluated the inhibitory effects of Psoralen on NLRP3 inflammasome in primary cultured microglia and astrocytes using two well-established activation models. Results consistently demonstrated the efficacy of Psoralen in inhibiting NLRP3 inflammasome activation in these glial cells. In an indirect co-culture system, conditioned medium from Psoralen-pretreated microglia and astrocytes protected against dopaminergic neuronal damage induced by conditioned medium from NLRP3 inflammasome activated glial cells. Recent studies have highlighted the role of neuronal NLRP3 inflammasome in neuroinflammation regulation [13, 47]. To assess the direct impact of Psoralen on neuronal survival, SH-SY5Y cells were pretreated with Psoralen followed by MPP^+^ stimulation. Psoralen treatment did not affect cell viability or LDH release under basal and MPP^+^ conditions, suggesting its neuroprotective effects are mediated through inhibition of glial NLRP3 inflammasome activation, rather than direct neuronal effects. Thus, our study demonstrates that by binding to the NACHT and LRR domains of NLRP3, Psoralen inhibits glial NLRP3 inflammasome activation to provide neuroprotection. The selectivity of Psoralen in inhibiting NLRP3 inflammasome activation presents a novel targeted approach to mitigate related inflammation, potentially avoiding the risks associated with general immunosuppressive strategies.

Post-translational modifications of the NLRP3 protein, including ubiquitination, phosphorylation, sumoylation, and acetylation, are crucial for inflammasome assembly and activation [8]. For instance, phosphorylation at Serine 194 initiates inflammasome activation [20], while phosphorylation at Serine 803 prevents it [21]. In this study, Psoralen treatment did not alter total NLRP3 expression but significantly reduced its phosphorylation level. This prompted further investigation into NLRP3 phosphorylation under Psoralen treatment. Using 4D label-free quantitative phosphorylation proteomics, we identified Serine 658 (S658) as a phosphorylation site blocked by Psoralen. Additionally, the non-phosphorylatable S658A mutant hindered NLRP3 inflammasome assembly and activation, nullifying Psoralen’s inhibitory effects. Our in vivo experiments revealed that inhibiting phosphorylation at S658, either by S658A mutation or Psoralen treatment, provided therapeutic benefits in PD. These results suggest that Psoralen prevents NLRP3 phosphorylation at S658, thus inhibiting inflammasome activation. However, although our data indicated *Nrp2*,* not* JNK/Mapk8 may be involved in the S658 phosphorylation site of NLRP3, it is too early to conclude that *Nrp2* is targeted in the phosphorylation of NLRP3 at Serine 658 (Fig. S13). More research is still needed to take a deep dive into the detailed mechanism. Therefore, our research identifies a novel phosphorylation site on the NLRP3 protein that positively regulates inflammasome assembly and activation, presenting an effective drug target for inhibiting inflammasome activation.

## Conclusion

In summary, our study provides new insights into the mechanism of NLRP3 inflammasome activation and introduces a novel inhibitor. However, further research is needed to explore the intricate phosphorylation processes of NLRP3. Future studies should identify potential kinases and phosphatases targeting NLRP3 at S658. Additionally, the long-term safety, efficacy, and potential off-target effects of Psoralen should be evaluated in PD animal models to fully understand its therapeutic potential and safety profile. Nonetheless, our work marks a significant advancement in discovering highly selective NLRP3 inflammasome inhibitors, offering new strategies for treating NLRP3 inflammasome-related diseases.

## Electronic supplementary material

Below is the link to the electronic supplementary material.

Supplementary Material 1

Supplementary Material 2

## Data Availability

The data supporting the findings of this study are available from the corresponding authors upon reasonable request.

## Abbreviations

AIM2: Absent in melanoma 2
BMDMs: Bone marrow-derived macrophages
Bio-Ori: Biotinylated oridonin
DA: Dopaminergic
EGFP: Enhanced green fluorescent protein
EPM: Elevated plus maze
HPLC: High-performance liquid chromatography
KEGG: Kyoto Encyclopedia of Genes and Genomes
KO: Knockout
LDH: Lactate dehydrogenase
NLRs: Nucleotide-binding oligomerization domain-like receptors
PD: Parkinson’s disease
PRRs: Pattern recognition receptors
Pso: Psoralen
SPF: Specific pathogen-free
SPR: Surface plasmon resonance
TLR: Toll-like receptor
UPLC–MS: Ultra-performance liquid chromatography-mass spectrum
WT: Wild-type
