# Supplementary material for: Inactivation of NLRP3 inflammasome by dephosphorylation at Serine 658 alleviates glial inflammation in the mouse model of Parkinson’s disease
Source: Mol Neurodegener. 2025 Mar 5;20:27. doi: 10.1186/s13024-025-00818-z (PMC11881452; doi:10.1186/s13024-025-00818-z)

FIG1 E

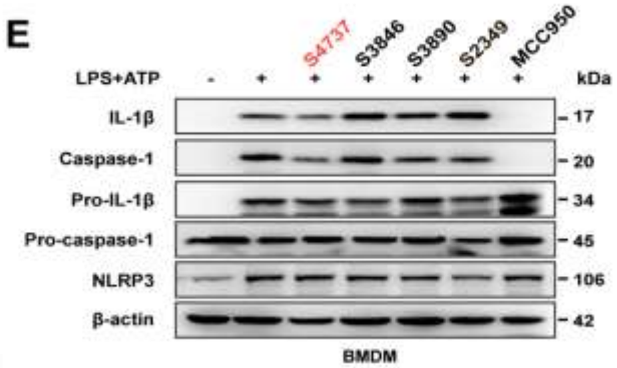
E
S4737
S3846
S3890
S2349
MCC950
LPS+ATP - + + + + + +
kDa
IL-1β - 17
Caspase-1 - 20
Pro-IL-1β - 34
Pro-caspase-1 - 45
NLRP3 - 106
β-actin - 42
BMDM

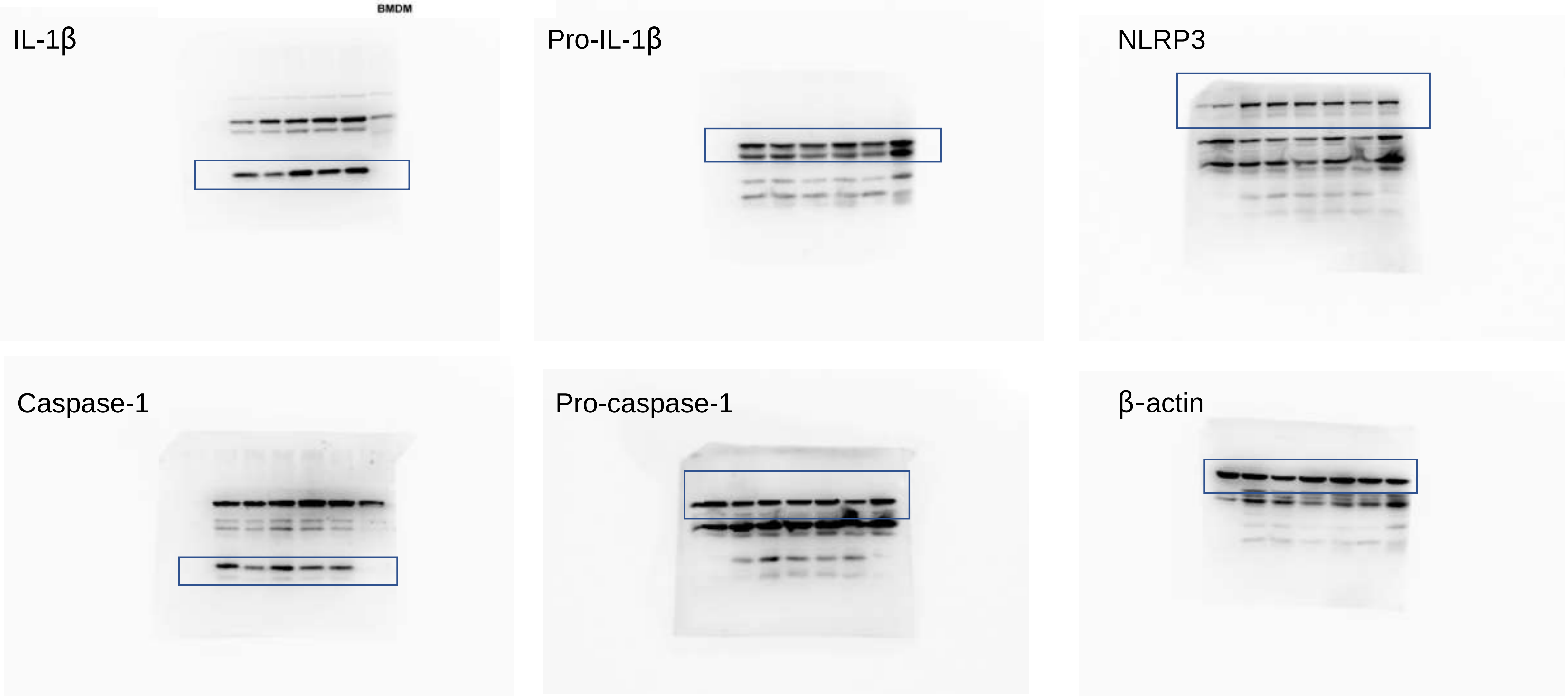
IL-1β
Pro-IL-1β
NLRP3
Caspase-1
Pro-caspase-1
β-actin

FIG1 G

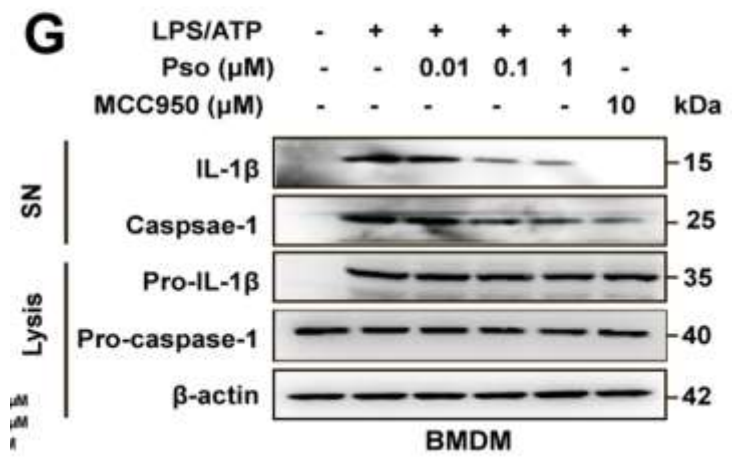

Pro-IL-1β

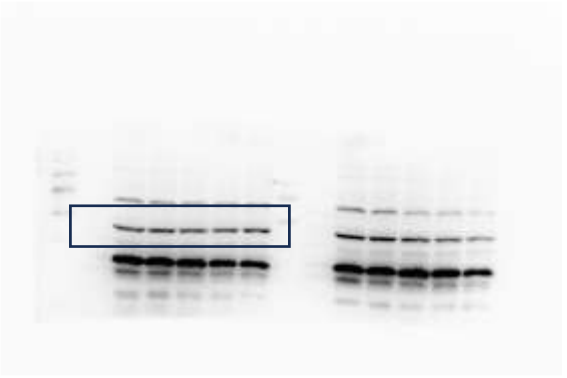

Pro-caspase-1

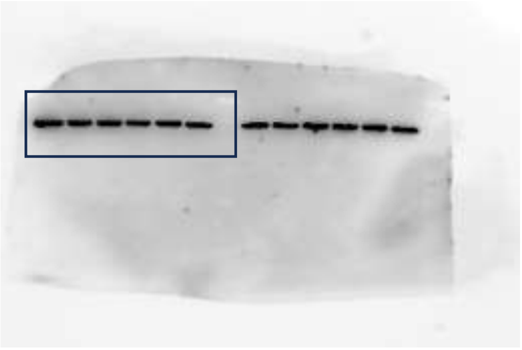

IL-1β

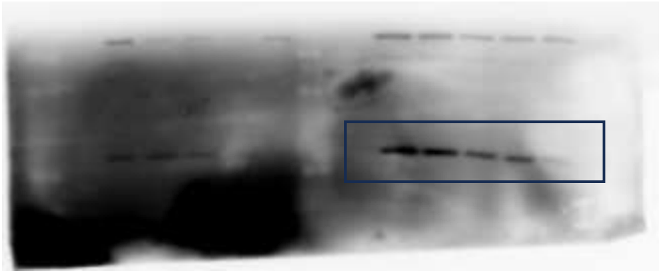

β-actin

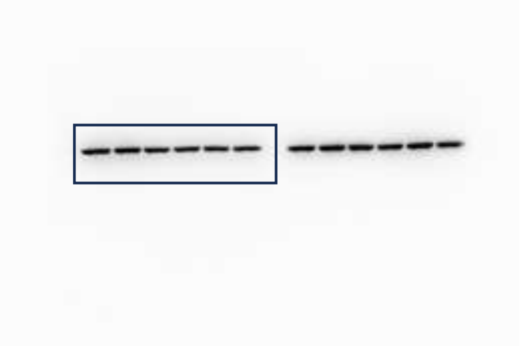

Caspase-1

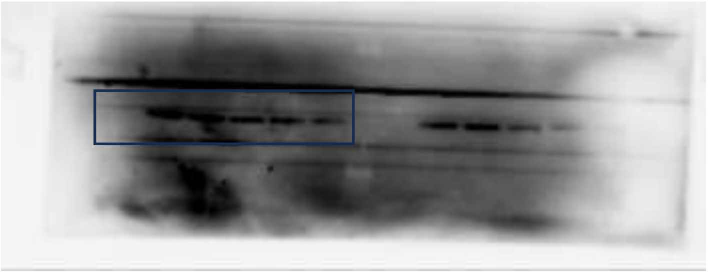

FIG1 H

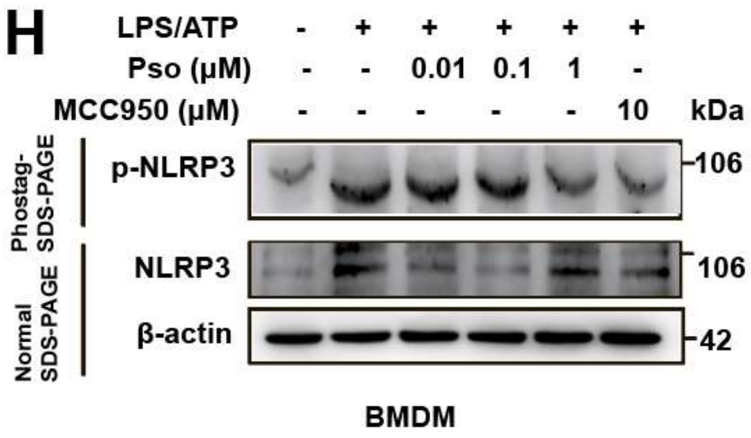

NLRP3

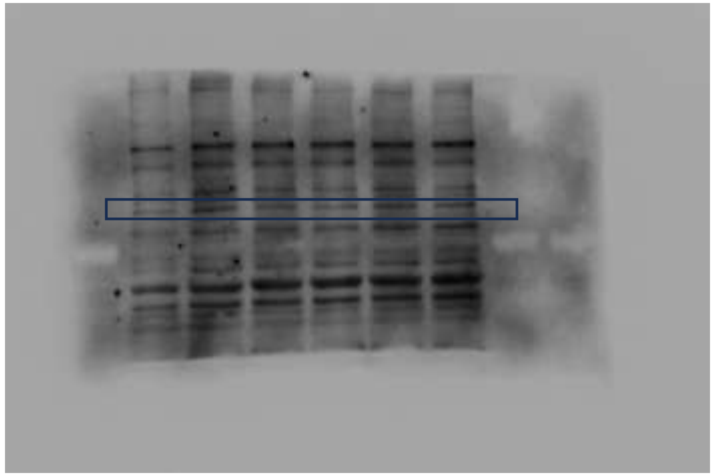

p-NLRP3

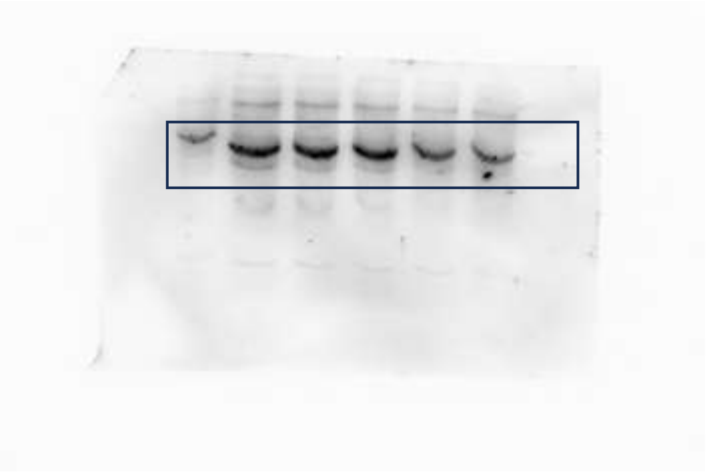

β-actin

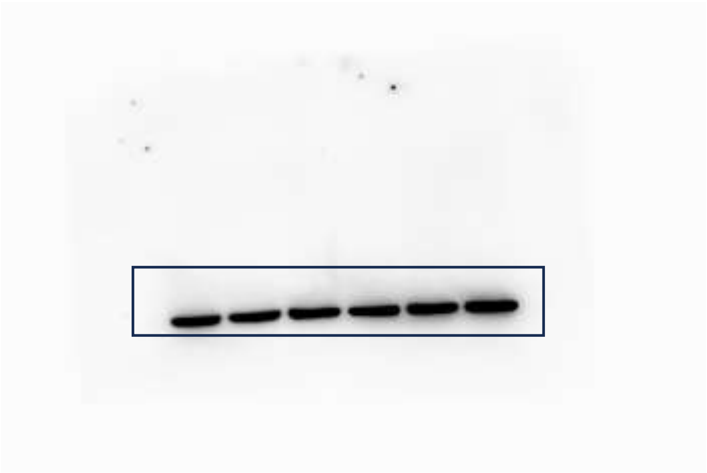

FIG1 I

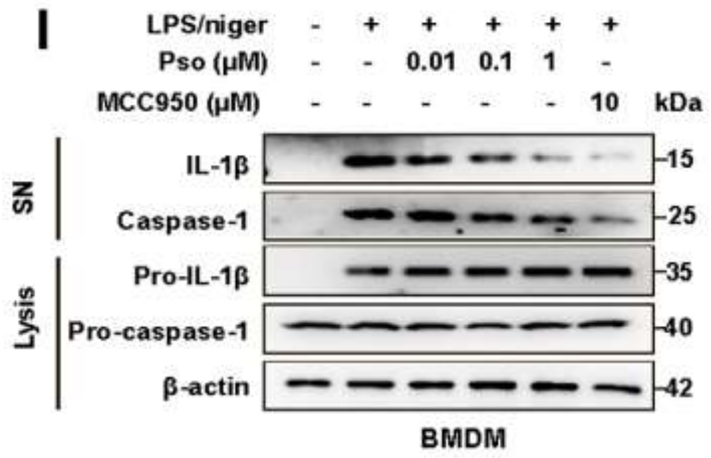

IL-1β

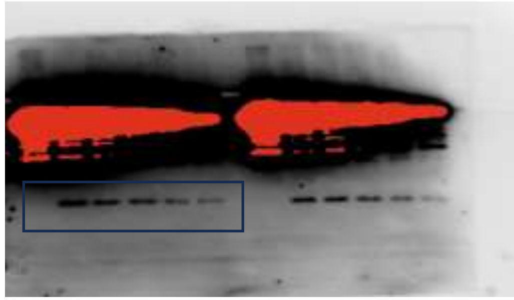

Caspase-1

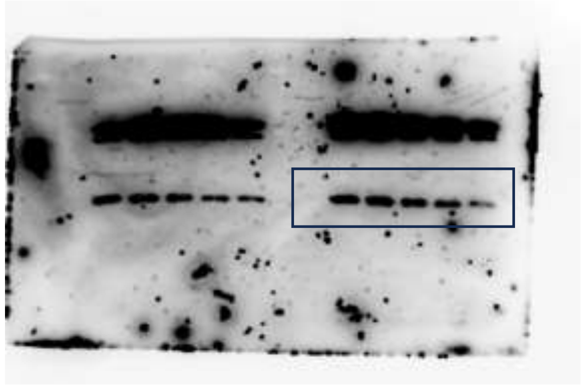

Pro-IL-1β

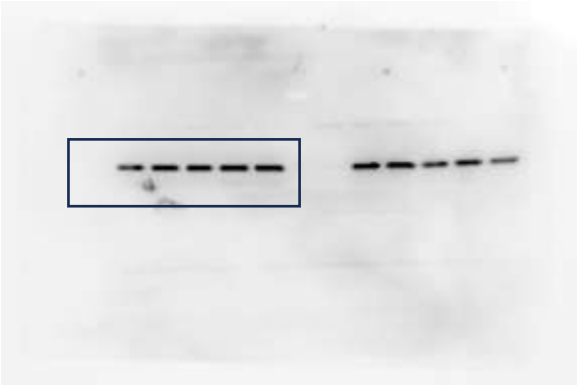

Pro-Caspase-1

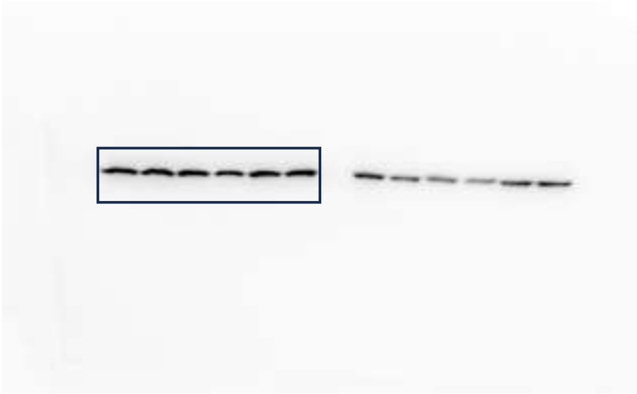

β-actin

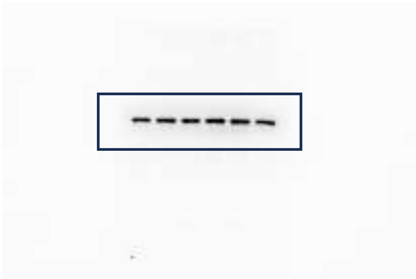

FIG1 J

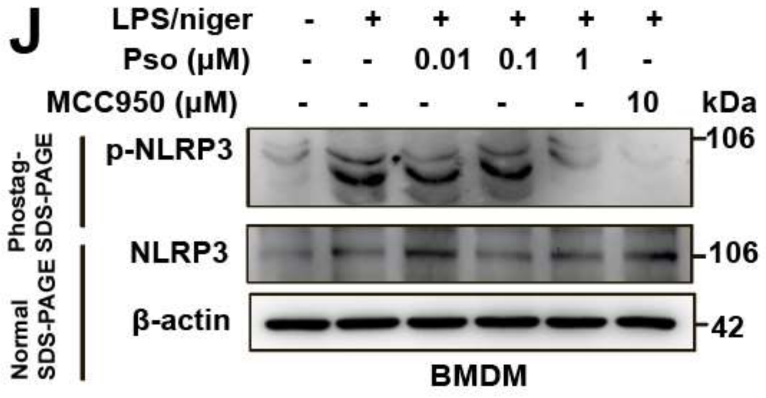

NLRP3

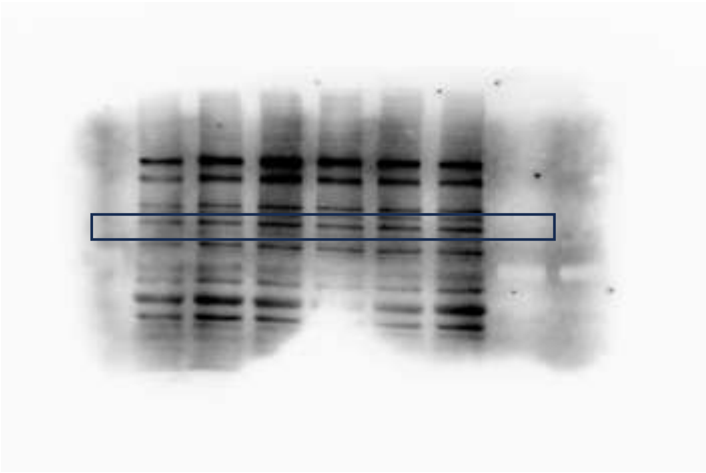

p-NLRP3

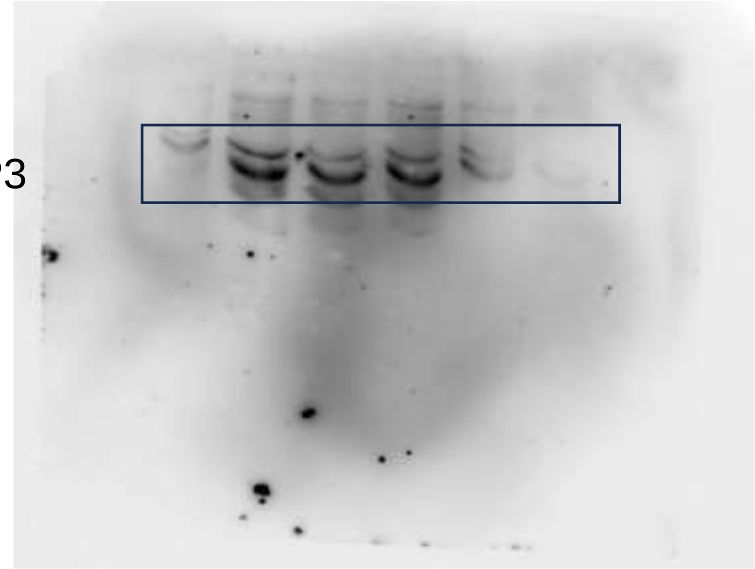

β-actin

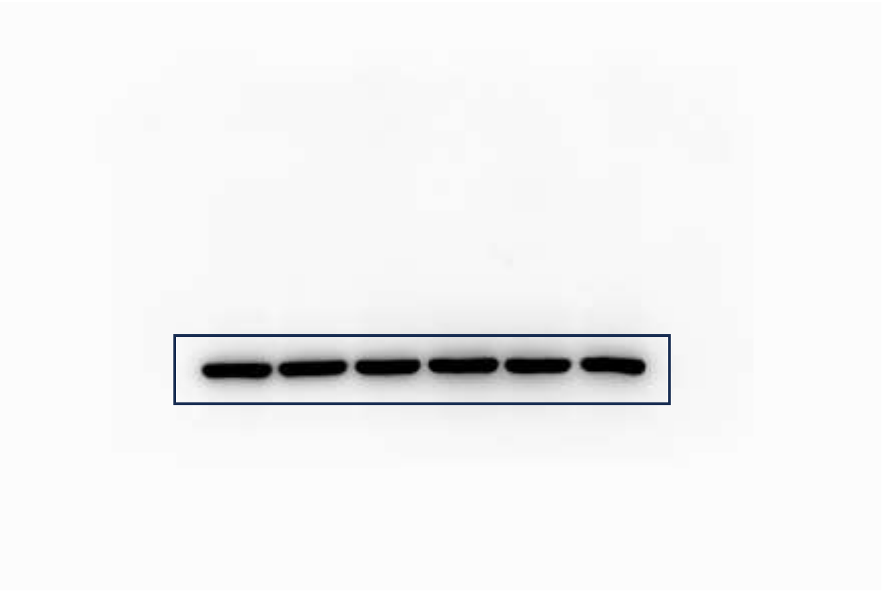

FIG2 A

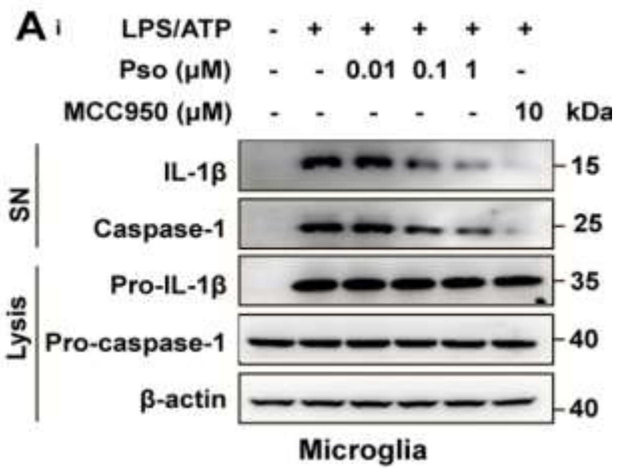

IL-1β

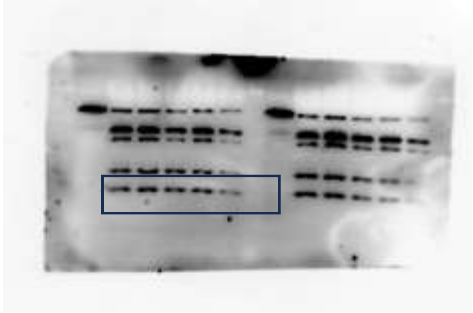

Caspase-1

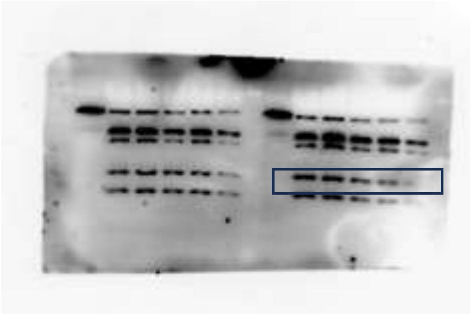

β-actin

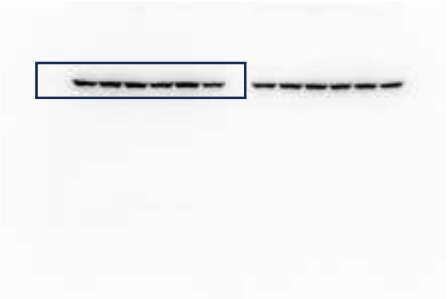

Pro-IL-1β

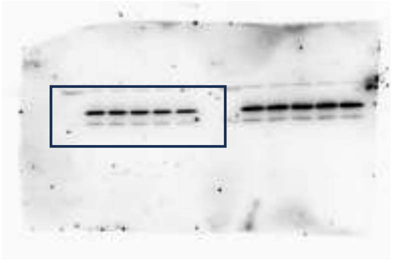

Pro-caspase-1

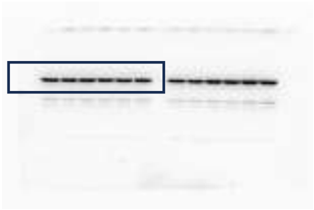

FIG2 B

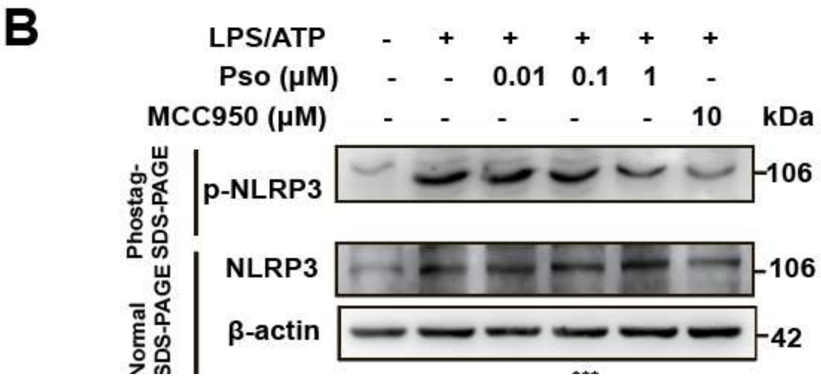

NLRP3

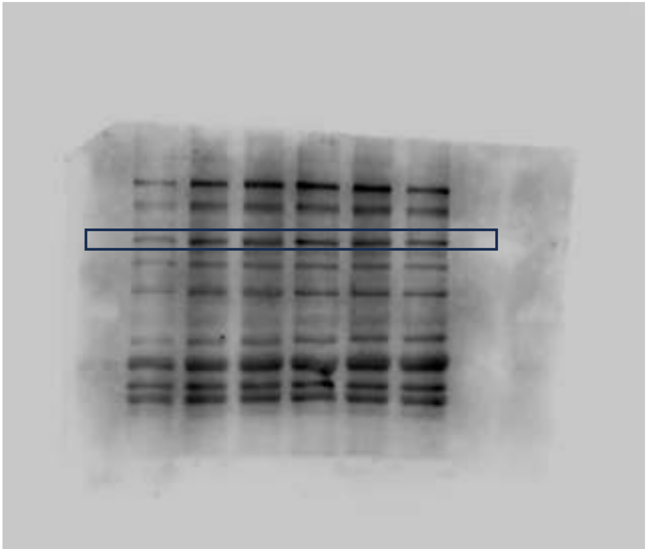

p-NLRP3

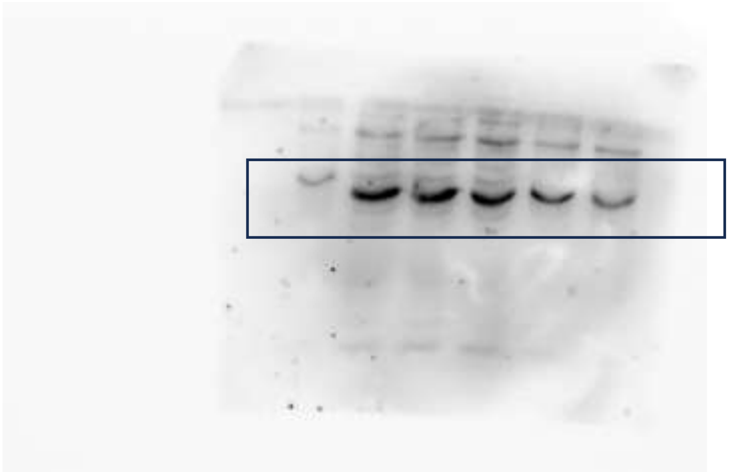

β-actin

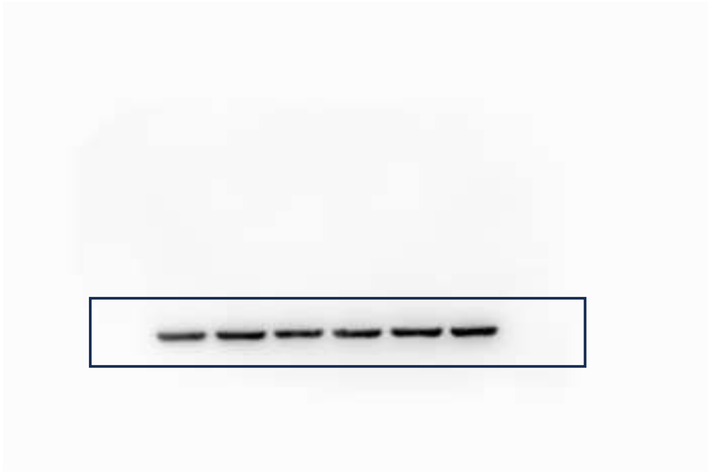

FIG2 F

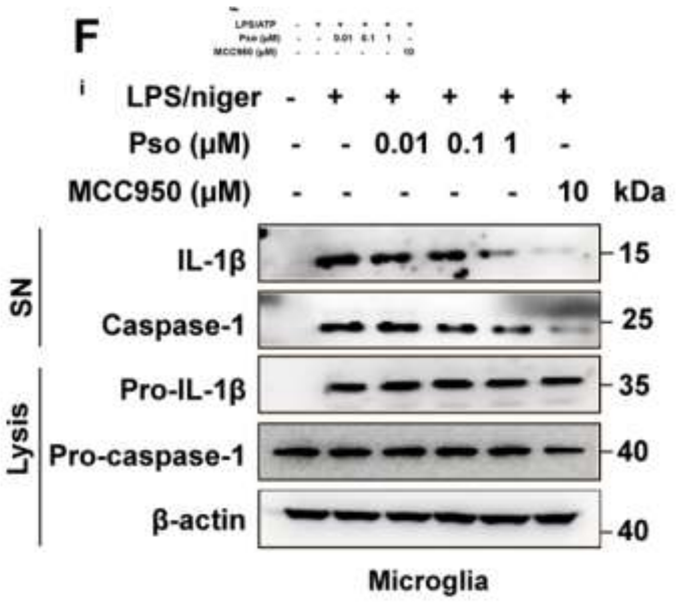

IL-1β

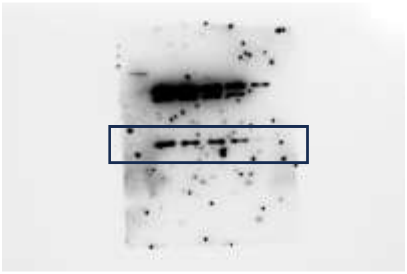

Pro-IL-1β

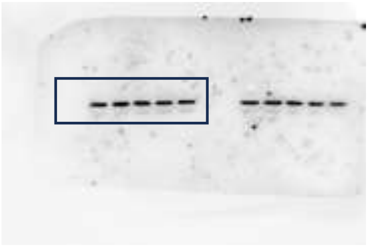

Caspase-1

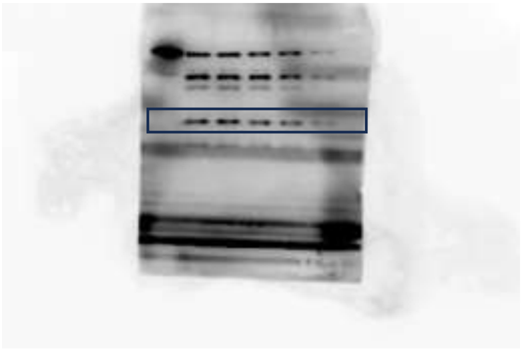

Pro-caspase-1

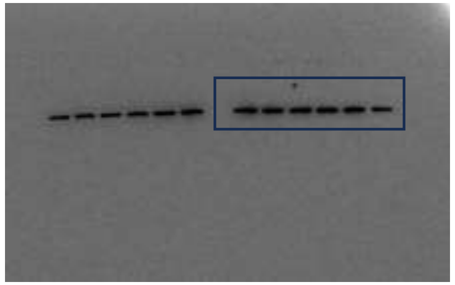

β-actin

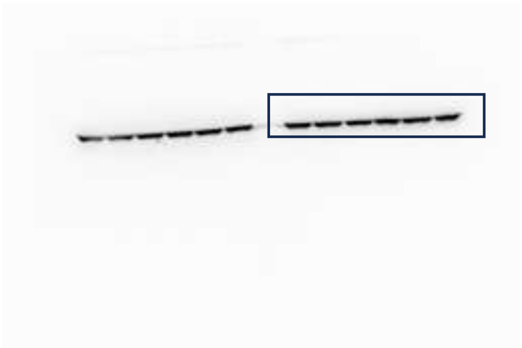

FIG2 G

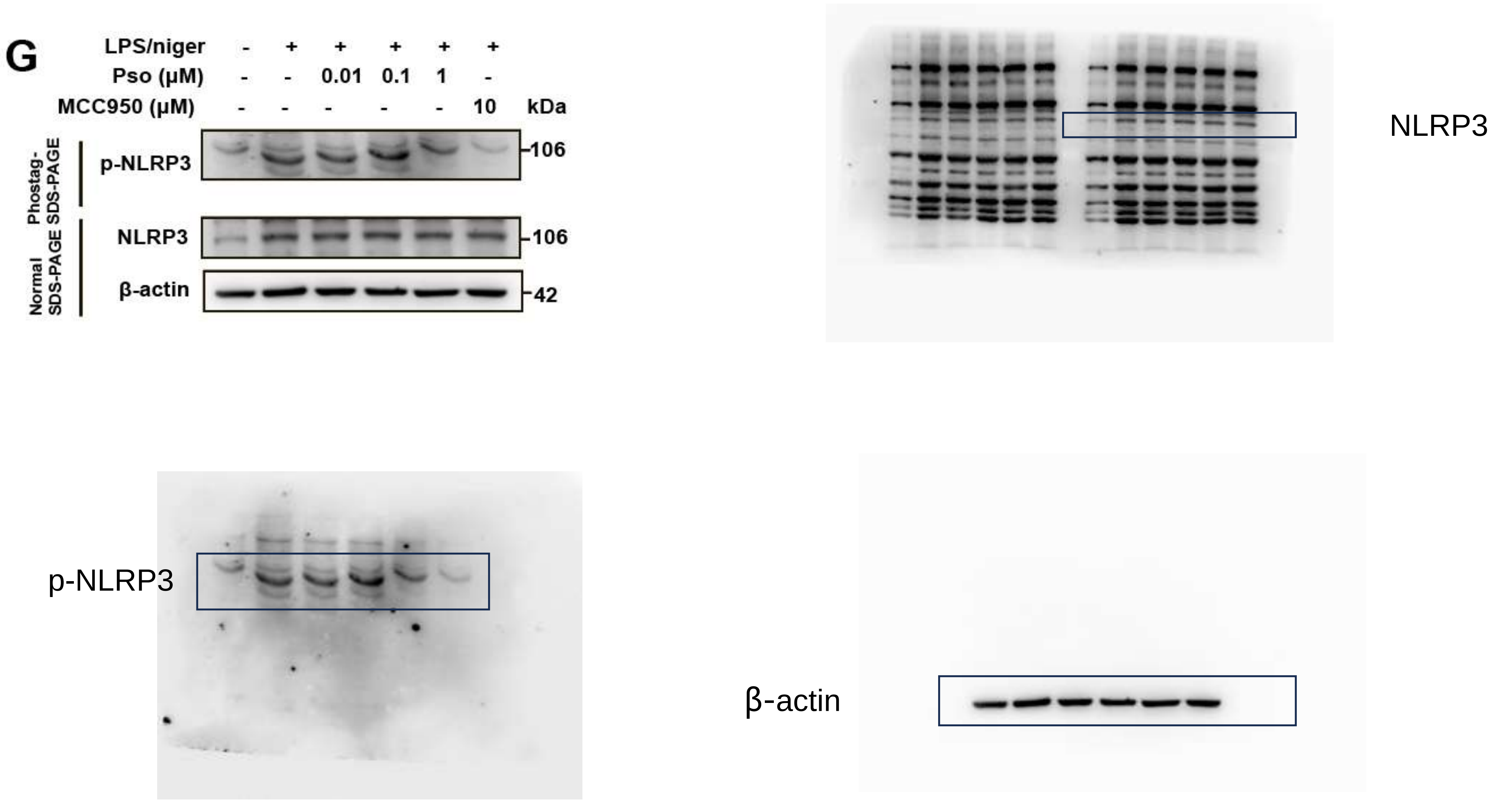
G
LPS/niger - + + + + +
Pso (μM) - - 0.01 0.1 1 -
MCC950 (μM) - - - - - 10
kDa
Phostag-SDS-PAGE
p-NLRP3
106
Normal SDS-PAGE
NLRP3
106
β-actin
42
NLRP3
p-NLRP3
β-actin

FIG2 H

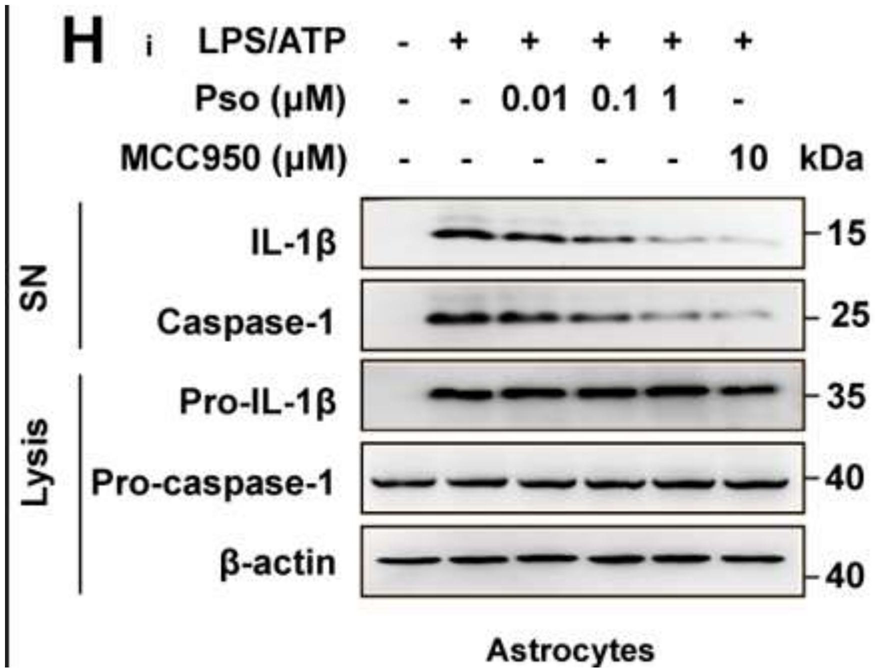

IL-1β

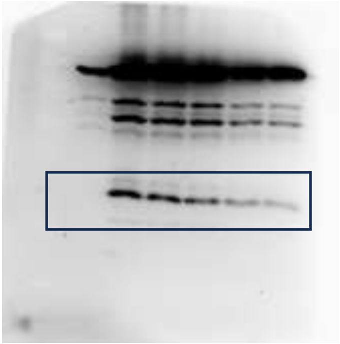

Pro-IL-1β

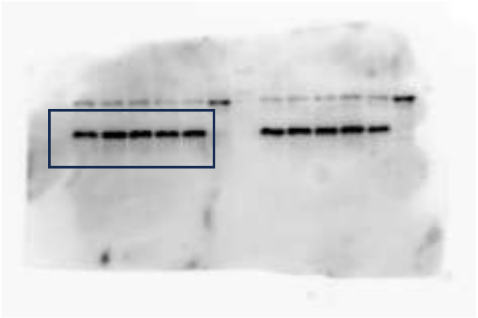

Caspase-1

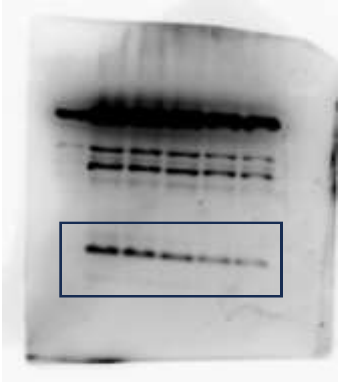

Pro-caspase-1

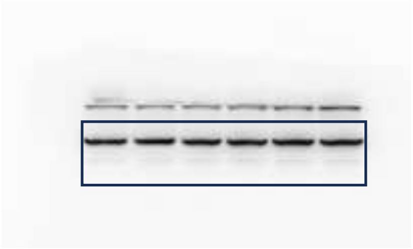

β-actin

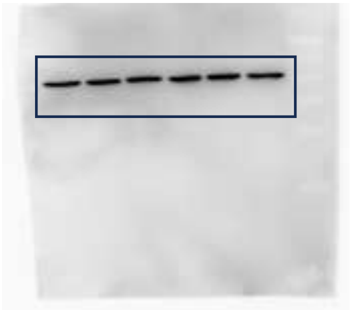

FIG2 I

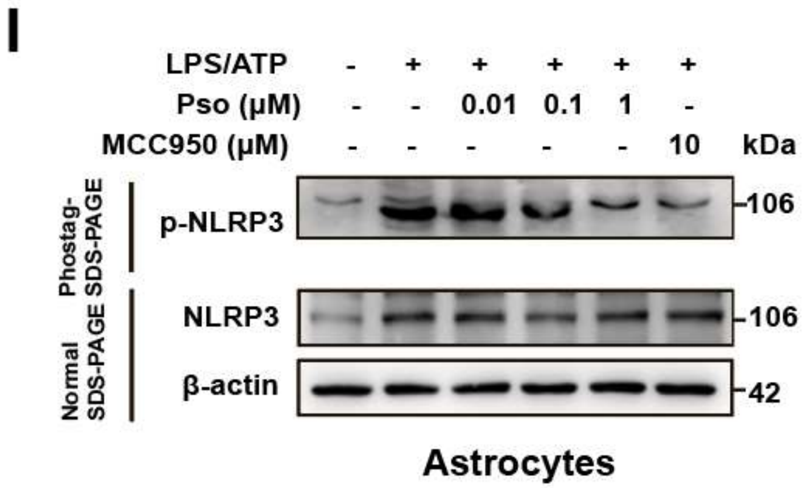
I
LPS/ATP - + + + + +
Pso (μM) - - 0.01 0.1 1 -
MCC950 (μM) - - - - - 10
kDa
Phostag-SDS-PAGE
p-NLRP3
106
Normal SDS-PAGE
NLRP3
106
β-actin
42
Astrocytes

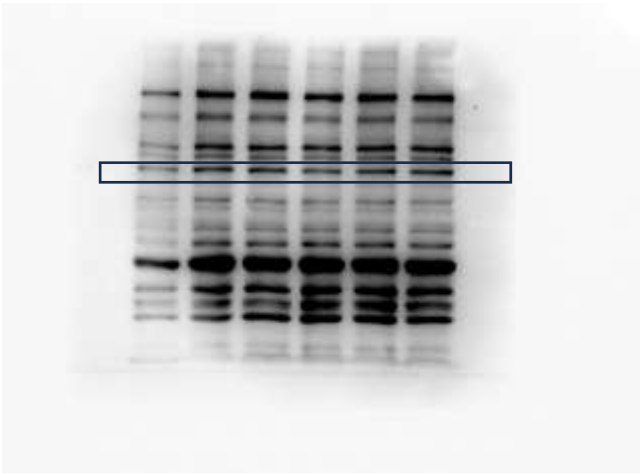
NLRP3

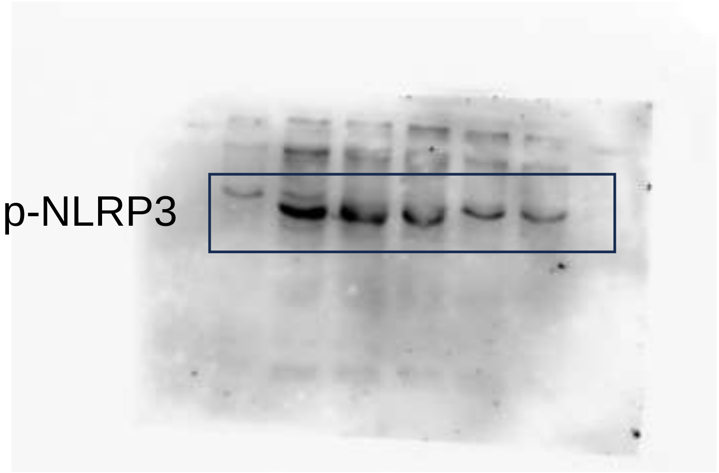
p-NLRP3

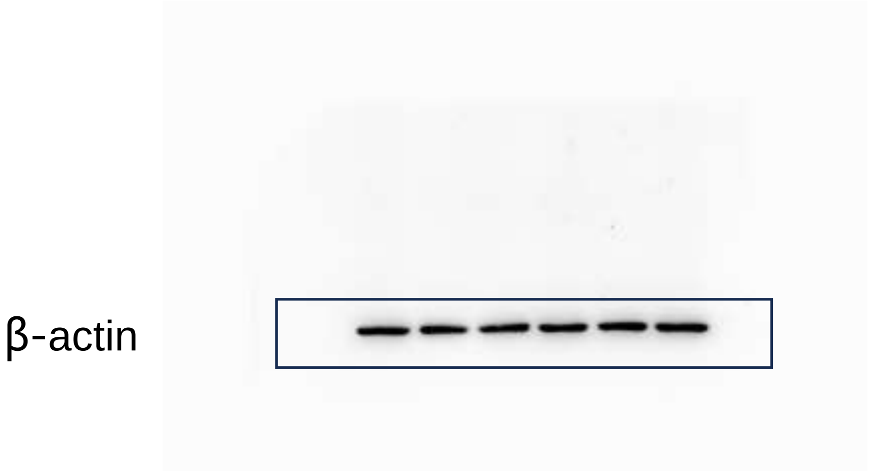
β-actin

FIG4 F

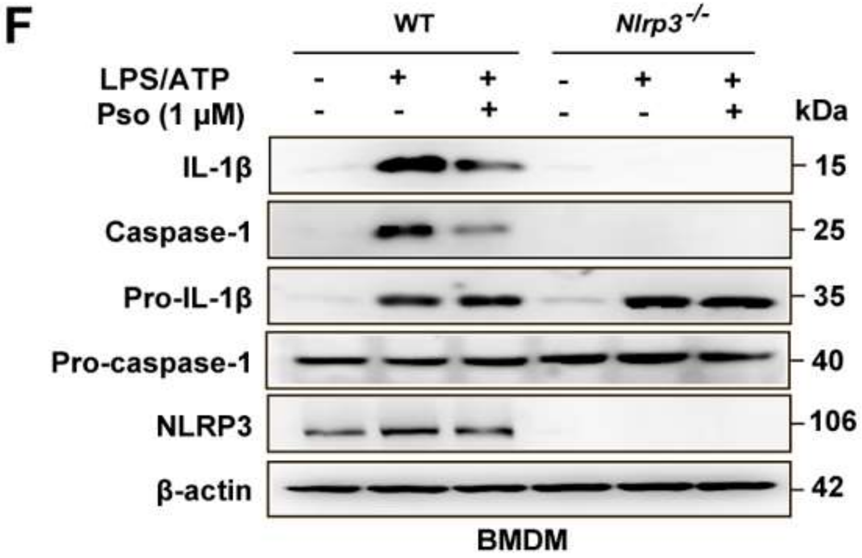

IL-1β

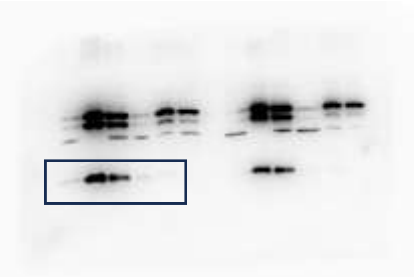

Caspase-1

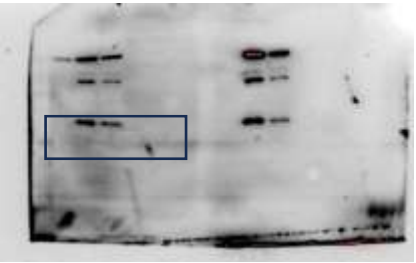

Pro-IL-1β

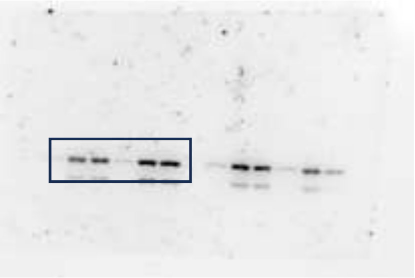

Pro-caspase-1

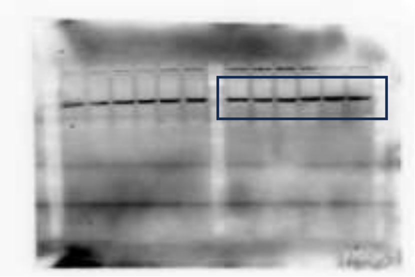

NLRP3

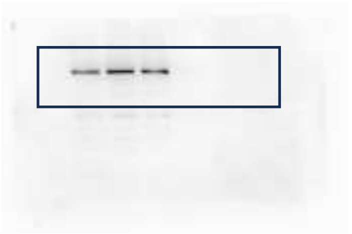

β-actin

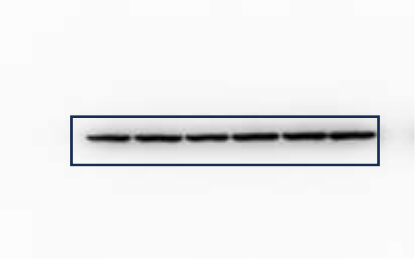

FIG 5C

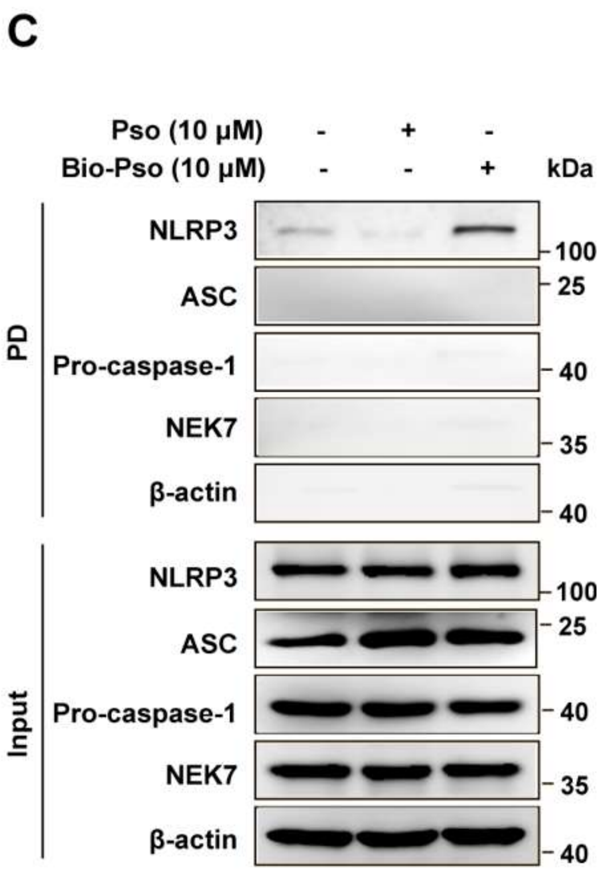

NLRP3-pull down

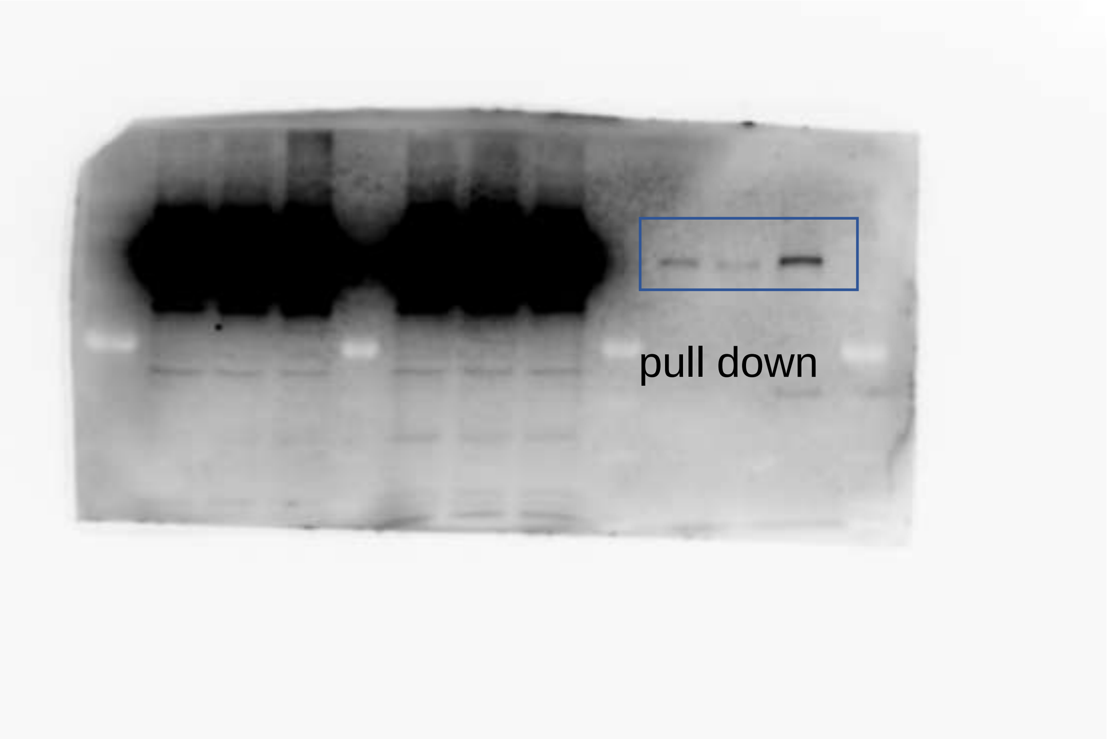

NLPR3-input

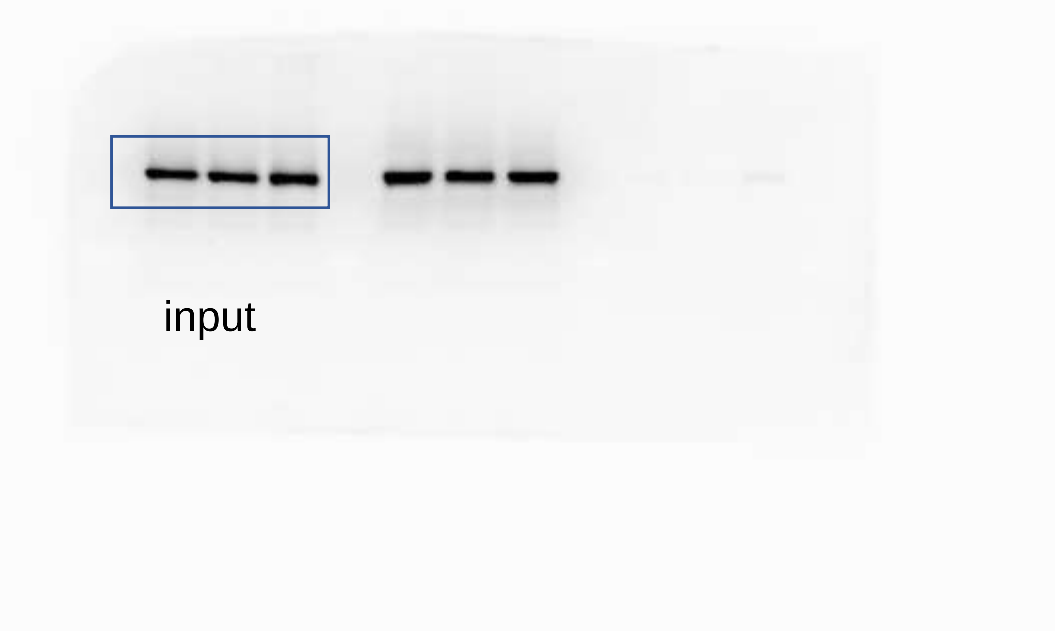

FIG 5C

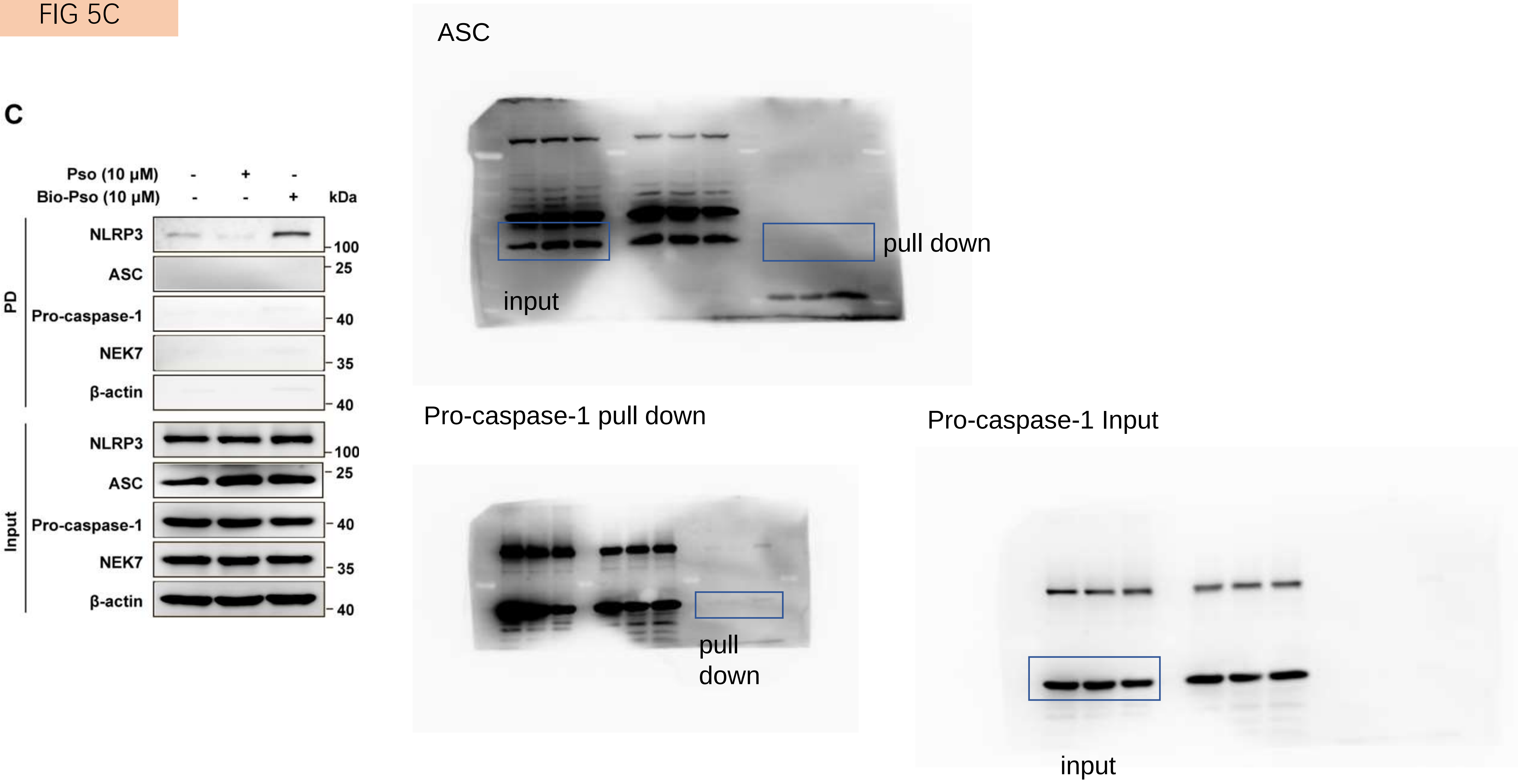
C
Pso (10 μM) - + -
Bio-Pso (10 μM) - - +
kDa
PD
NLRP3
ASC
Pro-caspase-1
NEK7
β-actin
Input
NLRP3
ASC
Pro-caspase-1
NEK7
β-actin
100
25
40
35
40
100
25
40
35
40
ASC
input
pull down
Pro-caspase-1 pull down
pull down
Pro-caspase-1 Input
input

FIG 5C

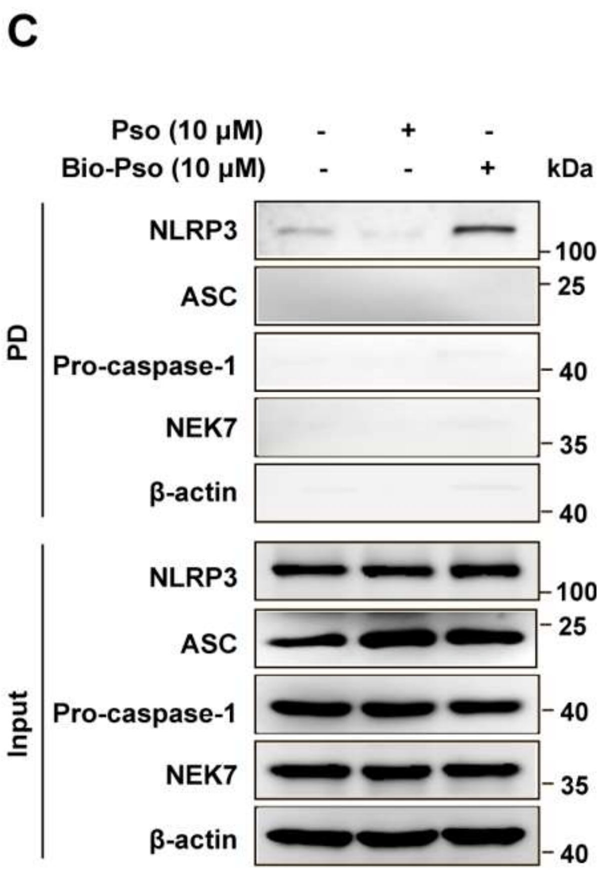

NEK7-pull down

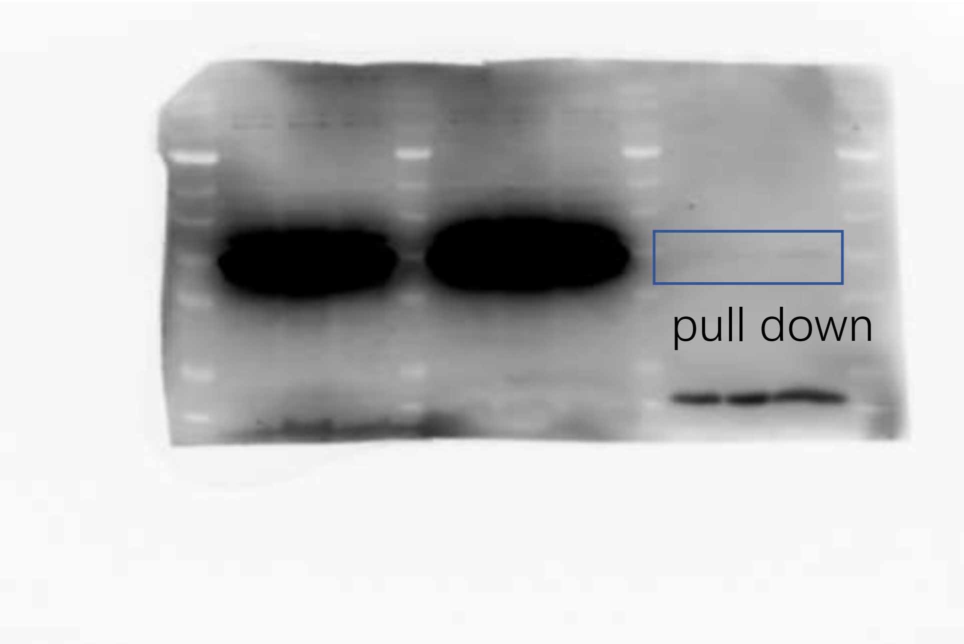

β-actin

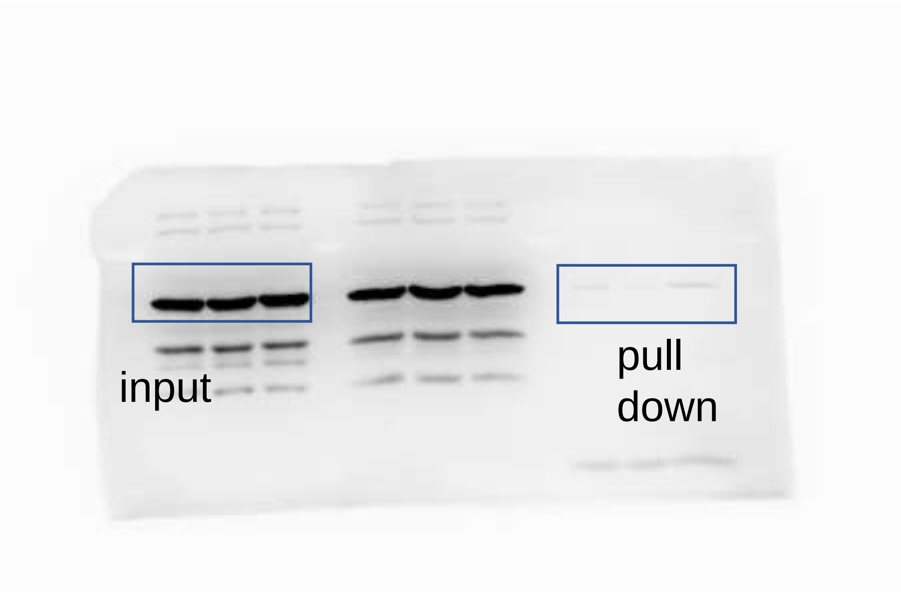

NEK7-input

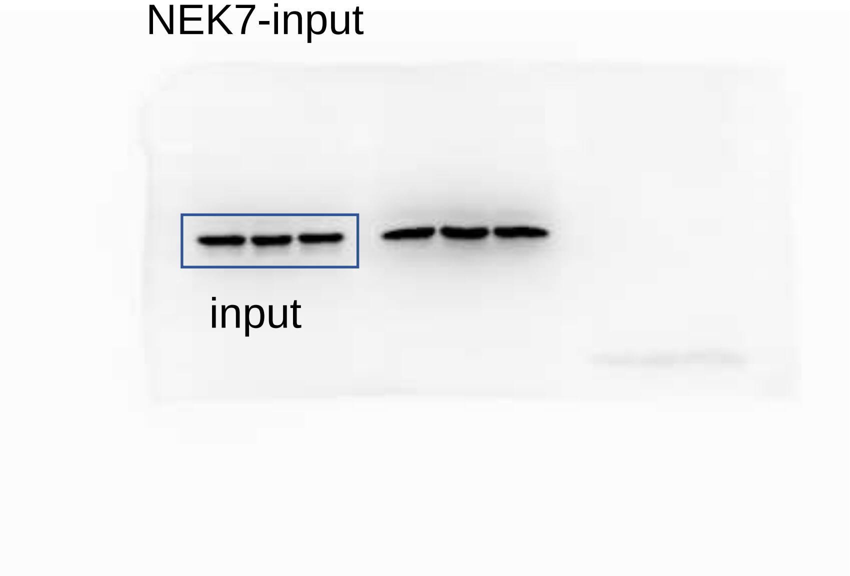

FIG5 D

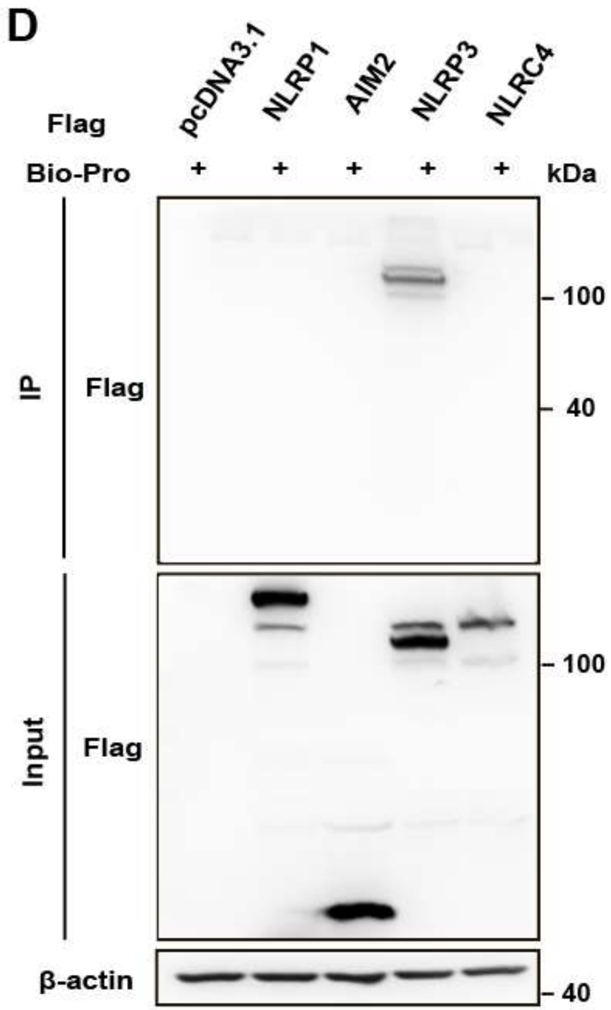

input

IP

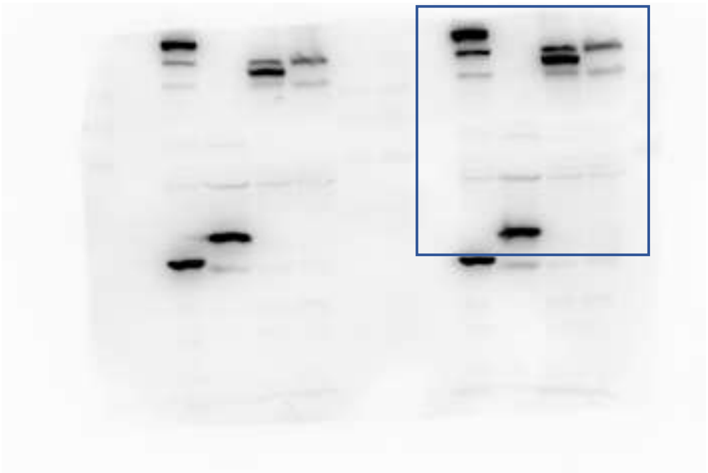

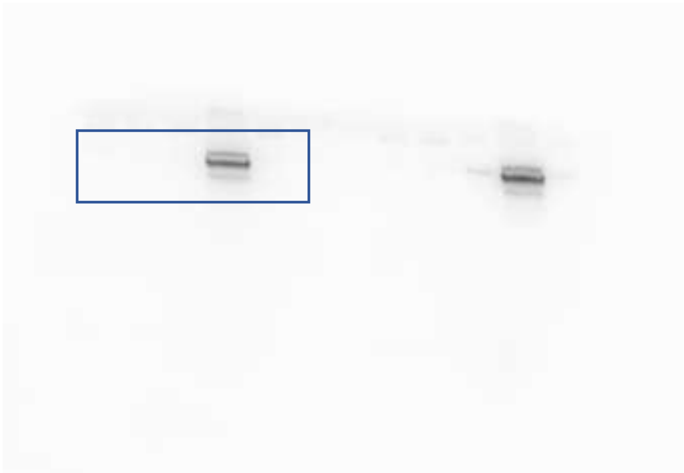

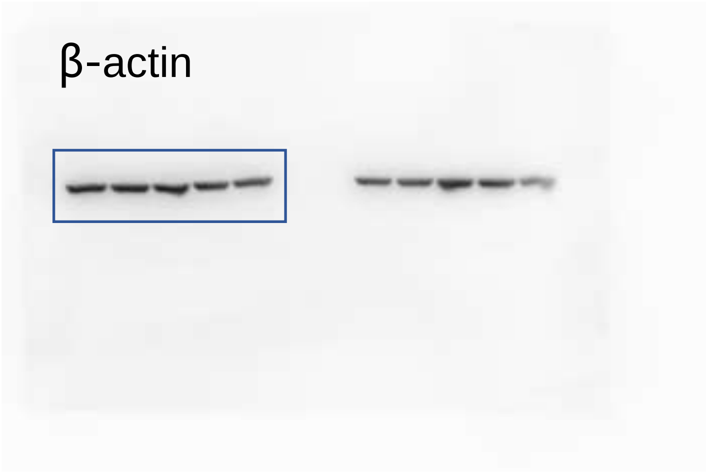

FIG5 E

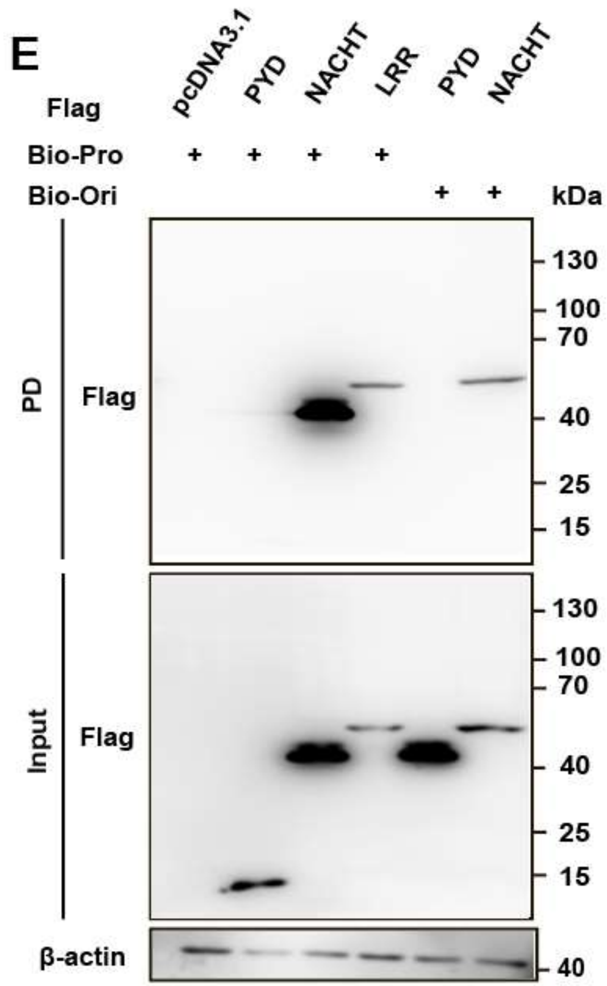

Pull down

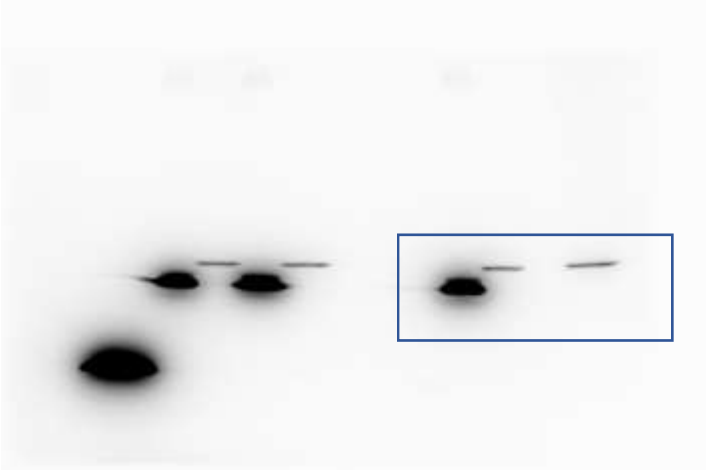

Input

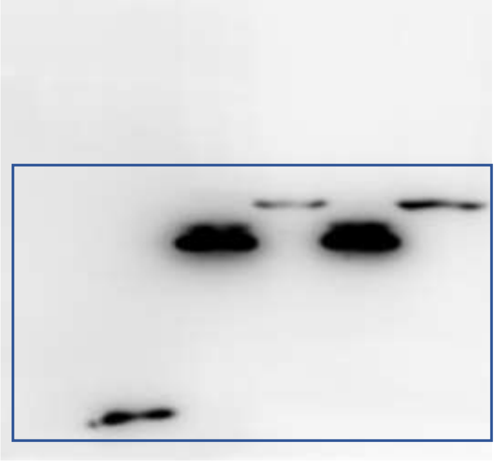

β-actin

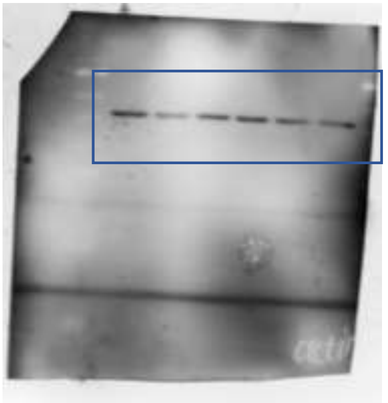

FIG6 G

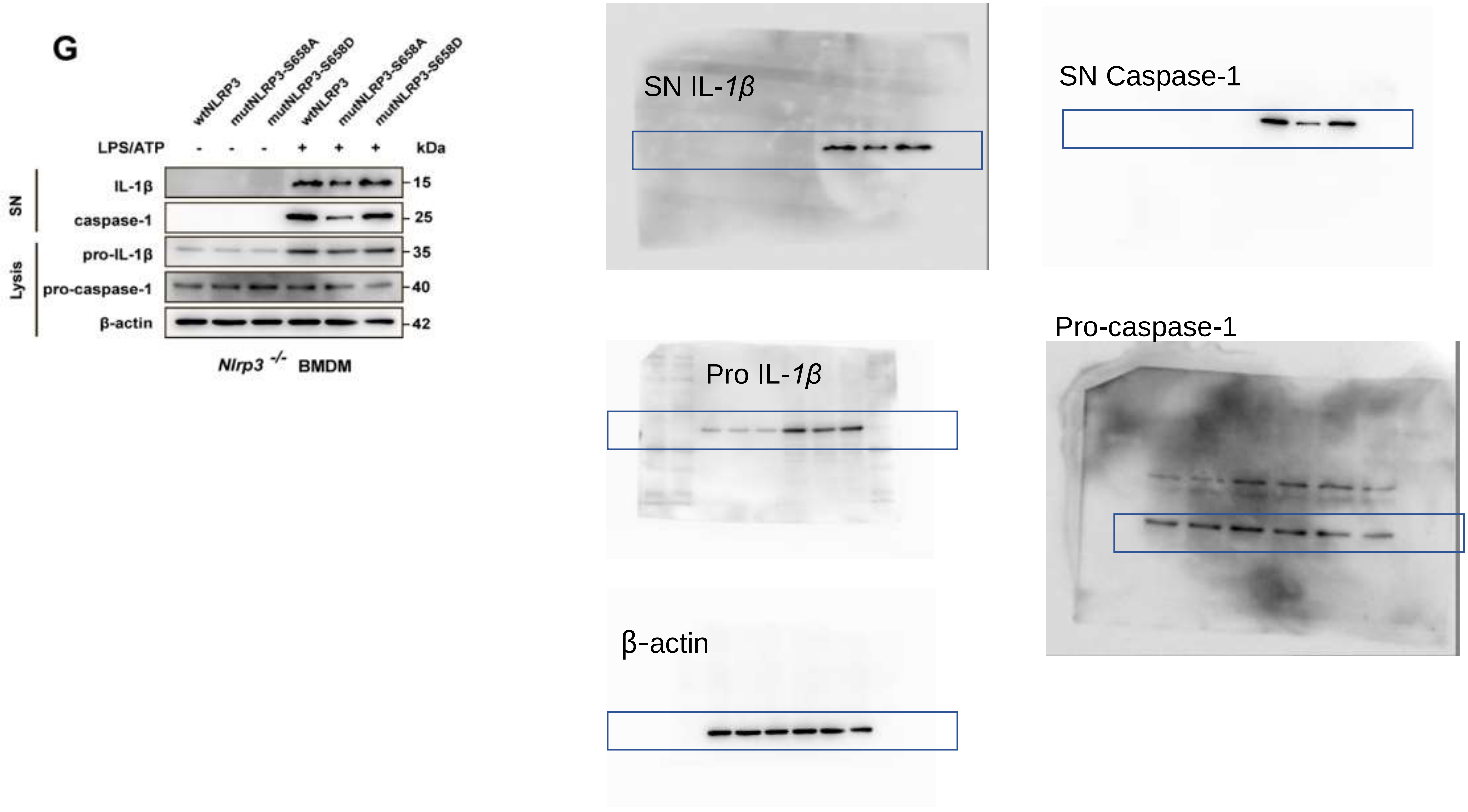
G
wtNLRP3
mutNLRP3-S658A
mutNLRP3-S658D
wtNLRP3
mutNLRP3-S658A
mutNLRP3-S658D
LPS/ATP - - - + + +
kDa
SN
IL-1β
15
caspase-1
25
Lysis
pro-IL-1β
35
pro-caspase-1
40
β-actin
42
Nlrp3 -/- BMDM
SN IL-1β
SN Caspase-1
Pro IL-1β
Pro-caspase-1
β-actin

FIG6 H

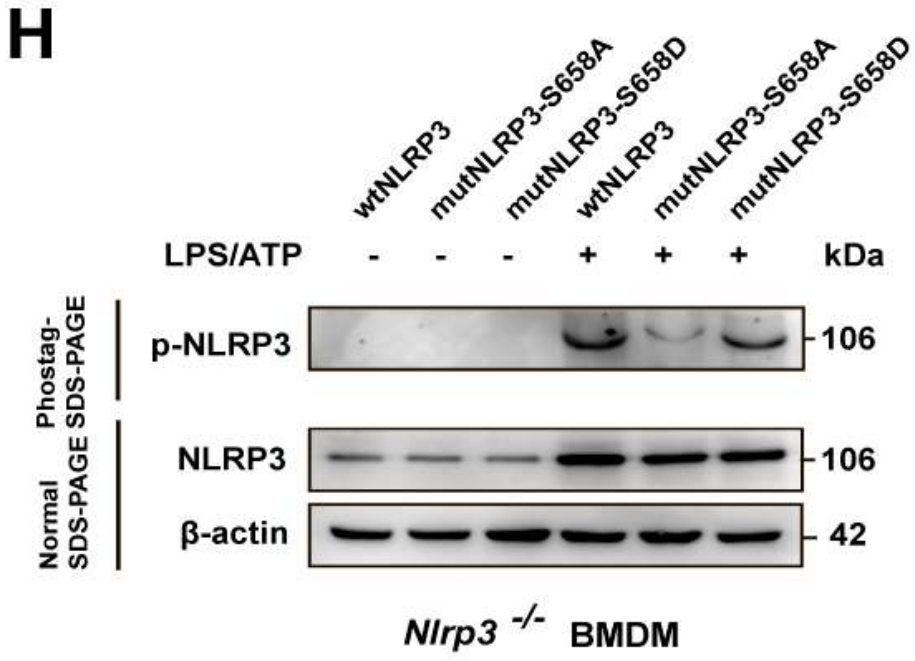
H
wtNLRP3
mutNLRP3-S658A
mutNLRP3-S658D
wtNLRP3
mutNLRP3-S658A
mutNLRP3-S658D
LPS/ATP - - - + + +
kDa
Phostag-SDS-PAGE
p-NLRP3
106
Normal SDS-PAGE
NLRP3
106
β-actin
42
Nlrp3 -/- BMDM

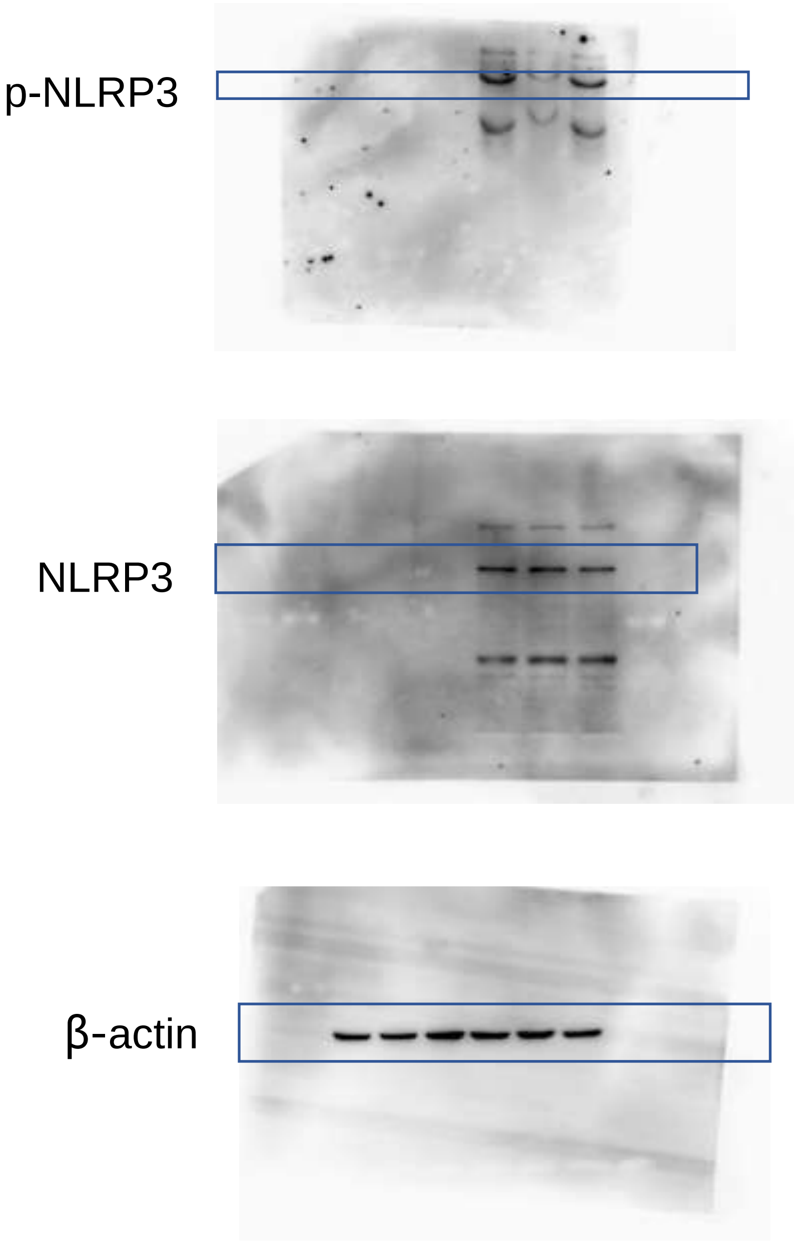
p-NLRP3
NLRP3
β-actin

FIG7 D

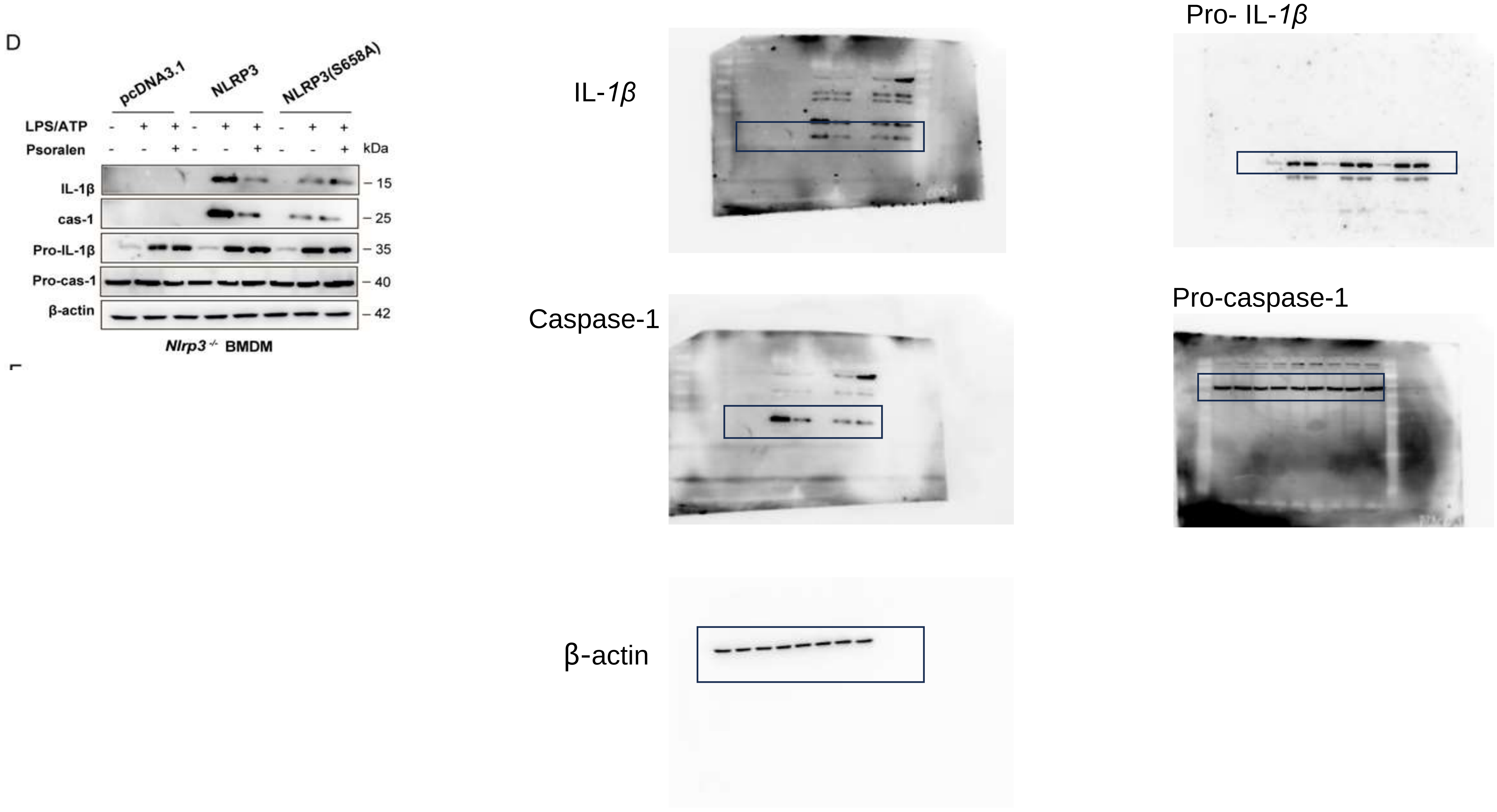
D
pcDNA3.1
NLRP3
NLRP3(S658A)
LPS/ATP
Psoralen
kDa
IL-1β
cas-1
Pro-IL-1β
Pro-cas-1
β-actin
15
25
35
40
42
$Nlrp3^{-/-}$ BMDM
IL-1β
Caspase-1
β-actin
Pro- IL-1β
Pro-caspase-1

FIG7 E

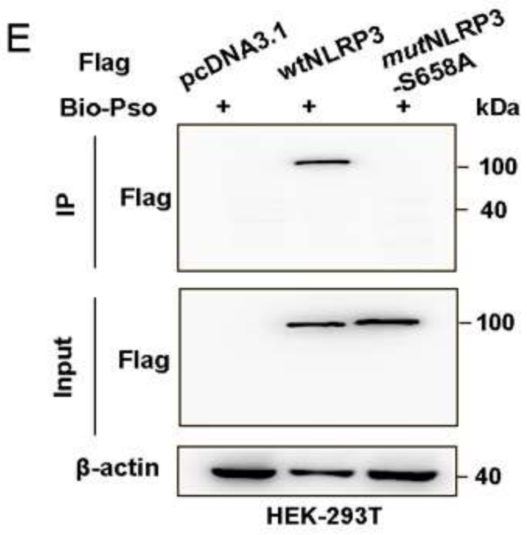
E
Flag
pcDNA3.1
wtNLRP3
mutNLRP3
-S658A
Bio-Pso
+
+
+
kDa
IP
Flag
100
40
Input
Flag
100
β-actin
40
HEK-293T

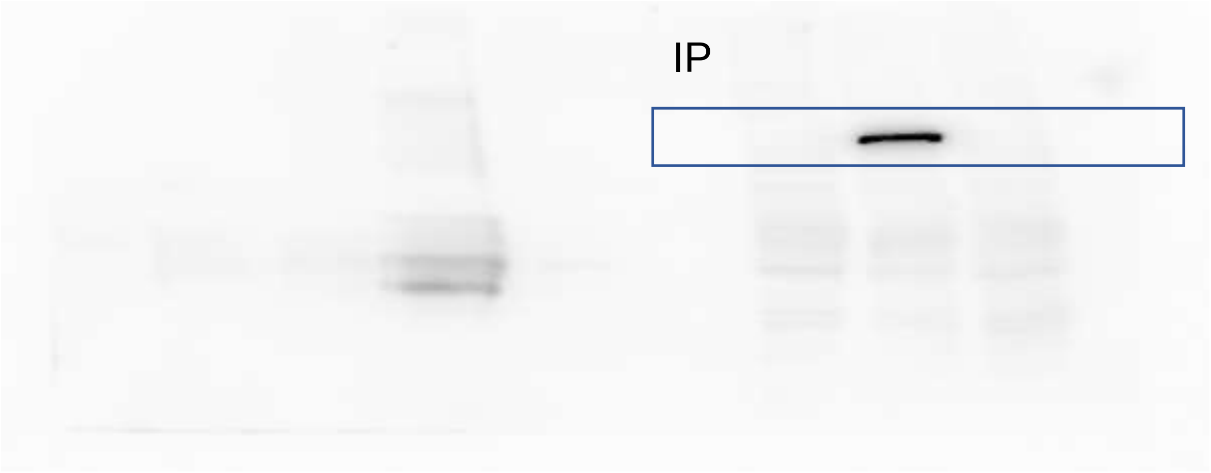
IP

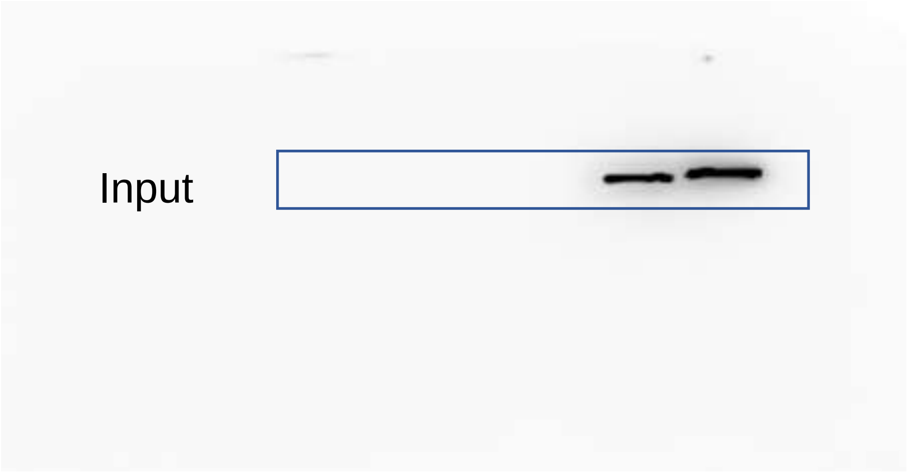
Input

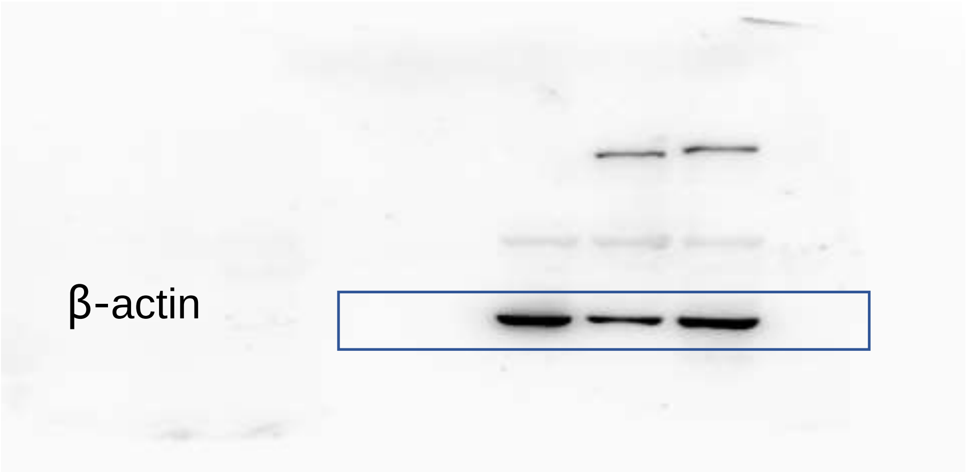
β-actin

FIG7 F

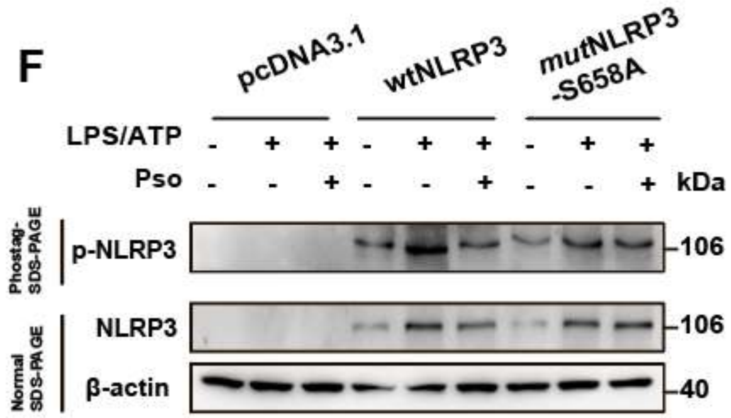

p-NLRP3

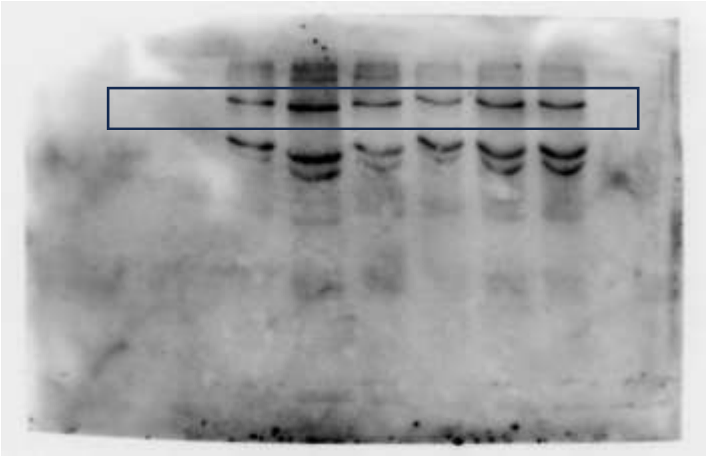

NLRP3

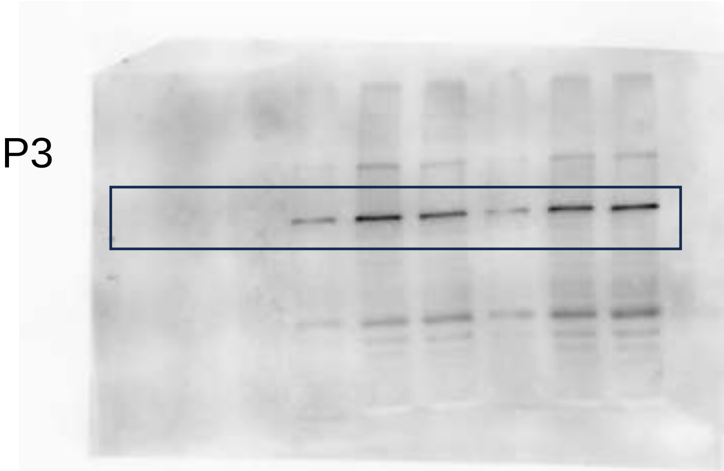

ACTIN

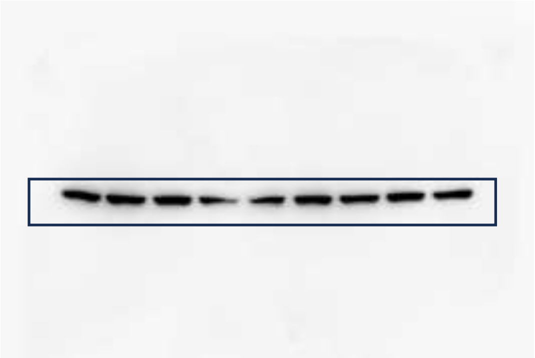

FIG8 P

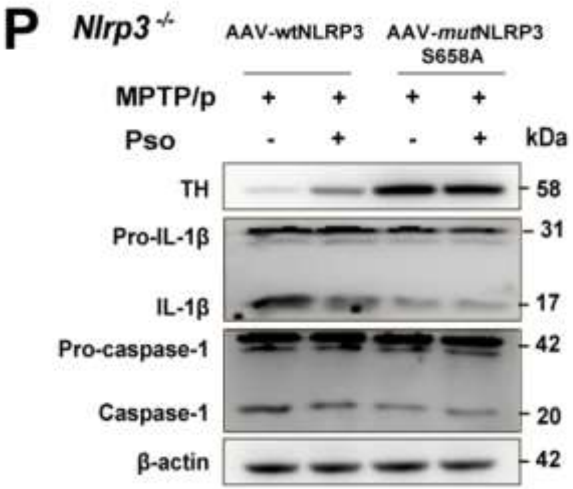

TH

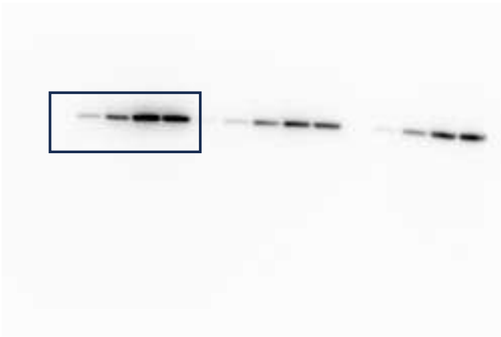

IL-1β

Pro- IL-1β

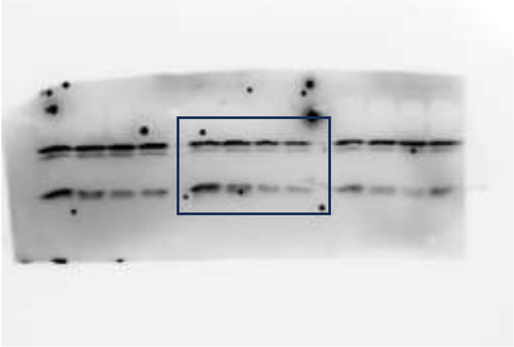

β-actin

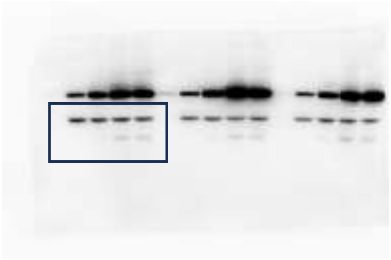

Caspase-1

Pro-caspase-1

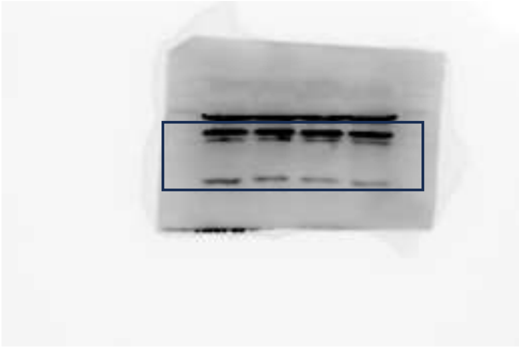

FIGS 10M

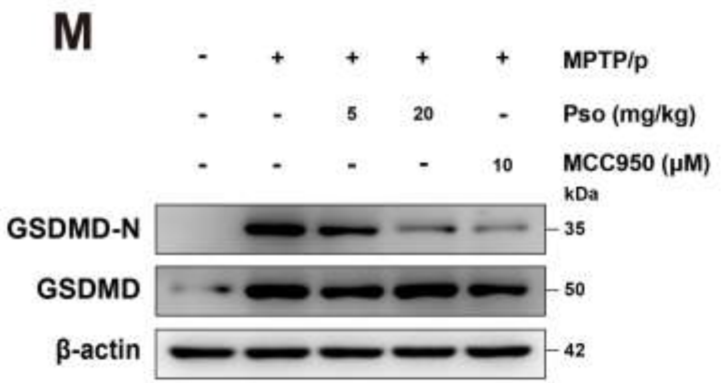

GSDMD

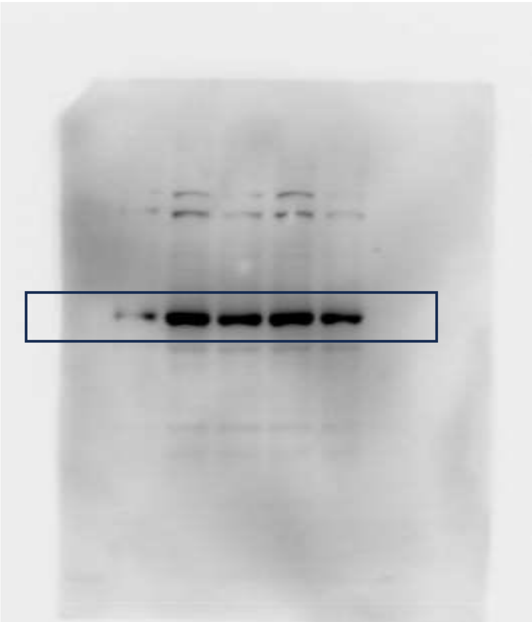

GSDMD-N

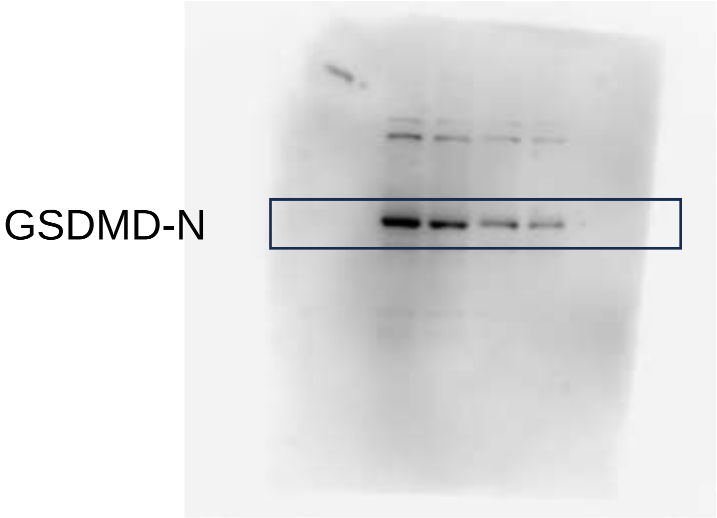

β-actin

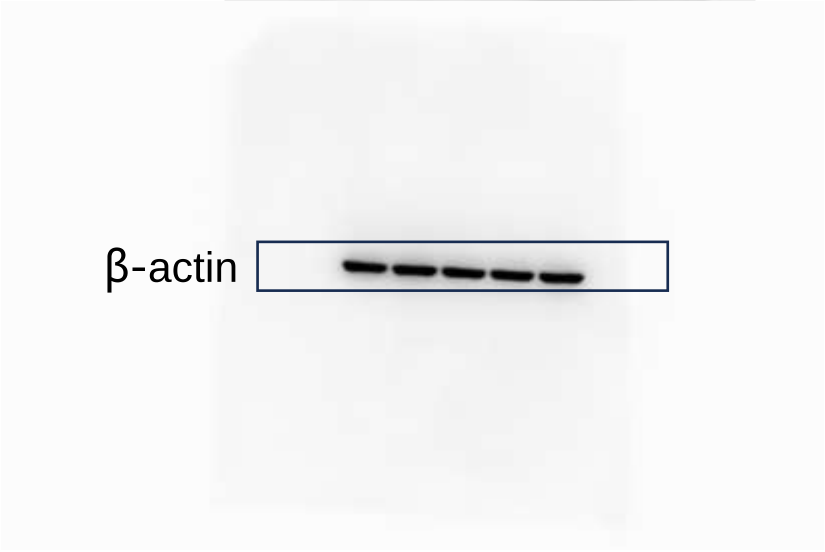

FIGS12 A

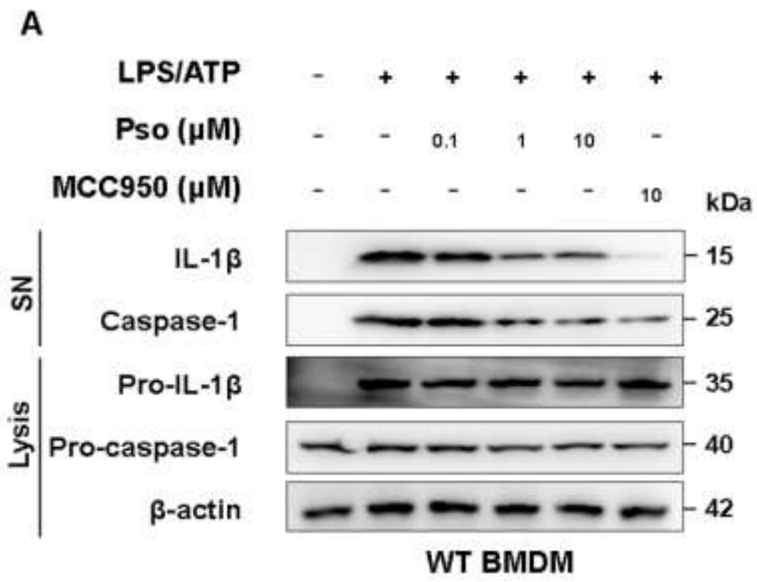

IL-*1β*

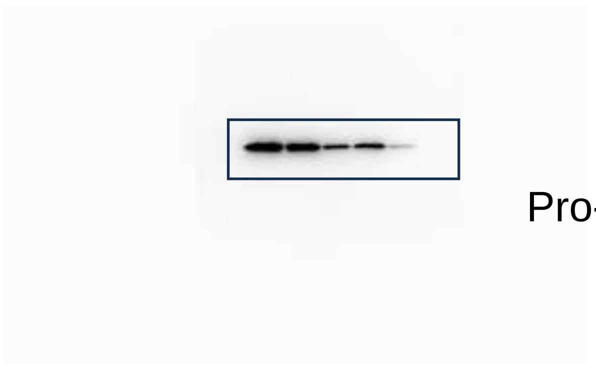

Pro- IL-*1β*

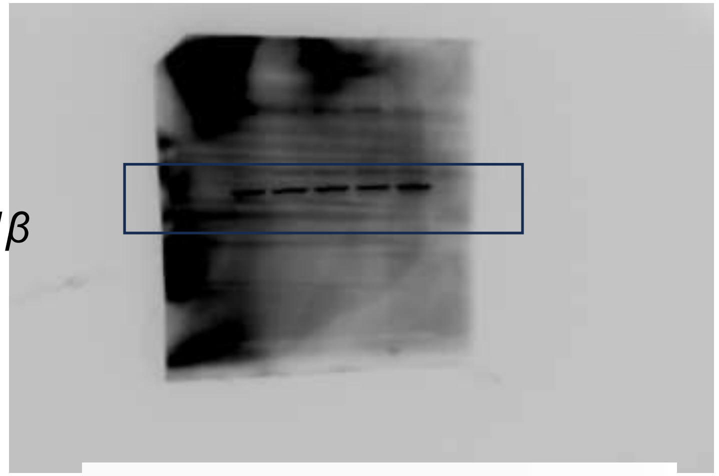

Caspase-1

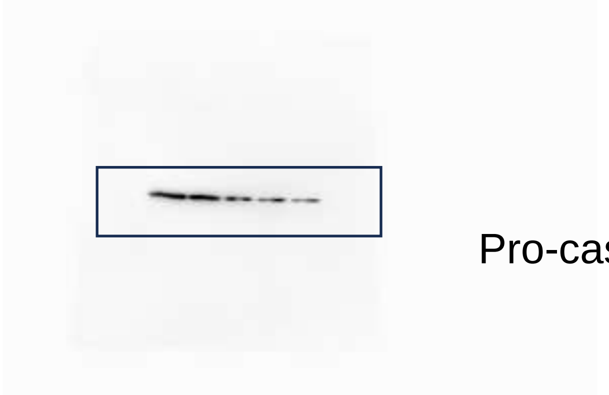

Pro-caspase-1

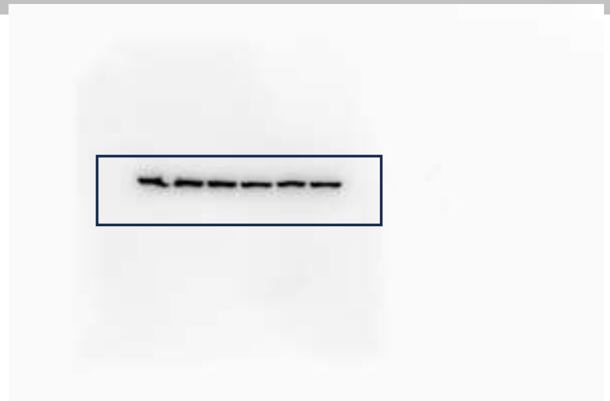

β-actin

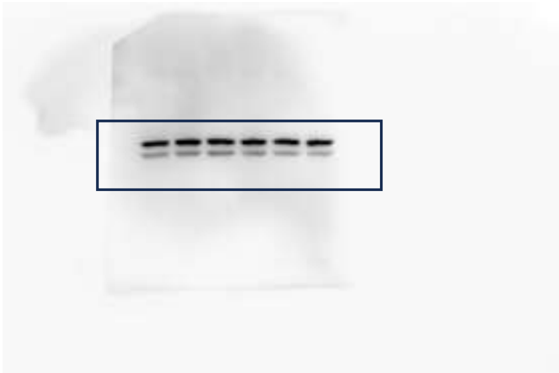

FIGS12 C

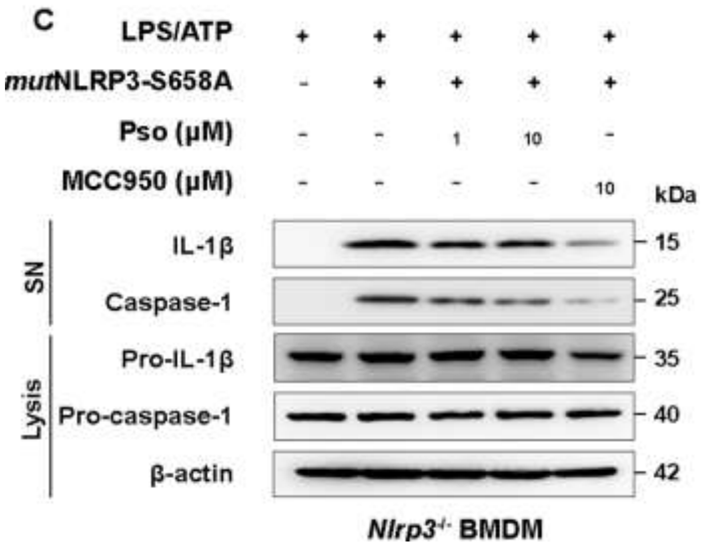

IL-1β

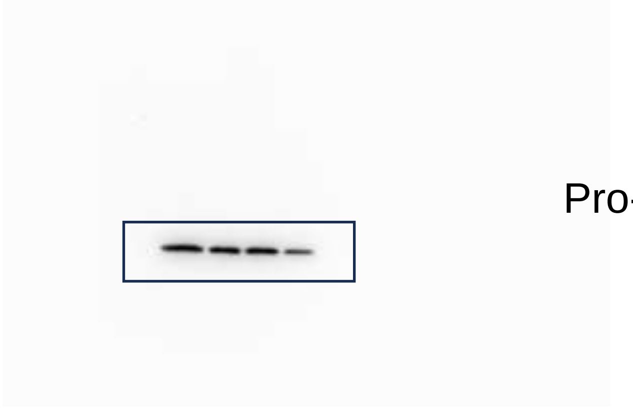

Pro- IL-1β

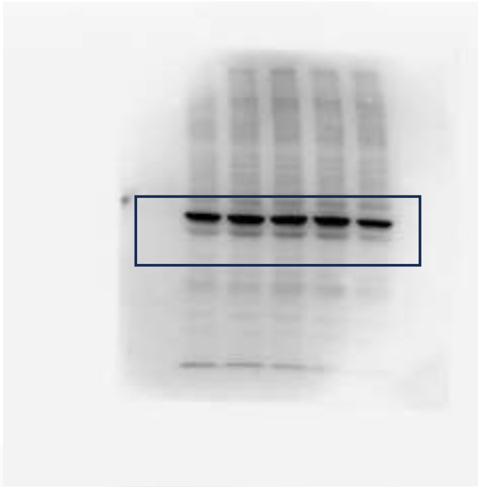

Caspase-1

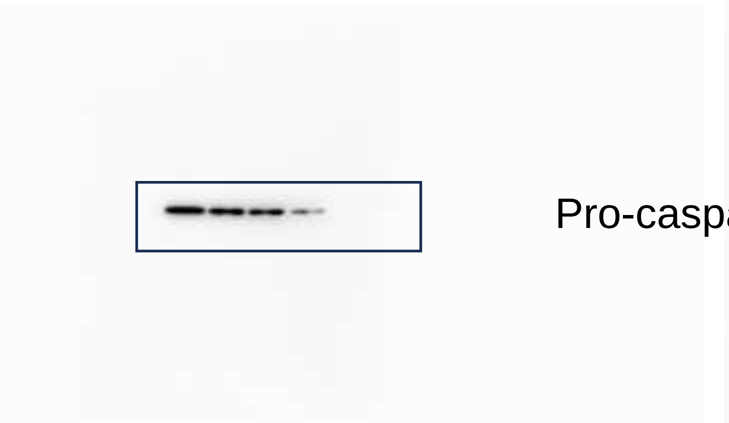

Pro-caspase-1

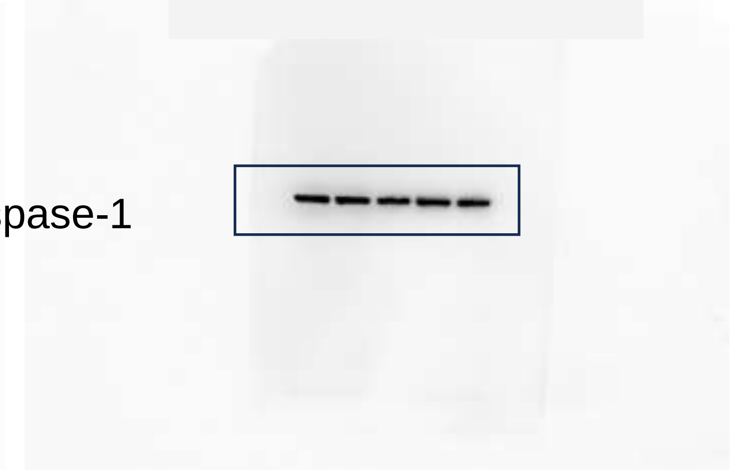

β-actin

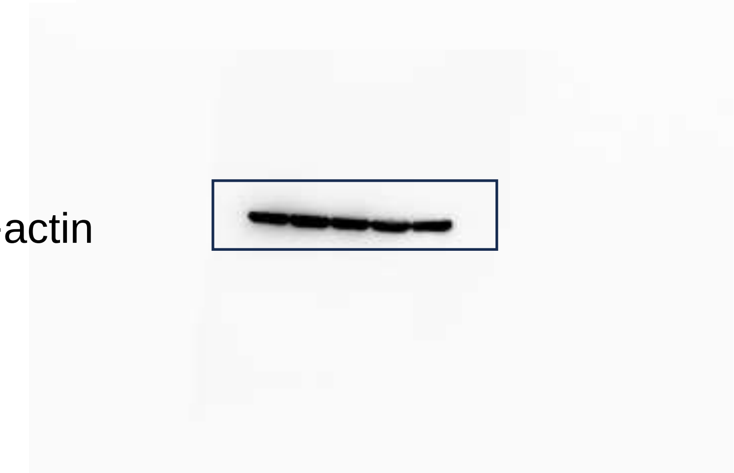

FIGS13 C

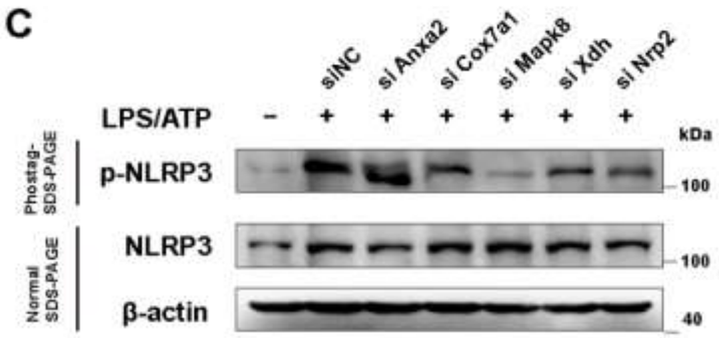

p-NLRP3

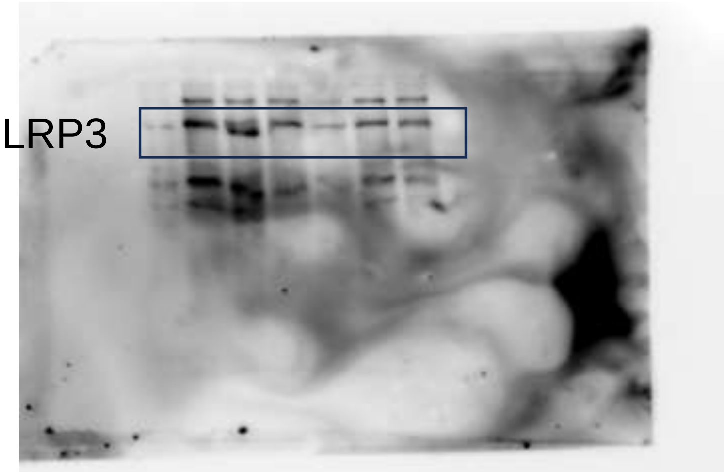

NLRP3

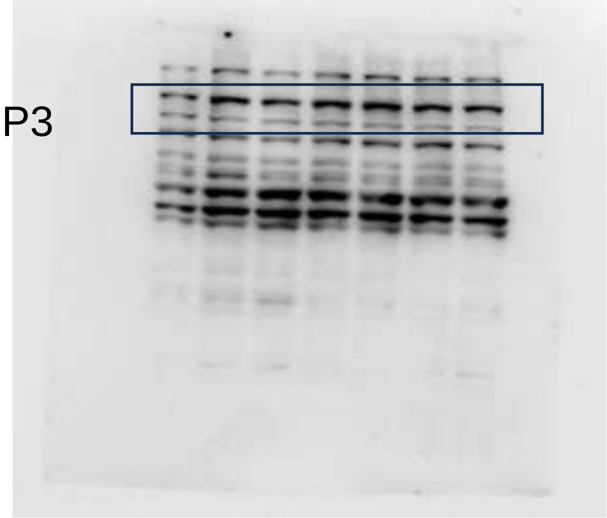

β-actin

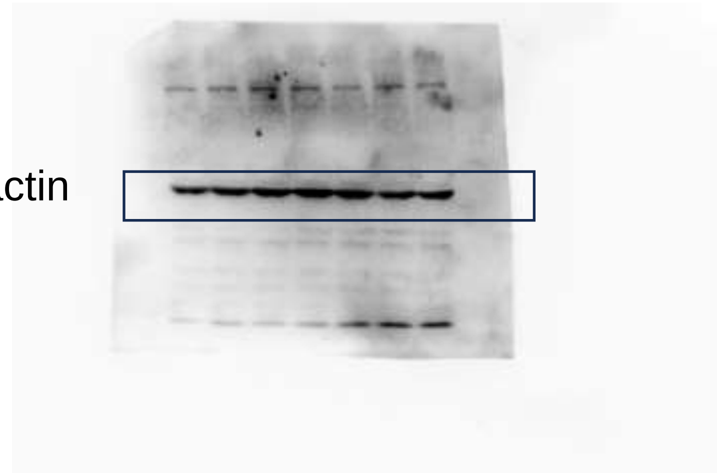

FIGS13 D

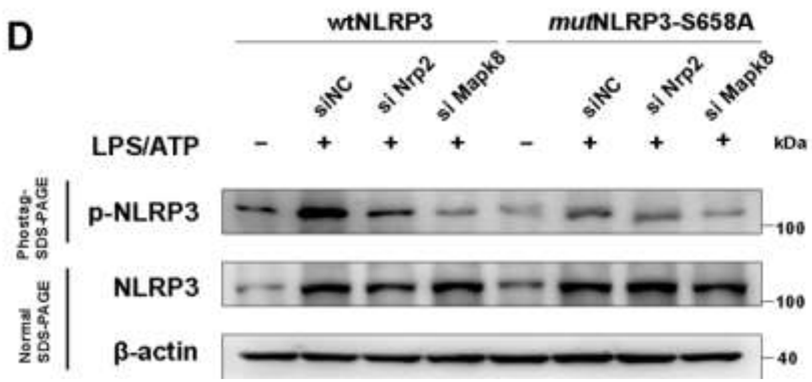

p-NLRP3

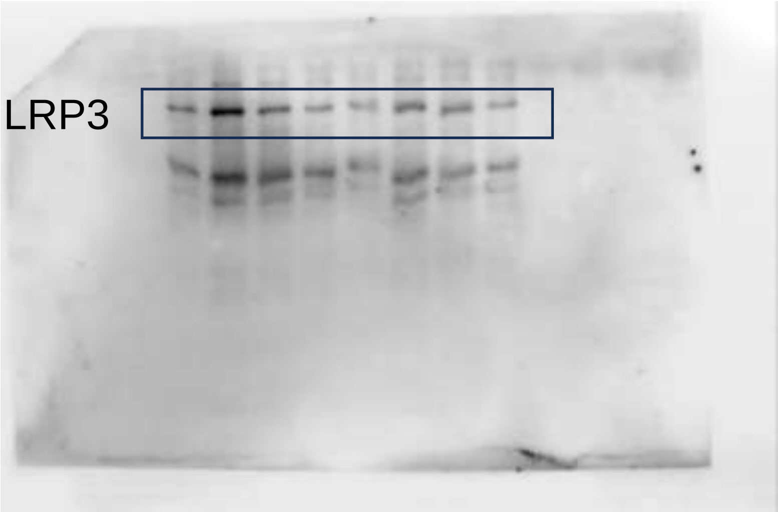

NLRP3

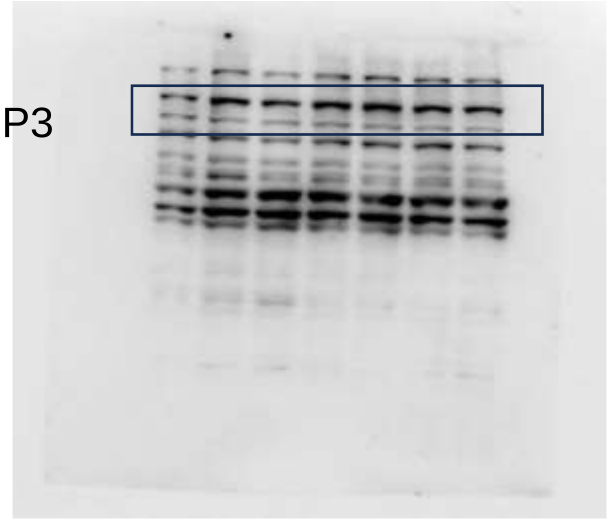

β-actin

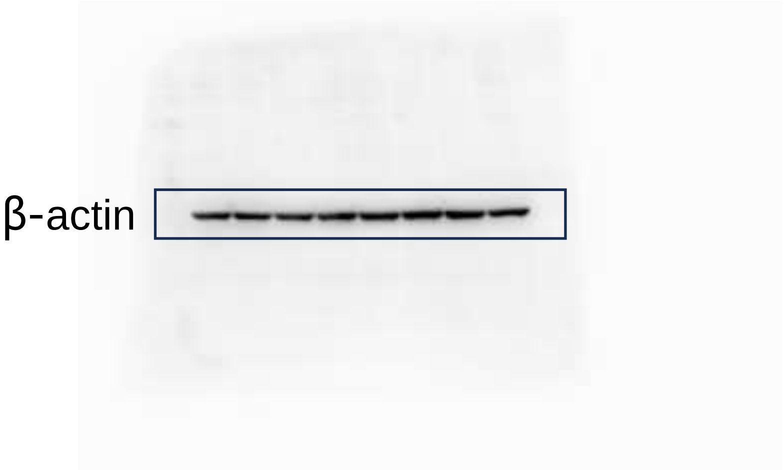

Supplement: Supplementary file 1 — Supplementary Material 1 [file 13024_2025_818_MOESM1_ESM.pdf]
