# Supplementary material for: Inactivation of NLRP3 inflammasome by dephosphorylation at Serine 658 alleviates glial inflammation in the mouse model of Parkinson’s disease
Source: Mol Neurodegener. 2025 Mar 5;20:27. doi: 10.1186/s13024-025-00818-z (PMC11881452; doi:10.1186/s13024-025-00818-z)
Supplement: Supplementary file 2 — Supplementary Material 2 [file 13024_2025_818_MOESM2_ESM.docx]

**Additional File 1**

**Inactivation of NLRP3 inflammasome by dephosphorylation at Serine 658 alleviates glial inflammation in the mouse model of Parkinson’s disease**

Rong-Xin Zhu ^a1^, Rui-Xue Han^a1^, Yue-Han Chen ^a^, Lei Huang ^a^,Ting Liu^a^, Jingwei Jiang^c^, Cong Wang^a^, Lei Cao^a*^, Yang Liu ^b*^, Ming Lu^a*^

*^a^ Jiangsu Key Laboratory of Neurodegeneration, Department of Pharmacology, Nanjing Medical University, Nanjing, 211116, China.*

*^b^ Department of Pharmacology, Nanjing University of Chinese Medicine, Nanjing, Jiangsu, 210023, China.*

*^c^ China Pharmaceutical University, Nanjing, 211116, China.*

^*^**Correspondence**

Ming Lu: lum@njmu.edu.cn

Yang Liu: liuyang@njucm.edu.cn

Lei Cao: leicao@njmu.edu.cn

**^1^ These authors contributed equally to this work.**

**Supplementary Figures**


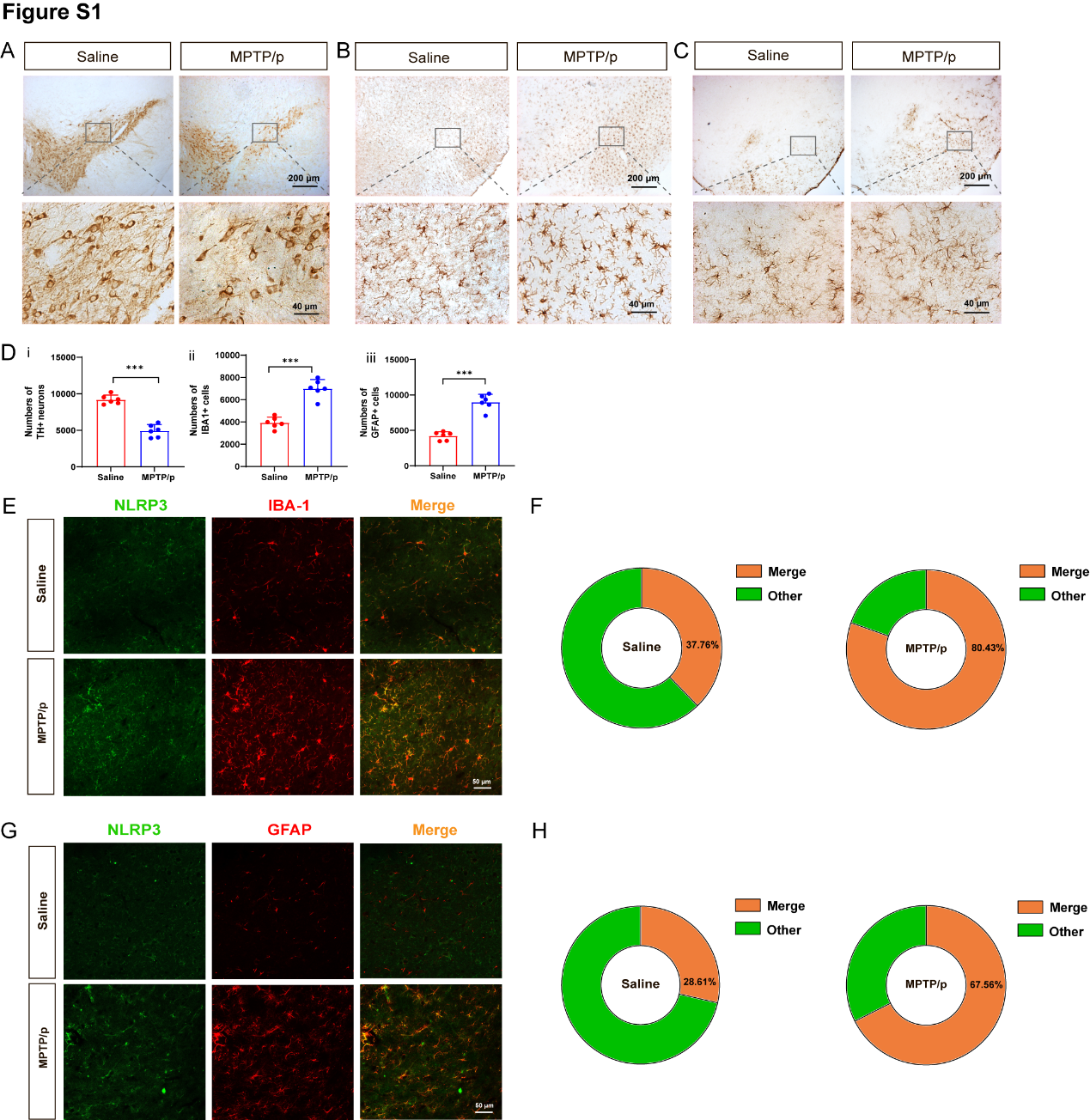
**Figure S1****. Loss of DA neurons is accompanied by heightened reactivity and NLRP3 expression in microglia and astrocytes of MPTP/p-treated mice.**

Representative images of immunohistochemical staining of TH **(A)**, IBA-1 **(B)**, and GFAP **(C)** in the SNpc. The scale bar represents 200μm. Enlarge vision: 40μm. **(D)** Quantification of TH^+^ DA neurons, IBA-1^+^ microglia, and GFAP^+^ astrocytes in the SNc. Data were analyzed by Student’s t-test. ***P < 0.001. **(E-F)** Immunostaining and quantification of NLRP3 (green) and microglia marker IBA1(red) in the SNc. **(G-H)** Immunostaining and quantification for NLRP3 (green) and astrocyte marker GFAP (red) in the SNc. The scale bar represents 50μm.


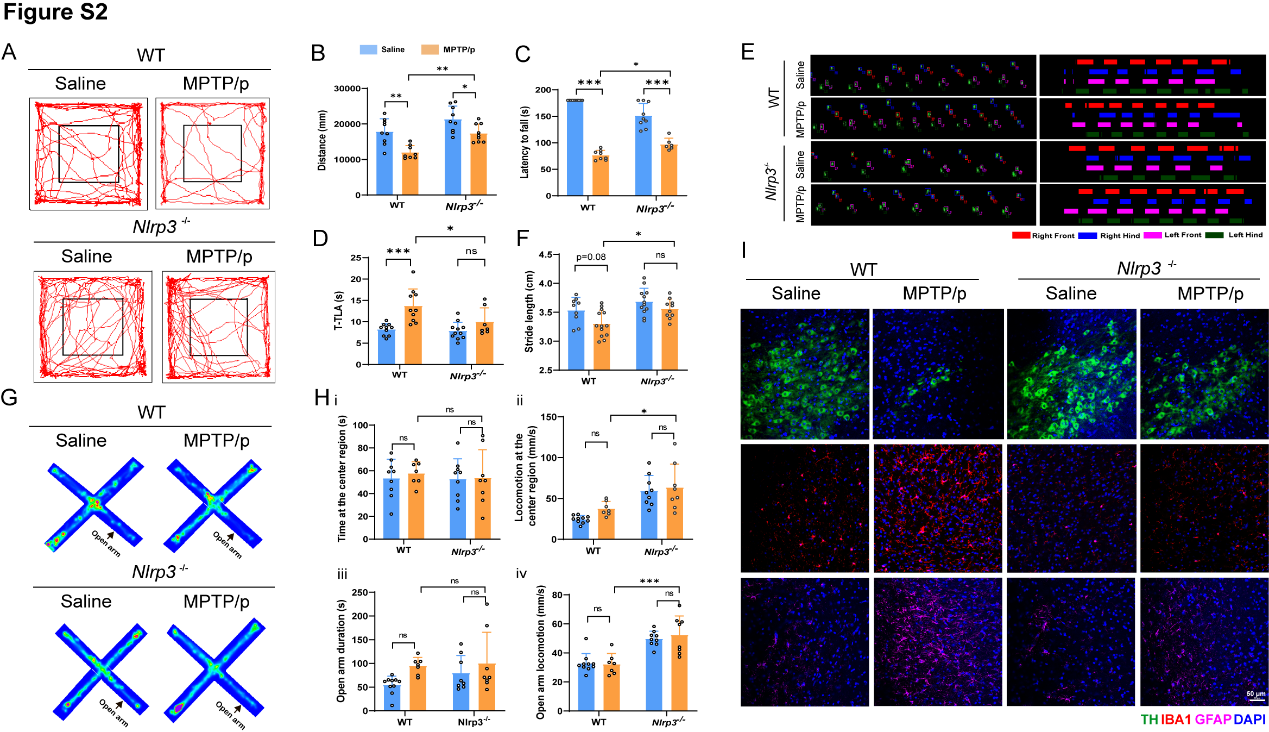


**Figure S2. NLRP3 knockout abolishes the motor dysfunction and neurodegeneration in the MPTP/p mouse model of PD.**

**(A-B)** The travel path and movement distance were recorded in the open field test. **(C)** The latency to fall was measured in the rotarod test. **(D)** The time taken to descend a pole (T-TLA) was recorded in the pole test, n=6-10. **(E)** Gait trace of the WT and *Nlrp3* KO mice in the Elevated plus maze test. **(F)** Stride length was analyzed by WalkAnalysisator software. **(G)** Movement trace of the WT and *Nlrp3* KO mice in the Elevated plus maze test (the arrow indicates the open arm). **(H)** Statistical analyses of the time at the center region (i), locomotion at the center region (ii), the duration in the open arm (iii), and the open arm locomotion (iv) in the Elevated plus maze test. **(I)** Immunostaining for TH (green), IBA1 (red), and GFAP (pink) in the SNc of MPTP/p-treated WT and *Nlrp3* KO mice. DAPI stains the nucleus (blue). The scale bar represents 50μm. Data were analyzed by two-way ANOVA, followed by Tukey post-tests. *P < 0.05, **P < 0.01, and ***P < 0.001. ns: no significance.


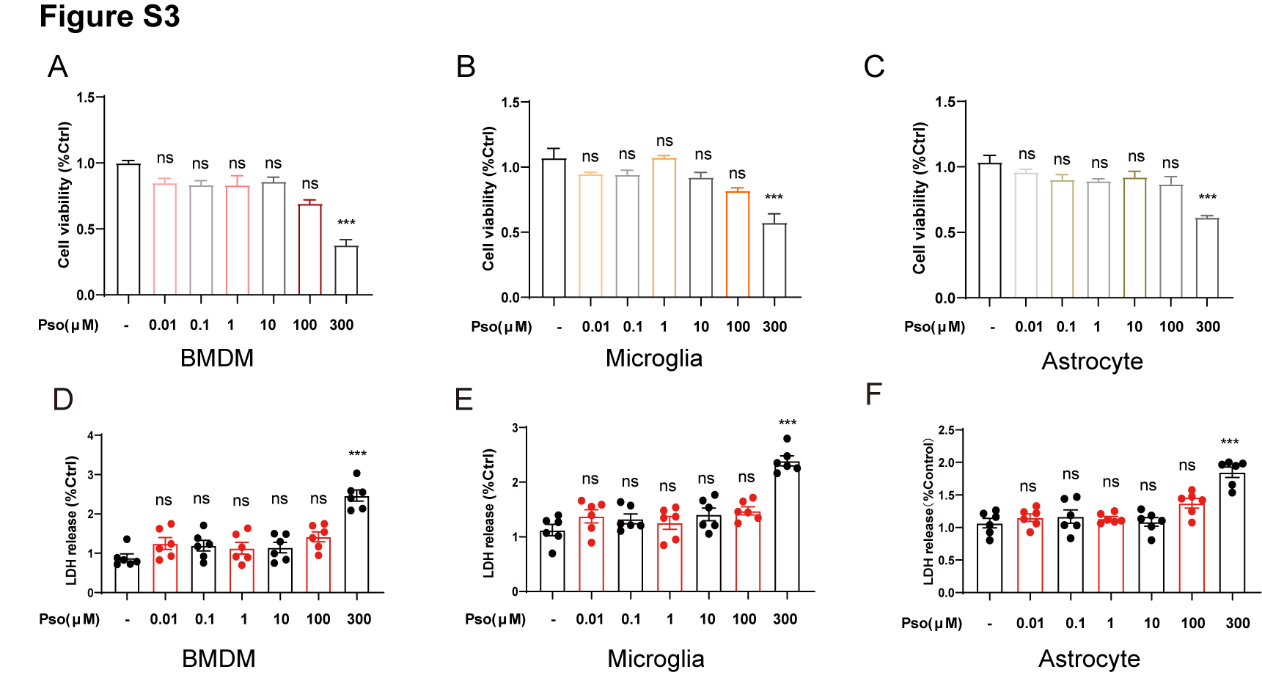


**Figure S3. Effects of different concentrations of** **Psoralen on cell viability and LDH release of BMDMs, microglia, and astrocytes.**

Relative cell viability and LDH release were detected in BMDMs **(A, D)**, microglia **(B, E)**, and astrocytes **(C, F)** treated with various concentrations of Psoralen (0.01, 0.1, 1, 10, 100, and 300 μM). Data were analyzed by one-way ANOVA, followed by Tukey post-tests. ***P < 0.001 vs control group. ns: no significance.


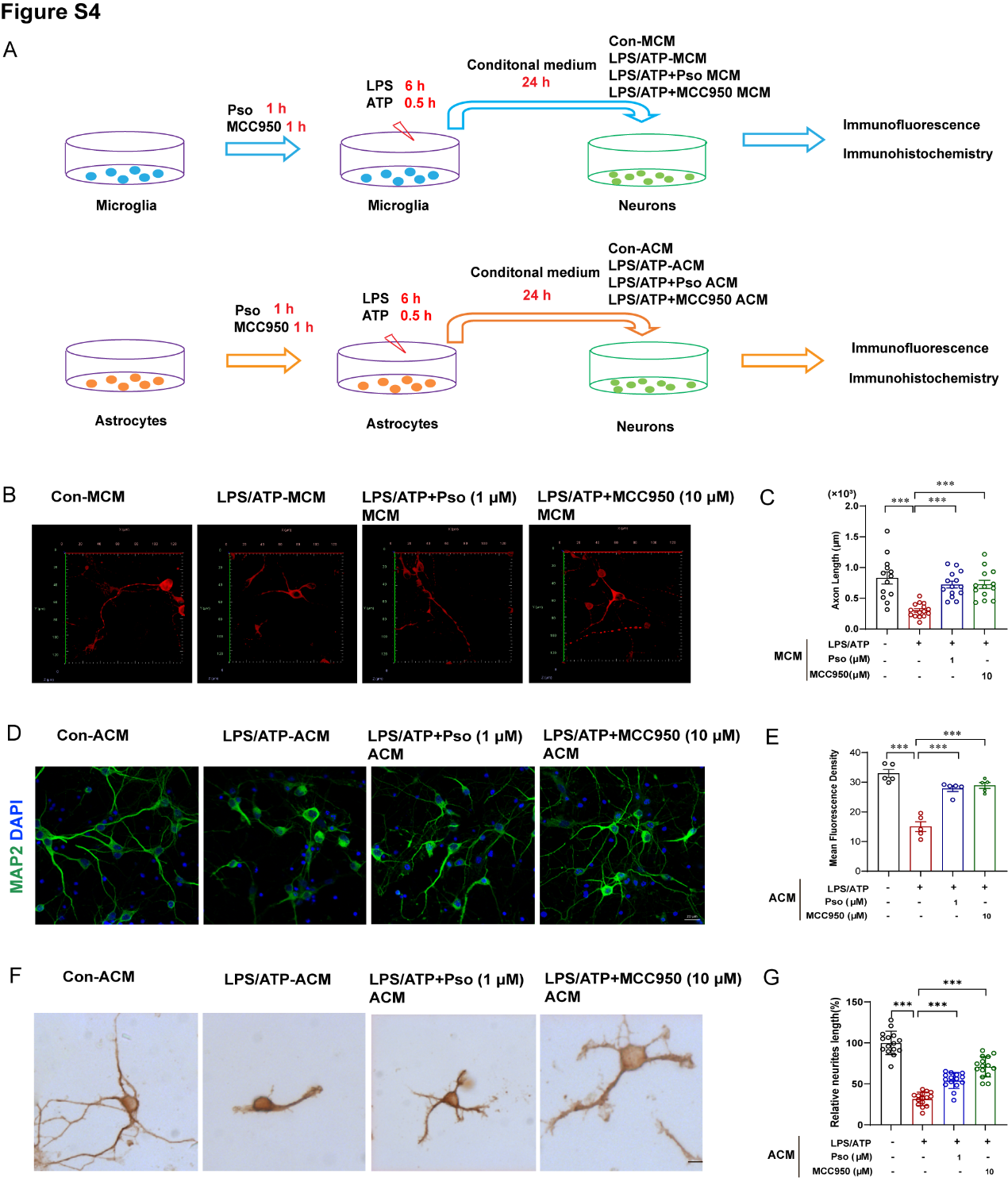


**Figure S4. Psoralen exerts neuroprotective effects via inhibiting NLRP3 inflammasome activation.**

**(A)** The schematic model of the indirect co-culture system of glia-neurons. **(B)** Immunofluorescence staining of TH to examine the effects of microglial conditioned medium on DA neuronal survival. The scale bar represents 20μm. **(C)** Quantification of axon length. **(D-G)** Immunofluorescence staining and quantification of MAP2 and TH to examine the effects of astrocytic conditioned medium on neuronal survival, n =15cells/group. Data were analyzed by one-way ANOVA, followed by Tukey post-tests. *P < 0.05, **P < 0.01, and ***P < 0.001.


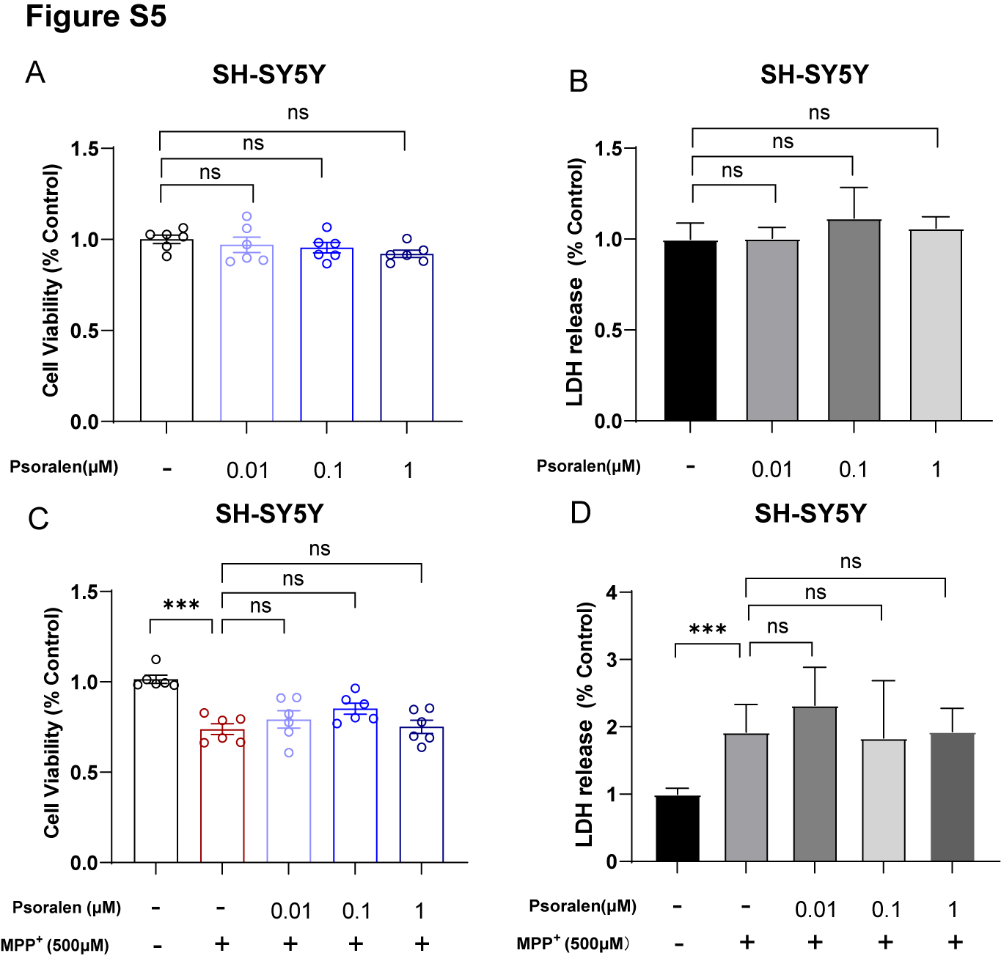


**Figure S5. Psoralen has no impact on the cell viability and LDH release in SH-SY5Y cells.**

**(A-B)** Effects of Psoralen on the cell viability and LDH release in SH-SY5Y cells at basal condition. **(C-D)** Effects of Psoralen on the cell viability and LDH release in SH-SY5Y cells in the MPP^+^ model. Data were analyzed by one-way ANOVA, followed by Tukey post-tests. ***P < 0.001. ns: no significance.


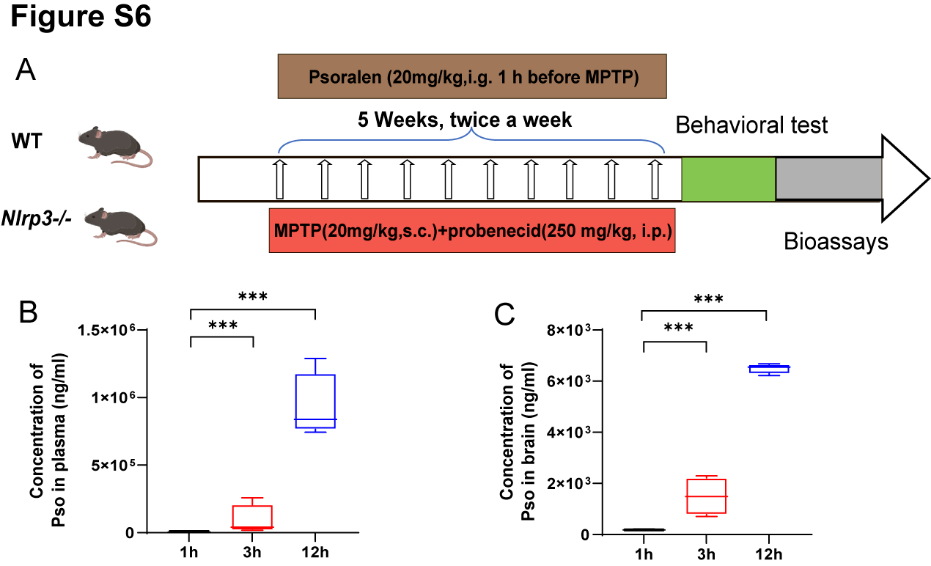


**Figure S6. Psoralen is able to cross the blood-brain barrier.**

**(A)** Schematic diagram of the experimental procedure in the MPTP/p mouse model. UPLC-MS/MS detected the concentration of Psoralen in the plasma **(B)** and brain **(C)**. Data were analyzed by one-way ANOVA, followed by Tukey post-tests. ***P < 0.001.


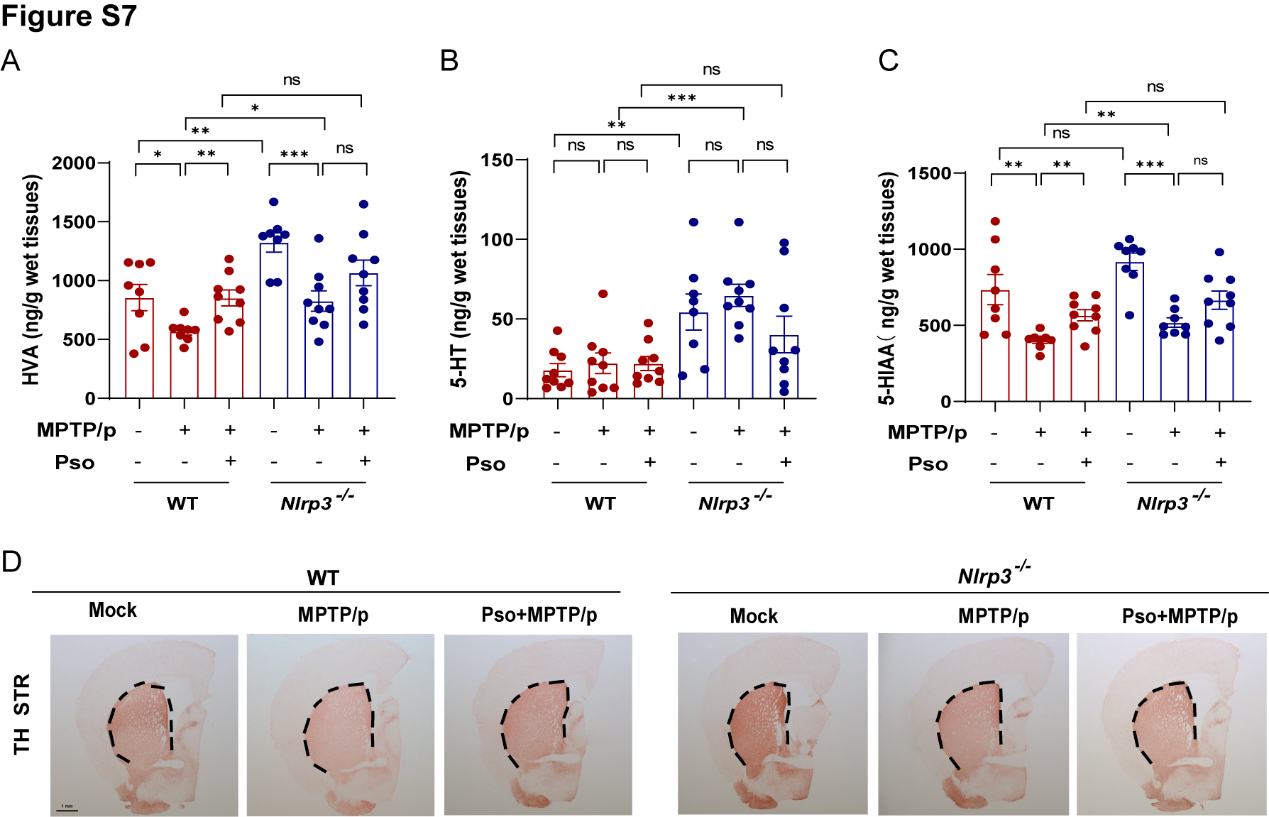


**Figure S7. NLRP3 knockout abolishes the neuroprotective effect of Psoralen on DA neurodegeneration.**

**(A-C)** The levels of HVA, 5-HT, and 5-HIAA in striatum homogenate was detected by HPLC, n=8-10. **(D)** Representative immunohistochemical staining of TH^+^ neurons in the striatum, n=6. Data were analyzed by one-way ANOVA, followed by Tukey post-tests. *P < 0.05, **P < 0.01, and ***P < 0.001. ns: no significance.


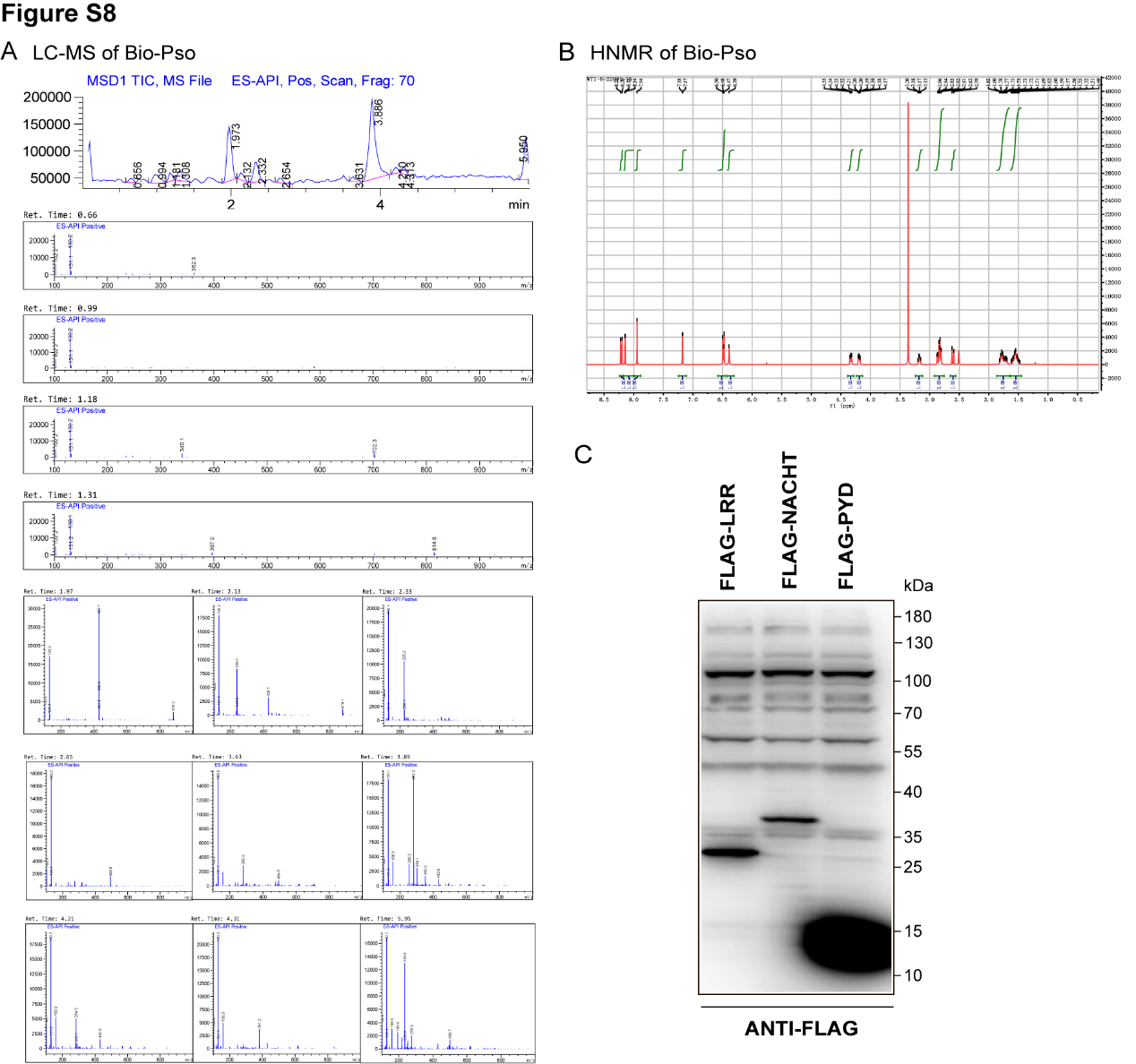


**Figure S8. Synthesis and validation of biotinylated Psoralen.**

**(A)** The LC-MS spectrogram of biotinylated Psoralen. **(B)** The HNMR spectrogram of biotinylated Psoralen. **(C)** Verification of transfection efficiency of LRR, NACHT, and PYD domain of NLRP3 protein.


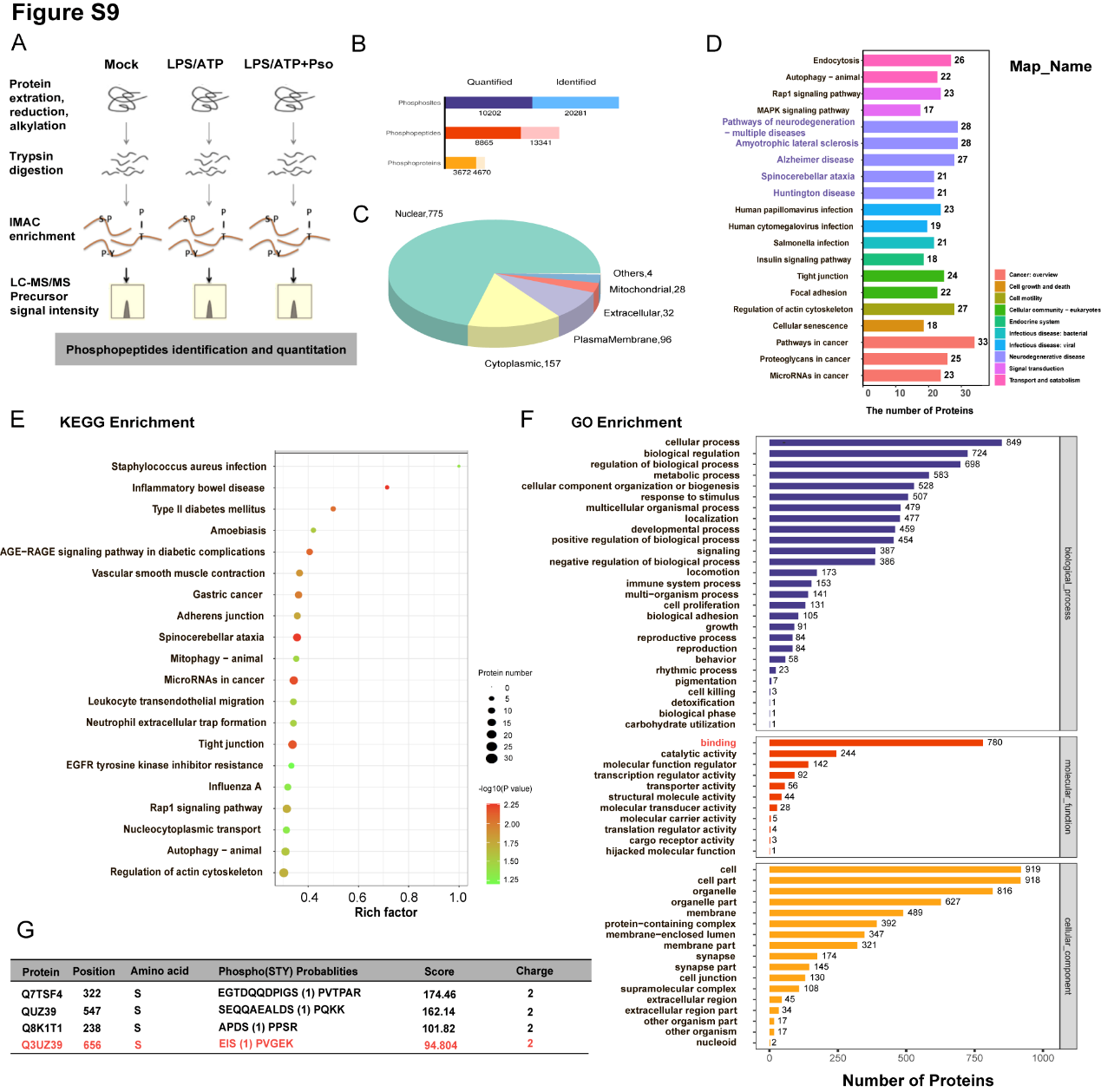


**Figure S9. Bioinformatics analysis of 4D label-free quantitative phosphorylation proteomics.**

**(A)** Schematic diagram of the experimental procedure of phosphorylation proteomics. **(B)** The quantified and identified phosphorylation peptides. **(C)** The cellular distribution of phosphorylation proteins. Signaling pathway mapping **(D)**, KEGG enrichment analysis **(E)**, and GO enrichment analysis **(F)**. **(G)** Phosphorylation sites with top scores.


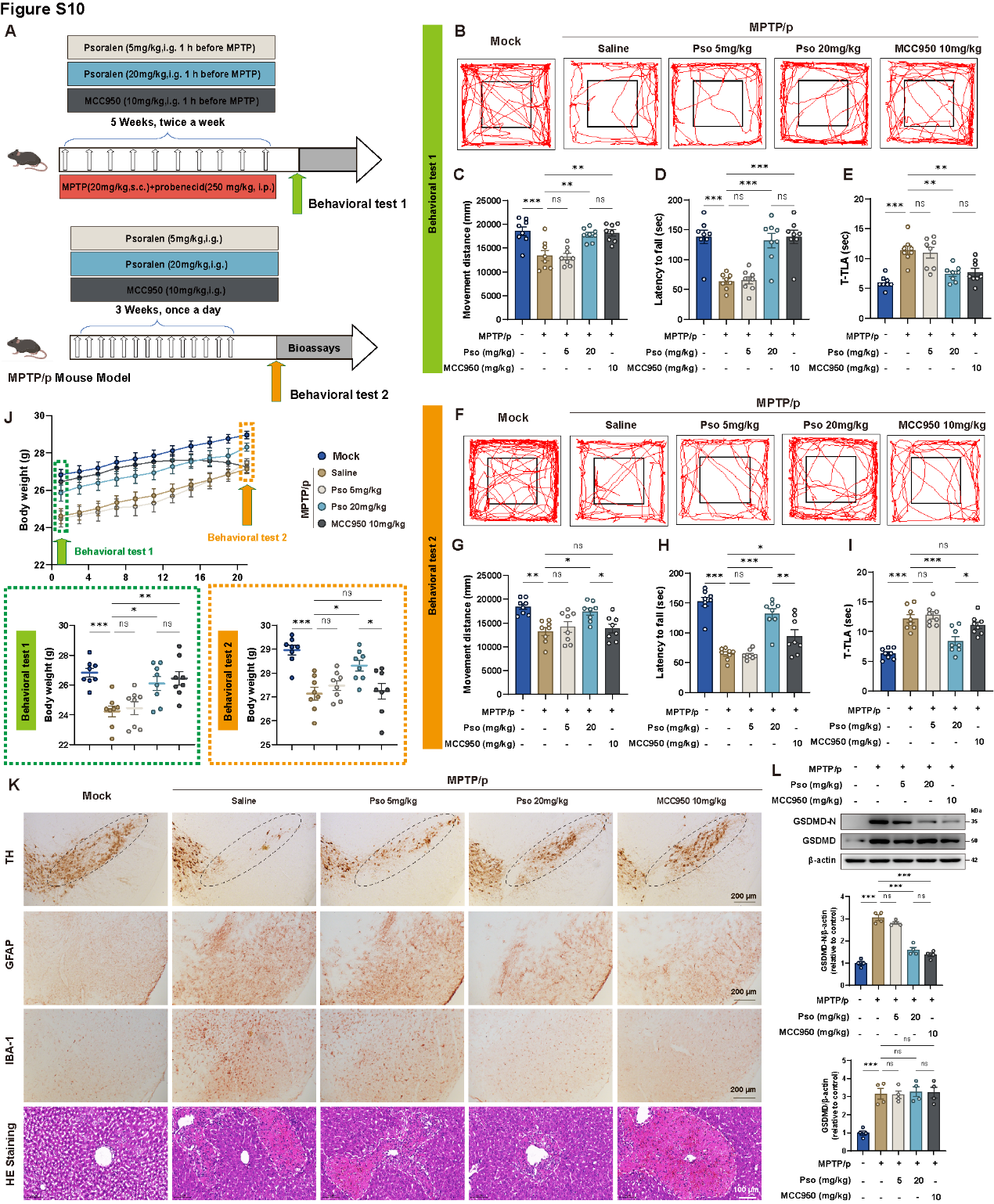


**Figure S10. Longitudinal functioning of psoralen.**

**(A)** Schematic diagram of the longitudinal experimental procedure included two stages. After Stage 1 chronic MPTP/p treatment (5 weeks), behavioral test 1 (green) was performed to assess the successful PD modeling and efficacy of Pso and MCC950. Then mice were administrated for another 3 consecutive weeks as Stage 2 following the behavioral test 2 (orange). At the endpoint of Stage 1, a series of behavioral tests (the travel path and movement distance in the open field test, the latency to fall in the rotarod test, and T-TLA in the pole test) were measured (**B-E**). At the endpoint of Stage 2, these behavioral tests were performed (**F-I**). n=8. (**J**) The body weight curve of mice during the longitudinal experiment. Enlarge: the body weight of mice at the endpoint of Stage 1 and Stage 2. (**K**) Representative immunohistochemical staining of TH^+^ neurons, GFAP, and IBA-1 in the midbrain, hematoxylin & eosin (H&E) staining in the liver, n=4. (**L**) Levels and quantification of GSDMD and N-terminal GSDMD in the midbrain were analyzed by immunoblotting. n=4. Data were analyzed by one-way ANOVA, followed by Tukey post-tests. *P < 0.05, **P < 0.01, and ***P < 0.001. ns: no significance.


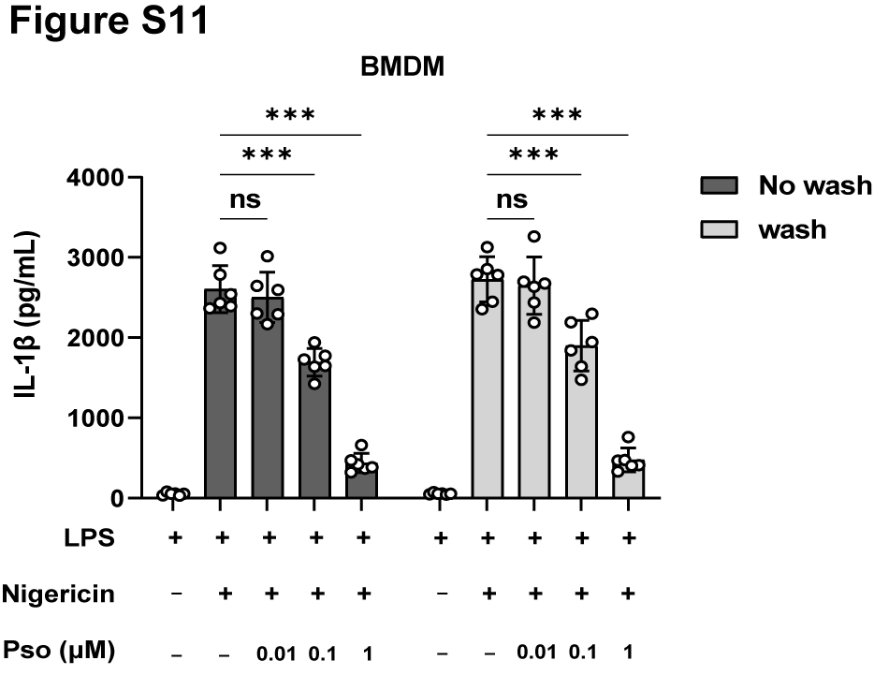


**Figure S11. The inhibitory effect of Pso is not reversible.**

ELISA of IL-1β in supernatants from LPS/Nigericin primed BMDMs that treated Pso (0.01, 0.1, 1 μM) for 1 h and washed 3 times, then left stimulated with nigericin. n=6. Statistics were analyzed using two-way ANOVA, followed by Šídák's multiple comparisons test. ns, not significant. ****P*<0.001.


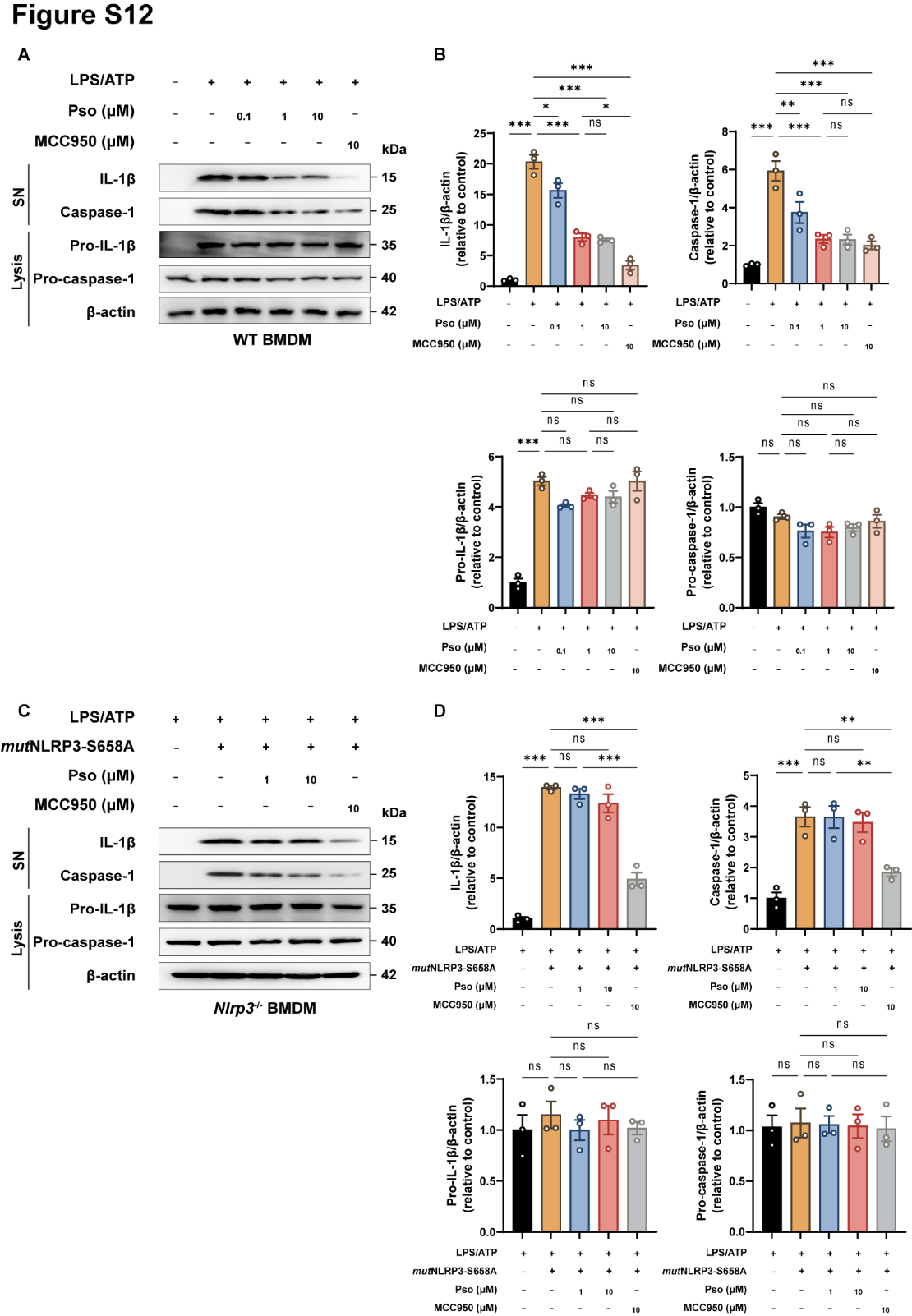


**Figure S12. The relative efficacy of Pso and MCC950.**

**(A-B)** Levels and quantification of IL-1β and caspase-1 in SN and levels of pro-IL-1β/pro-caspase-1 in Lysates were analyzed by immunoblotting in WT BMDMs pretreated with different concentrations of Psoralen (0.1, 1.0, and 10.0 μM) and MCC950 (10.0 μM) followed by stimulation with LPS/ATP. **(C-D)** Levels and quantification of IL-1β and caspase-1 in SN and levels of pro-IL-1β/pro-caspase-1 in Lysates were analyzed by immunoblotting in NLRP3 S658A mutant BMDMs pretreated with different concentrations of Psoralen (1.0, and 10.0 μM) and MCC950 (10.0 μM) followed by stimulation with LPS/ATP. n=3. Statistics were analyzed using one-way ANOVA, followed by Tukey's multiple comparisons test. *P < 0.05, **P < 0.01, and ***P < 0.001. ns: no significance.


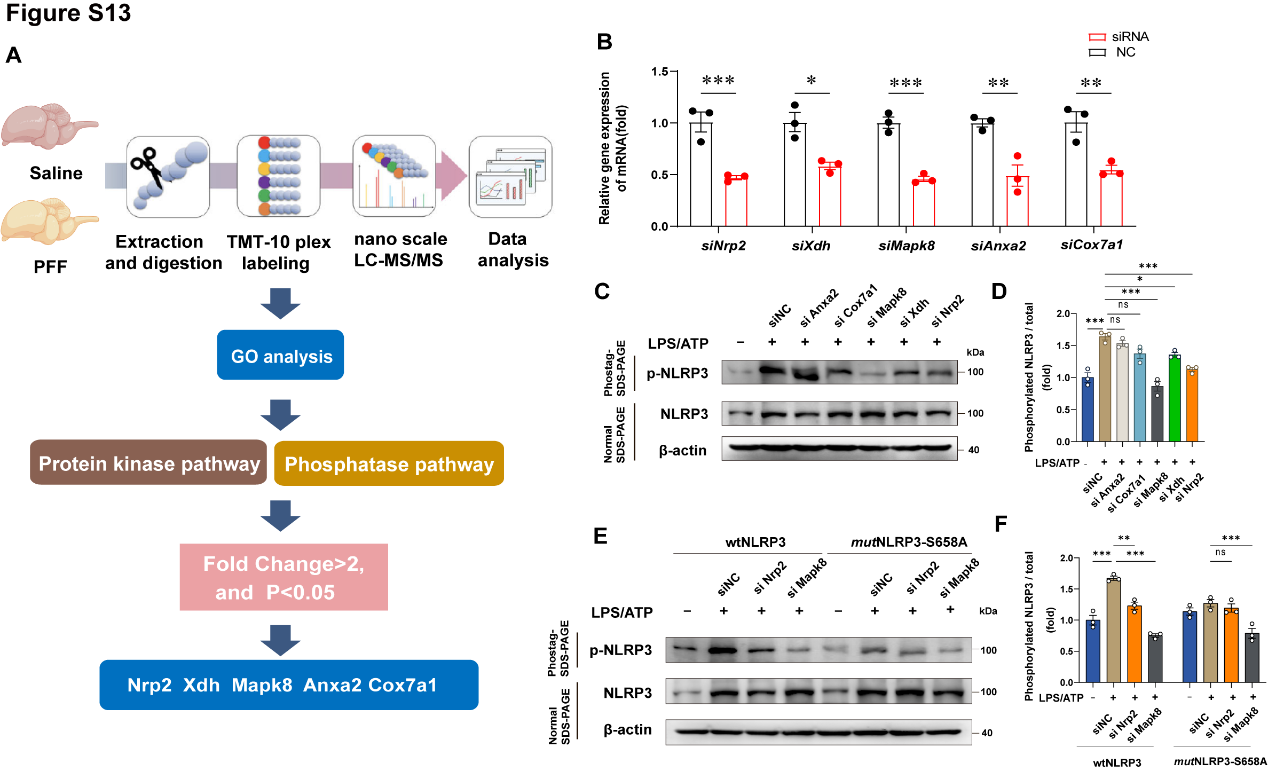


**Figure S13. Potential protein kinase or phosphatase regulating the S658 site of NLRP3 phosphorylation.**

**(A)** Schematic diagram of the experimental proteomics procedure. **(B)** The efficiency of small interfering RNA (*Nrp2*, *Xdh*, *Mapk8*, *Anxa2*, and *Cox7a1)* is validated by RT-PCR in BMDMs. **(C-D)** Phos-tag SDS-PAGE and quantification of the phosphorylation levels of NLRP3 were detected while these potential genes in protein kinase or phosphatase pathways (*Nrp2*, *Xdh*, *Mapk8*, *Anxa2*, and *Cox7a1*) were knockdown followed by stimulation with LPS/ATP in BMDMs. **(E-F)** Phos-tag SDS-PAGE and quantification of the phosphorylation levels of NLRP3 were detected while *Nrp2* and *Mapk8* were knockdown followed by stimulation with LPS/ATP in NLRP3 WT and the NLRP3 S658A mutant BMDMs. (B) Data were analyzed by Unpaired t-test. *P < 0.05, **P < 0.01, and ***P < 0.001. n=3. (D, F) Statistics were analyzed using one-way ANOVA, followed by Tukey's multiple comparisons test. *P < 0.05, **P < 0.01, and ***P < 0.001. ns: no significance. n=3.


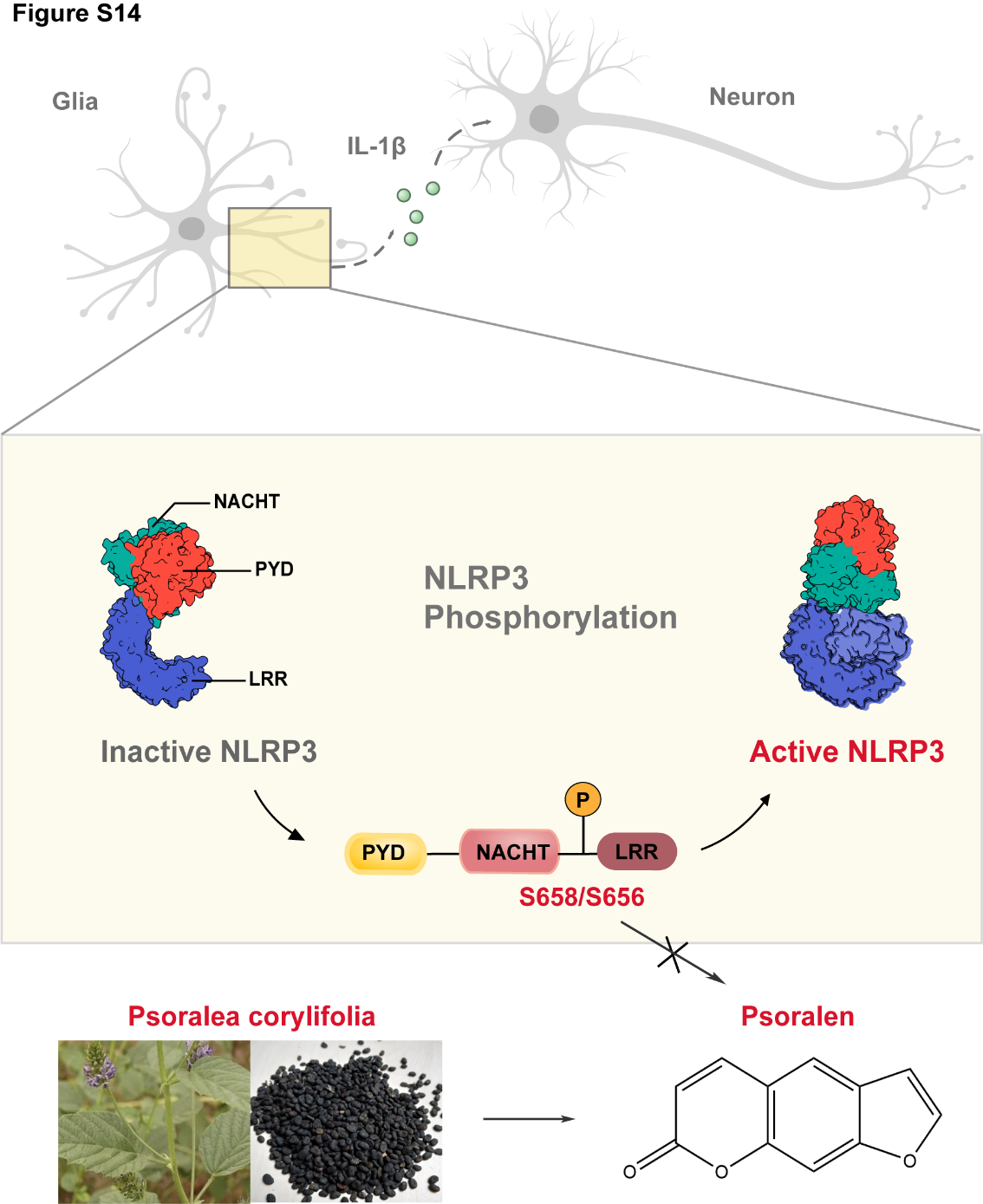


**Figure S14.** Psoralen from *Psoralea corylifolia* impedes NLRP3 phosphorylation at Serine 658/656 to attenuate NLRP3 inflammasome-mediated neuroinflammation.

**Supplementary Tables**

**Table 1.** Docking score of Natural Products.

| **Number** | **Docking score** | **Compound name** |
| --- | --- | --- |
| S2349 | 1.740 | Rutecarpine |
| S3846 | 0.765 | Eupatilin |
| S4737 | 0.746 | Psoralen |
| S3890 | 0.725 | Leonurine |
| S5135 | 0.627 | Hydroferulic acid |

**Table 2.** List of reagents used for Western blot, immunofluorescent, and immunohistochemistry.

| **Regents** | **Format** | **Source** | **#Catalog** |
| --- | --- | --- | --- |
| Anti-GFAP | Mouse IgG | Millipore | MAB360 |
| Anti-TH | Mouse IgG | Millipore | MAB318 |
| Anti-IL-1β | Goat IgG | Sigma Aldrich | I-3767 |
| Anti-Caspase-1 | Mouse IgG | AdipoGen | AG-20B-0042 |
| Anti-NLRP3 | Mouse IgG | AdipoGen | AG-20B-0014-C100 |
| Anti-ASC | Mouse IgG | Santa Cruz | SC-514414 |
| Anti-MAP2  Anti-IBA1  Anti-FLAG | Mouse IgG  Goat IgG  Mouse IgG | Proteintech  Abcam  CST | 17490-1-AP  AB5076  14793 |
| Anti-NEK7 | Rabbit IgG | Abcam | ab133514 |
| Anti-Beta Actin | Mouse IgG | Proteintech | 66009-1-Ig |
| Donkey anti-mouse | Alexa Fluor 488 | Invitrogen | A-21202 |
| Donkey anti-rabbit | Alexa Fluor 555 | Invitrogen | A-31572 |
| Donkey anti-rabbit | Alexa Fluor 647 | Invitrogen | A-31573 |
| Goat anti-mouse | Alexa Fluor 555 | Invitrogen | A32727 |
| Goat anti-rabbit | Alexa Fluor 488 | Invitrogen | A-11008 |
| Goat anti-mouse | PolyHRP | HISTOV | PHGM30 |
| Goat anti-rabbit | PolyHRP | HISTOV | PHGR30 |
| Donkey Serum |  | Sigma Aldrich | D9663 |
| Goat Serum |  | Sigma Aldrich | G9023 |
| DAPI |  | Sigma Aldrich | D9542 |
| Lipopolysaccharides (LPS) | From E. coli O55:B5 | Medchemexpress | HY-D1056 |
| Adenosine 5'-triphosphate (ATP) |  | Medchemexpress | HY-B2176 |
| Nigericin |  | Medchemexpress | HY-127019 |
| MCC950 |  | Medchemexpress | HY-12815 |
| Rutecarpine |  | Selleck | S2349 |
| Eupatilin |  | Selleck | S3846 |
| Psoralen |  | Selleck | S4737 |
| Leonurine |  | Selleck | S3890 |
| Hydroferulic acid |  | Selleck | S5135 |
| Phosbind Acrylamide |  | APExBIO | F4002 |
| MPTP Hydrochloride |  | Sigma Aldrich | M0896 |
| Probenecid |  | ALADDIN-E | 57-66-9 |

**Table 3.** List of plasmids used for immunoblotting.

| **Plasmids** | **Source** | **#Catalog** |
| --- | --- | --- |
| Flag-NLRP1 | Transheep Bio | TSB308093-1 |
| Flag-AIM2 | Hanbio | pHBLP002226 |
| Flag-NLRP3 | PPL | PPL00151-2b |
| Flag-NLRP3 S658A | PPL | PPL00151-2j |
| Flag-NLRC4 | PPL | PPL01760-2a |
| Flag-NLRP3（PYD 1-93aa） | PPL | PPL00151-2f |
| Flag-NLRP3（NACHT 220-536aa） | PPL | PPL00151-2g |
| Flag-NLRP3（LRR 742-991aa） | PPL | PPL00151-2h |

**Table 4.** The sequences of QT-PCR primers and siRNA were used.

| **Primer name** | **Sequence** |
| --- | --- |
| *m-Nrp2* | Forward: GGTGAAGATTGGATGGTCTACCG |
|  | Reverse: TGAACCGAGTCAGCAGTGGCAT |
| *m-Xdh* | Forward: GCTCTTCGTGAGCACACAGAAC |
|  | Reverse: CCACCCATTCTTTTCACTCGGAC |
| *m-Mapk8* | Forward: CGCCTTATGTGGTGACTCGCTA |
|  | Reverse: TCCTGGAAAGAGGATTTTGTGGC |
| *m-Anxa2* | Forward: CACCAACTTCGATGCTGAGAGG |
|  | Reverse: GCACATTGCTGCGGTTTGTCAG |
| *m-Cox7a1* | Forward: AAACCGTGTGGCAGAGAAGCAG |
|  | Reverse: CCCAAGCAGTATAAGCAGTAGGC |
| *m-Gapdh* | Forward: ACAGCAACAGGGTGGTGGAC |
| *Negative control* | Reverse: TTTGAGGGTGCAGCGAACTT  Forward: UUCUCCGAACGUGUCACGUTT  Reverse: ACGUGACACGUUCGGAGAATT |
| *m-siNrp2* | Forward: GGUGAAGAUUGGAUGGUCUTT |
|  | Reverse: AGACCAUCCAAUCUUCACCTT |
| *m-siXdh* | Forward: GCAUCGUCAUGAGUAUGUATT |
|  | Reverse: UACAUACUCAUGACGAUGCTT |
| *m-siMapk8* | Forward: GCAGAAGUAAACGUGACAATT |
|  | Reverse: UUGUCACGUUUACUUCUGCTT |
| *m-siAnxa2* | Forward: AGGGUGACGUUAGCAUUACTT |
|  | Reverse: GUAAUGCUAACGUCACCCUCA |
| *m-siCox7a1* | Forward: ACUGCCUACUGCUUAUACUTT |
|  | Reverse: AGUAUAAGCAGUAGGCAGUTT |
